# Guidelines for diagnosis and treatment in neurology – Lyme neuroborreliosis

**DOI:** 10.3205/000349

**Published:** 2025-10-09

**Authors:** Sebastian Rauer, Stefan Kastenbauer, Rick Dersch, Heidelore Hofmann, Volker Fingerle, Hans-Iko Huppertz, Klaus-Peter Hunfeld, Andreas Krause, Bernd Salzberger, Consensus group

**Affiliations:** 1 German Society of Neurology (DGN), Berlin, Germany; 2 Department of Neurology and Neurophysiology, Medical Center – University of Freiburg, Germany; 3 German Dermatology Society (DDG), Berlin, Germany; 4 German Society for Hygiene and Microbiology (DGHM), Münster, Germany; 5 German Society of Paediatrics and Adolescent Medicine (DGKJ), Berlin, Germany; 6 German Society for Paediatric Infectious Diseases (DGPI), Berlin, Germany; 7 German Society for Clinical Chemistry and Laboratory Medicine (DGKL), Bonn, Germany; 8 INSTAND e.V., Düsseldorf, Germany; 9 German Society for Rheumatology and Clinical Immunology (DGRh), Berlin, Germany; 10 German Society for Infectious Diseases (DGI), Berlin, Germany

**Keywords:** Borrelia burgdorferi infection, Lyme borreliosis, Lyme disease, Bannwarth syndrome, lymphocytic meningoradiculitis, facial nerve paresis, polyradiculitis, meningitis, encephalomyelitis, Lyme encephalopathy, polyneuropathy, tick-borne borreliosis

## Abstract

Lyme disease is the most common tick-borne infectious disease in Europe. Neurological manifestations occur in 3–15% of infections and can present as polyradiculitis, meningitis, and rarely as encephalomyelitis. The disease is treatable with antibiotics. The S3 guideline “Neuroborreliosis” has been updated in accordance with the methodological standards of the “Arbeitsgemeinschaft der Wissenschaftlichen Medizinischen Fachgesellschaften e. V.” (AWMF register number 030/071). Eighteen AWMF member societies, the Robert Koch Institute, the “Paul-Ehrlich-Gesellschaft für Infektionstherapie”, the “Schweizerische Neurologische Gesellschaft”, the “Österreichische Gesellschaft für Neurologie“, the “Deutsche Borreliose-Gesellschaft” and two patient organizations were involved in the update. The guideline aimed at physicians in practice and hospital settings who are involved in the treatment of neuroborreliosis in children and adults. For the first time, there is Class Ia evidence that a 14-day course of antibiotics is therapeutically sufficient in early neuroborreliosis. Additionally, it is now recommended that the administration of steroids alongside antibiotic therapy is not advised in cases of facial palsy within the context of a neuroborreliosis that is probable or confirmed according to diagnostic criteria. The age limit for doxycycline in the treatment of neuroborreliosis in children under 8 years has been removed. There are still no valid study data on the effectiveness of combination antibiotic treatments. A systematic review on the therapy of so-called Post-Treatment Lyme Disease Syndrome (PTLDS) showed that parameters such as quality of life, fatigue, depression, and cognition do not respond to antibiotic therapy.

## Abstract (de)

Die Lyme-Borreliose ist die häufigste durch Zecken übertragene Infektionskrankheit in Europa. Eine neurologische Manifestation kommt bei 3–15% der Infektionen vor und kann sich als Polyradikulitis, Meningitis sowie selten als Enzephalomyelitis manifestieren. Die Erkrankung ist durch Antibiotika behandelbar. Die bisher gültige S3-Leitlinie „Neuroborreliose“ wurde entsprechend den methodischen Vorgaben der Arbeitsgemeinschaft der Wissenschaftlichen Medizinischen Fachgesellschaften e. V. (AWMF-Registernr. 030/071) aktualisiert. An der Aktualisierung waren 18 AWMF-Mitgliedsgesellschaften, das Robert Koch-Institut, die Paul-Ehrlich-Gesellschaft für Infektionstherapie, die Schweizerische Neurologische Gesellschaft, die Österreichische Gesellschaft für Neurologie, die Deutsche Borreliose-Gesellschaft und 2 Patientenorganisationen beteiligt. Die Leitlinie richtet sich an Ärzte in Praxis und Klinik, die mit der Behandlung der Neuroborreliose bei Kindern und Erwachsenen befasst sind. Erstmals liegt jetzt eine Klasse-Ia-Evidenz vor, dass eine 14-tägige Dauer der Antibiotikagabe bei früher Neuroborreliose therapeutisch ausreichend ist. Neu ist außerdem, dass eine Steroidgabe zusätzlich zur Antibiotikatherapie bei Fazialisparese im Rahmen einer entsprechend den Diagnosekriterien wahrscheinlichen oder gesicherten Neuroborreliose nicht empfohlen wird und dass die Altersbegrenzung für Doxycyclin bei Kindern unter 8 Jahren zur Therapie der Neuroborreliose entfällt. Über die Wirksamkeit von Antibiotika-Kombinationsbehandlungen liegen weiterhin keine validen Studiendaten vor. Im Rahmen eines systematischen Reviews zur Therapie des sogenannten Post-Treatment Lyme Disease Syndrome (PTLDS) zeigte sich, dass die untersuchten Parameter Lebensqualität, Fatigue, Depression und Kognition nicht auf eine antibiotische Therapie ansprechen. Für die Wirksamkeit anderer Therapieverfahren fanden sich keine aussagekräftigen Studien.

## Preamble

This guideline pertains to the diagnosis and treatment of neurological manifestations of Lyme borreliosis in children and adults. The guideline also deals with aspects of chronic, non-specific symptoms associated with Lyme borreliosis, which are also subsumed under such terms as “post-treatment Lyme disease syndrome” (PTLDS), “chronic neuroborreliosis” and “Lyme encephalopathy” without a clear distinction being made between them. 

Twenty-two medical societies, 18 of which were members of the AWMF, the Robert Koch Institute and two patient organisations participated in its development. A systematic search and assessment of the literature for the first version of the S3 guideline “Lyme Neuroborreliosis” was carried out by the German Cochrane Centre Freiburg (Cochrane Germany) with significant input from RD. A systematic search and assessment of the literature for the updated guideline were again carried out by RD. 

### What’s new?

**1. Statement (reviewed in 2023)**

- *The previous S3 guideline “Lyme Neuroborreliosis” (AWMF register no. 030/071) was updated in accor* *d* *a* *nce with the methodological specifications of the Association of Scientific Medical Societies (AWMF). *
- *The update now includes the diagnosis and treatment of Lyme neuroborreliosis in children. *
- *There are still no analysable study data on the efficacy of combination antibiotic treatment. *
- *There are still no study data on the efficacy of chloroquine, carbapenems and metronidazole.*

**2. Statement (new as of 2023)**

*A prospective, randomised clinical trial on the length of antibiotic treatment for early Lyme neuroborreliosis has now been published. It has found that treatment with doxycycline for 6 weeks provided no extra clinical benefit compared to treatment for 2 weeks *[1]*. According to a pooled analysis with data from an earlier study *[2]*, there is now level 1a evidence for the length of antibiotic treatment for early Lyme neuroborreliosis (section 5.2.1).*

Level Ia evidence

**3. Statement (new as of 2023)**

*A systematic review of the treatment for post-treatment Lyme disease syndrome (PTLDS) was carried out for the first time as part of the development of the guideline. It found that the analysed parameters quality of life, fatigue, depression and cognition did not respond to antibiotic treatment. There are currently no conclusive studies on the effectiveness of other treatment methods (section 4.3.5).*

Level Ib evidence

**15. Recommendation (new as of 2023)**

*Steroids should not be administered alongside antibiotics to treat facial nerve paresis if there is probable or confirmed Lyme neuroborreliosis as per the diagnostic crite* *r* *i* *a (see section 3.10).*

[3]*, *[4]*, *[5]

Grade of recommendation ↓↓

Level III evidence

Level of consensus 100% (17/17)

**4. Statement (new as of 2023)**

*Restrictions have been lifted on administering doxycycline to children under the age of 8 as part of the treatment of Lyme neuroborreliosis. Recent data show that this does not lead to a yellowing of the teeth *[6]*, *[7]*, *[8]*, *[9]*, *[10]* (section 5.5).*

Level IIIb evidence

### Key recommendations at a glance

**8. Statement (reviewed in 2023)**

*The suspected clinical diagnosis of Lyme neuroborreliosis (cranial nerve deficits, meningitis/meningoradiculitis,* *encephalomyelitis) can be confirmed by the detection of i* *n* *flammatory changes in the cerebrospinal fluid in con* *junc* *tion with a borrelia-specific intrathecal antibody synthesis.*

[11]*, *[12]*, *[13]*, *[14]*, *[15]

Level III evidence

Level of consensus: 100% (14/14)

**3. Recommendation (reviewed in 2023)**

*Serology testing should only be ordered if there is sufficient clinical suspicion.*

EC

Level of consensus: 100% (16/16) 

**24. Recommendation (reviewed in 2023)**

*Early* *Lyme neuroborreliosis should be treated with one of the following antibiotics: doxycycline, ceftriaxone, cefotaxime, penicillin G.*

[2]*, *[16]*, *[17]*, *[18]*, *[19]*, *[20]*, *[21]*, *[22]

Grade of recommendation ↑↑

Level IIb evidence

Level of consensus: 94% (16/17 yes, 1 abstention) 

**25. Recommendation (reviewed in 2023)**

*Late* *Lyme neuroborreliosis should be treated with one of the following antibiotics: doxycycline, ceftriaxone, cefotaxime, penicillin G.*

*Section 5.3, *[16]*, *[23]*, *[24]*, *[25]*, *[26]

Grade of recommendation ↑↑

Level IV evidence

Level of consensus: 88% (15 yes, 2 no)

**5. Statement (modified in 2023)**

*Doxycycline and beta-lactam antibiotics show similar efficacy and safety in regard to neurological symptoms in early Lyme neuroborreliosis *[16]*, *[17]* (section 5.2.2).*

Level Ia evidence

Level of consensus: 100% (17/17) 

**18. Recommendation (modified in 2023)**

*Antibiotic treatment for* *early* *Lyme neuroborreliosis should be carried out over a period of 14 days.*

[1]*, *[2]*, *[16]

Grade of recommendation ↑

Level I evidence

Level of consensus: 100% (17/17) 

**19. Recommendation (modified in 2023)**

*Antibiotic treatment for* *late* *Lyme neuroborreliosis should be carried out over a period of 14–21 days.*

*See section 5.3, *[16]*, *[23]*, *[24]*, *[25]*, *[26]

Grade of recommendation ↑

Level IV evidence

Level of consensus: 94% (16 yes, 1 no) 

**26. Recommendation (reviewed in 2023)**

*Treatment success should be based on clinical symptoms.*

Grade of recommendation ↑↑

EC

Level of consensus: 100% (17/17)

**6. Statement (modified in 2023)**

*A systematic review found that the apparently high prevalence of persistent, non-specific, or atypical symptoms following a presumed case of Lyme neuroborreliosis, as reported in many studies, is largely attributable to methodological biases resulting from unclear case definitions *[27]*.*

Section 4.1

Level Ia evidence

Level of consensus: 88% (15 yes, 2 no) 

### Preface

Lyme borreliosis is the most common tick-borne infectious disease in Europe. A neurological manifestation occurs in 3–15% of infections and can manifest as polyradiculitis, meningitis and (rarely) encephalomyelitis. The disease can be treated with antibiotics. 

### Target group

This guideline is directed at physicians in private practices and clinics specialising in various fields of medicine who are directly or indirectly involved in treating Lyme neuroborreliosis in children and adults. The wide range of interdisciplinary medical fields dealing with Lyme neuroborreliosis is reflected in the number of medical societies involved and in the participation of the Robert Koch Institute (see the Guideline Report in Attachment 1 ). The guideline also acts as a source of information for patients and others interested in Lyme neuroborreliosis. These groups are represented in the consensus process by mandate holders from two patient and/or interest organisations (see the Guideline Report in Attachment 1). 

### Guideline objectives (recommendations)

- Definition of the disease
- Confirmation of the clinical diagnosis
- Differentiation of non-specific symptoms
- Antibody testing in serum
- Cerebrospinal fluid (CSF) testing including antibody detection in CSF
- Effective use of molecular testing and culture tests
- Treatment
- Differential diagnosis
- Prevention
- Observation of the tick bite; information sheet for patients
- Diseases caused by relapsing fever Borrelia (Borrelia miyamotoi and Borrelia recurrentis) are not covered in this guideline.
- Questions relating to co-infections linked to tick-borne diseases are not covered in this guideline.

## 1 Epidemiology, transmission, manifestations, prophylaxis

### 1.1 Epidemiology

#### 1.1.1 Definition

Lyme borreliosis is a multi-systemic inflammatory disease caused by an infection with spirochetes from the *Borrelia burgdorferi* sensu lato complex. These are transmitted in Germany through the bite of the Ixodes ricinus tick.

#### 1.1.2 Distribution and species

Lyme borreliosis is the most common vector-borne disease in the temperate climate zones of the northern hemisphere and is endemic. In North America, Lyme borreliosis is caused exclusively by the Borrelia species *Borrelia burgdorferi* sensu stricto, while in Europe *B. afzelii*, *B. bavariensis* and *B. garinii* have also been found to be pathogenic to humans. In addition, the newly identified species *Borrelia spielmanii* has the potential of being pathogenic to humans. It has been detected in 4 out of 160 skin isolates (all from erythema migrans), but has so far not been linked to Lyme neuroborreliosis in Germany (72 CSF isolates) [28]. The pathogenic potential of the various *Borrelia burgdorferi* species varies [29]. After *B. garinii* OspA type 4 was classified as a new species of *Borrelia bavariensis* [30], a re-evaluation of 242 human isolates from Germany [28] revealed 21% of the 72 CSF isolates were *B. afzelii*, 22% *B. bavariensis* and 29% *B. garinii*. Of the 160 skin isolates, 67% were *B. afzelii*, 12% *B. bavariensis* and 12% *B. garinii*; i.e, only the skin isolates showed a clear prevalence of one species, namely *B. afzelii*.

No reliable figures are currently available on the incidence of Lyme borreliosis in the individual European countries. An analysis of reporting registries from six eastern German federal states found a strongly fluctuating incidence of 0.5 cases per 100,000 inhabitants depending on the region, compared to 138 cases per 100,000 inhabitants in the period from 2013 to 2017 [31]. Secondary data analyses of health insurance data on the basis of the ICD-10 coding A 69.2 (G) found a significantly higher incidence of 179 per 100,000 inhabitants with area-dependent fluctuations up to a factor of 16 (40–646 per 100,000 inhabitants) [32]. An earlier study using a similar methodology arrived at even higher case numbers, although the authors do not rule out the possibility that their case numbers were overestimated due to clinical misdiagnoses or miscoding [33].

In summary, the available epidemiological data are insufficient for drawing definitive conclusions. Data published to date in Germany suggest an incidence of Lyme borreliosis of between 60,000 to >200,000 cases/year. 

#### 1.1.3 Frequency of different manifestations

According to a survey on reportable manifestations of Lyme borreliosis in nine federal states in Germany, acute neuroborreliosis (2.7%) is the second most common clinical manifestation after erythema migrans (95%). This is followed by Lyme arthritis (2.1%) [31]. In a prospective, population-based study conducted in the Würzburg area, 313 cases of Lyme borreliosis were identified over a 12-month period, corresponding to an incidence of 111 per 100,000 inhabitants. This resulted in the following manifestation rates [34].

**Early manifestations:**

- 89% erythema migrans (erythema migrans linked to another organ manifestation in a further 3% of cases)
- 3% Lyme neuroborreliosis (stage II)
- 2% Borrelial lymphocytoma
- <1% carditis

**Late manifestations:**

- 5% Lyme arthritis
- 1% acrodermatitis chronica atrophicans
- Late Lyme neuroborreliosis (stage III) was not identified.

According to one study, children have a higher risk of developing Lyme neuroborreliosis after a tick bite than adults, most likely because they are more frequently bitten on the head [35].

#### 1.1.4 Seroprevalence of Borrelia-specific antibodies

Borrelia-specific antibodies are found in 3–20% of healthy individuals in Germany and Austria, depending on the endemic region and age group [36], [37], [38], [39]. A seroprevalence of 20% was found in 964 (asymptomatic) Swiss orienteers; in asymptomatic blood donors this was 8% [40]. A cross-sectional German study of children and adolescents ranging in age from 1 to 17 years found an average seroprevalence of 4.8%. The relative probability of a positive antibody result was age-dependent and increased for every year of life by 6% in girls and by 11% in boys [41]. An elevated level of borrelia-specific IgG antibodies were found in 20% of men over the age of 60 [38].

#### 1.1.5 Rates of infected ticks

Studies of ticks in southern Germany revealed average infection rates of around 1% for larvae, 10% for nymphs and 20% for adults [42]. In addition to regional differences in rates of infected ticks (18–37% of adults and 5–12% of nymphs), there were also clear differences in the regional distribution of Borrelia species [28]. Infection rates in Switzerland were 5–7% depending on the region [43]. The population density of infected ticks also varies greatly from region to region, ranging from 2 to 58 per 100 m^2^ in Switzerland. Across Europe, an analysis of publications from 24 countries revealed an average rate of infected ticks of 14.2% (range: 3.1–38.1%) [44]. In addition to Lyme borreliosis, ticks can transmit other infectious diseases including tick-borne encephalitis (TBE), human granulocytic anaplasmosis and rickettsiosis etc. 

##### Summary

- Lyme borreliosis is a multisystem disease that is transmitted through the bite of the Ixodes ricinus tick. It primarily affects the skin, nervous system and joints.
- Five species have so far been identified in Europe that are pathogenic to humans.
- There are no reliable figures on incidence (incidence from various surveys in Germany ranges from 60,000 to >200,000/year).
- The seroprevalence of Borrelia-specific antibodies is 3–20% and depends on region and age.
- Rates of infected ticks are area-dependent: 3–38% of adults, 5–12% of nymphs, 1% of larva.

### 1.2 Route of infection

Borrelia are transmitted through the bite of hard-bodied ticks (in Europe by the “castor bean tick” Ixodes ricinus). According to data from animal experiments, the risk of infection increases the longer blood meal. It is not possible to derive from current data the earliest point in time that an infection can occur, especially as the probability of transmission also appears to vary depending on the species [45]. The transmission mechanism of the Borrelia that survive in the tick’s intestine before the blood meal is very complex [46]. According to German studies, a seroconversion can be expected in 2.6–5.6% of those who have been bitten by a tick and disease will manifest in 0.3–1.4% [47], [48], [49]. A study conducted in western Switzerland found that the risk of becoming infected with Borrelia from a tick bite was just under 5% [50].

### 1.3 Prophylaxis

(Cited from the DDG S2k guideline “Cutaneous Lyme Borreliosis”; AWMF Register No. 013/044 [51].)

#### 1.3.1 Preventing Lyme borreliosis

It is very important to **remove ticks early**, before they have become engorged. The risk of Borrelia transmission increases with the length of time that the tick sucks [52]. Transmission in the first 12 hours was rarely observed in laboratory animals. After spending time in nature (garden, park, field, forest and meadows etc.) where contact with ticks is possible, the body should be checked for ticks that same evening; the head and neck of children should receive particular attention. 

Ticks should be removed immediately using tick tweezers, a tick card or suitable tools in order to reduce the likelihood of a Borrelia transmission. If a suitable tool is not available, some authors recommend grasping hold of the animal between the thumb and forefinger without crushing it, then carefully pulling the animal vertically away from the skin, stretching out the skin, and then waiting up to 90 seconds until the tick releases itself. If parts of the sucking apparatus remain in the skin, they can be removed later using a needle or curettage [53]. If the head or the sucking apparatus is left in the skin, this poses no danger with regard to the transmission of Borrelia. The bodies of fully engorged nymphs and adult ticks should not be squeezed. Examining the removed tick for Borrelia is not recommended, as the detection of Borrelia in the tick is not sufficiently predictive of the transmission of Borrelia to the host and the development of disease. After the tick has been removed, the patient should be instructed to **observe the bite site** for the next 6 weeks (Appendix 6: Patient information after a tick bite in Attachment 2). 

#### 1.3.2 Prophylactic treatment after a tick bite

According to an American study, the risk of infection can be reduced by taking a one-time, 200 mg prophylactic dose of doxycycline after a tick bite (87% efficacy) [54], [55]. However, the results should be interpreted with caution, as only one follow-up was carried out after 6 weeks. Therefore, no conclusions can be drawn as to whether this is effective for late-stage infections.

According to a meta-analysis, a single 200 mg prophylactic dose of doxycycline after a tick bite is effective (relative risk of 0.29 (95% CI: 0.14–0.60)); in contrast, neither prophylactic treatment with antibiotics over 10 days (amoxicillin, penicillin or tetracycline) nor a local prophylactic application of azithromycin were found to effective [55]. The authors conclude that 50 prophylactic treatments (95% CI: 25–100) are necessary to prevent one infection. 

In view of the low risk of infection, a large number of unnecessary doxycycline doses would have to be administered in order to prevent one potential infection. Frequent administration of a prophylaxis could affect the intestinal flora, and the development of resistance is conceivable. For this reason, oral doxycycline prophylaxis is not recommended in Europe [56]. The prophylactic use of an antibiotic cream is also controversial. Animal studies with azithromycin cream show good prophylactic results [57], [58]. A placebo-controlled study on its effectiveness in humans showed no prophylactic effect [59]. Therefore, this prophylactic treatment is also not recommended. 

**Recommendations for preventing infection**

(Taken from the S2k guideline “Cutaneous Lyme Borreliosis”, AWMF Register No. 013/044, [51].)

- *To prevent tick bites, clothing should be worn that covers the body. *
- *The use of tick repellents can be recommended to a limited degree. *
- *After spending time outdoors where contact with ticks is possible, the skin should be checked for ticks no later than that evening. *
- *Ticks should be removed early to prevent Lyme borreliosis. *
- *The site of the bite should be observed for up to six weeks. *

**Not recommended**

- *The removed tick should not be analysed for Borrelia. *
- *Local or systemic prophylactic antibiotic treatment after a tick bite should not be carried out.*

(Consensus 80% (12 yes, 3 no))

#### 1.3.3 Vaccines

There is currently no vaccine that has been approved for use in humans.

A vaccine with recombinant lipidated Osp A has been tested in the USA as part of a major study and has shown to be effective [60], [61]. The vaccine was authorised in the USA in 1999, but was withdrawn from the market by the manufacturer in 2002. The reasons for this are not of a medical nature. Reports of adverse reactions to the vaccine in individuals with a genetic predisposition have been refuted by several qualified studies [62], [63], [64].

This monovalent vaccine is not suitable for use in Europe as it only protects against infection with *B. burgdorferi* sensu stricto and not against the genospecies *B. afzelii* and *B. garinii*, which are frequently found in Europe. A polyvalent OspA vaccine is currently being developed for Europe [65], but approval is not expected in the foreseeable future. A 6-valent outer surface protein A vaccine is currently being investigated for efficacy, safety and tolerability as part of a phase 3 clinical trial, which is scheduled to finish in late 2024 (ClinicalTrials.gov Identifier: NCT05477524). 

## 2 Symptoms

### 2.1 Possible stages

**Early localised stage:** An early Borrelia infection manifests in 80–90% of patients as local erythema migrans (early localised stage) [34], [66]. General symptoms such as feeling unwell, arthralgia, myalgia, subfebrile temperatures or night sweats may occur a few days to weeks after a Borrelia infection [67]. 

**Early disseminated stage:** A disseminated infection can occur weeks to months after a tick bite (erythema migrans is only reported in around 25–50% of the acute cases of Lyme neuroborreliosis [15], [23], [24]). This predominantly affects the nervous system, joints and heart [67].

**Late manifestations:** In rare cases, a late or chronic manifestation can occur after months or years with involvement of the skin, nervous system and joints [67], [68], [69], [70].

Information about tick bites reveals little about the time of infection, since unnoticed tick bites lead to infection in around two-thirds of all cases [15], [24], [71]. Therefore, in addition to the clinical picture, disease duration is being used more and more to classify Lyme neuroborreliosis [72].

### 2.2 Neurological manifestations in adults

**Garin-Bujadoux-Bannwarth syndrome (meningoradiculoneuritis)** is the most common manifestation of acute Lyme borreliosis in adults in Europe after erythema migrans [14], [15], [24]. 

In Europe, isolated **meningitis** (without radicular symptoms) is predominantly observed in children [24], [35], [73], [74], [75].

The symptoms of **radiculitis** develop on average 4–6 weeks (maximum 1–18) after the tick bite or after the erythema migrans [24], [76]. Segmental pain occurs first, which is worse and night and whose localisation can change. The pain is often initially localised in the extremity where the tick bite or erythema migrans had been observed [24], [77]. The pain has a burning, piercing, stabbing or tearing nature and responds only mildly to conventional analgesics. It often peaks within a few hours or days. Three-quarters of patients develop neurological deficits after 1–4 weeks, and pareses are more frequent than sensory disorders [24], [76].

Around 60% of patients with Bannwarth syndrome have **cranial nerve deficits**.

- All cranial nerves can be involved with the exception of the olfactory nerve.
- The facial nerve is affected in over 80% of cases where there is cranial nerve involvement [13], [24], whereby a bilateral manifestation is observed (in around 1/3 of all cases) [15], [24], [78]. The sense of taste may not be affected. In unilateral cases, it can be difficult to differentiate from idiopathic facial nerve paresis; in some cases, however, symptoms or patient history (e.g. erythema migrans, radicular pain) can help diagnose Lyme neuroborreliosis. CSF testing can provide clarity here. Total recovery is observed in most cases within 1–2 months regardless of the severity of the facial nerve paresis. Residual symptoms or partial recovery with facial synkinesis (pathological, unintentional movements) occur in around 5–10% of patients [78], [79], [80].
- Lyme neuroborreliosis can also affect the abducens nerve and very rarely the vestibulocochlear nerve, the optic nerve (optic neuritis, papilloedema), the cranial nerves (III and IV), the trigeminal nerve and the lower cranial nerves (IX–XII) [15], [24], [76], [81]. It is uncertain whether isolated damage to the vestibulocochlear nerve occurs in the context of an acute Borrelia infection.

**Polyneuropathy/polyneuritis** as an expression of a Borrelia infection is linked to acrodermatitis chronica atrophicans (ACA) in 20% of patients in Europe [82]. Isolated polyneuropathies/polyneuritis without other clear symptoms of Lyme borreliosis have been described in 39–52% of American patients with Lyme borreliosis [83], [84]. However, in 284 US patients with an aetiologically unexplained polyneuropathy, Lyme borreliosis was identified as the cause of the polyneuropathy in only one case (0.3%) following a diagnostic re-evaluation [85]. In contrast, there are only very few instances of polyneuropathy or polyneuritis in Europe with no link to ACA. No causal relationship can easily be made between neurological symptoms and a Borrelia infection in patients with polyneuropathy/polyneuritis whose blood has tested positive for Borrelia [86]. This is because Borrelia-specific antibodies are found in approx. 3–20% of healthy individuals, depending on the endemic region and age group [36], [38], [87]. Occupationally exposed risk groups, such as forestry workers, even have a seroprevalence of over 50% [88]. In such cases, the probability of a causal relationship depends on whether other clinical symptoms of Lyme borreliosis are present and whether other common causes of polyneuritis have been ruled out. 

An **involvement of the central nervous system** is rare and occurs in around 3.3% of Lyme neuroborreliosis cases (95% CI 2.2–4.4%) [15], [24], [26]. Its onset is gradual and it is often chronic. The most common manifestation is myelitis with a spastic-ataxic gait and bladder dysfunction [14], [24]. Symptoms can develop over days to several months. Some patients develop severe tetra- or paraparesis. Around 60% of patients with myelitis have additional signs of encephalitis and around 40% have cranial nerve involvement. Encephalitis has no clinical characteristics specific to the pathogen. 

Encephalitis can lead to **psychiatric symptoms** or **organic brain syndrome**. According to a systematic review in conjunction with a retrospective cohort study from Scandinavia (n=45/35 patients), the most common symptoms of encephalitis are reported to be: confusion 44%/71%, change in character 38%/49%, lethargy 9%/26%, somnolence 22%/8%, coma 18%/0%, memory loss and loss of concentration 27%/69%, aphasia 7%/26%, ataxia 24%/20%, hallucinations 11%/20%, dysarthria 20%/11%, apraxia 2%/8%, seizures 16%/11%, paresis 51%/31%. Concomitant symptoms are: headache 36%/63%, dizziness 11%/54%, fever 36%/23%, cranial nerve involvement 24%/28%, and radicular pain 22%/77% [26]. In addition, cases of acute psychosis [15], [89], [90], [91], [92], [93] or Tourette’s syndrome [94] have been reported, which show inflammatory CSF changes with pleocytosis and protein elevation as well as an increased Borrelia-specific AI. These regress under antibiotic treatment. 

In very rare cases, cerebral symptoms (e.g. strokes) are caused by a Borrelia-induced **vasculitis** [95], [96]. According to a non-systematic review, only 62 cases had been reported up to 2015 [95]. **Myositis** is another very rare manifestation of Lyme borreliosis, for which only individual case reports exist [97], [98]. Clinical symptoms include focal pain and paresis. 

### 2.3 Neurological manifestations in children

In Europe, **Lymphocytic meningitis** with **facial nerve paresis** (approx. 55%) and without (approx. 30%) is the most common manifestation of Lyme neuroborreliosis in children [24], [35], [73], [74], [99]. Facial nerve paresis caused by *B. burgdorferi* is usually accompanied by lymphocytic meningitis. The symptoms of meningitis are often very subtle and can be overlooked if there is no cranial nerve involvement [100]. The facial nerve and the nerves of the external eye muscles are most frequently affected. In principle, all cranial nerves can be affected with the exception of the olfactory nerve. Radicular symptoms in the spinal nerves are rare. However, early Lyme neuroborreliosis with myelitis [101], acute hemiparesis [102], opsoclonus myoclonus syndrome [103] and ataxia [104] has been reported. In some cases, erythema migrans can still be detected, sometimes also on the head or neck. **Late Lyme neuroborreliosis** is very rare in children. It is characterised by seizures, neurological deficits with paralysis and enuresis. Cognitive impairment and mood disorders can also occur [102].

### 2.4 Clinical course

**Early Lyme neuroborreliosis: **Symptoms can last weeks to months [14], [15], [24]

- Presumably more than 98% of Lyme neuroborreliosis cases [34], [75]
- Neurological symptoms appear several weeks to several months after a tick bite
- Typical manifestations: painful meningopolyradiculitis of the spinal nerves in connection with unilateral or bilateral facial paresis (Bannwarth syndrome); also meningitis in children
- Frequently: radicular pain

**Late Lyme neuroborreliosis** (also known as **chronic Lyme neuroborreliosis):** Symptoms last for months to years [14], [15], [24], [26]

- Presumably less than 3% of Lyme neuroborreliosis cases [26], [34], [75]
- The neurological symptoms develop gradually over months (to years)
- Typical manifestations: encephalomyelitis with spastic-ataxic gait disorder and bladder disorder, encephalitic symptoms with change in personality, confusion, cognitive impairment, impaired consciousness and epileptic seizures (see above)
- Isolated meningitis is very rare
- Rarely painful

A history of erythema migrans (EM) is reported by 34–46% of patients with Lyme neuroborreliosis [14], [15], [24].

### 2.5 Symptoms that should prompt a clarification of Lyme neuroborreliosis

(Hansen & Lebech [24]; Kaiser [14]; Oschmann et al. [15]) (Appendix 7 in Attachment 2)

- Radiculitis of the spinal nerves (typical for early stages) (frequency 70–75%): initially strong radicular or segmental pain, mostly at night, persisting for weeks if not treated, paresis develops into paraesthesia as the disease progresses
- Radiculitis of the cranial nerves II–XII (frequency 47–56%): fascial nerve paresis most frequent (83–92%), bilateral in around one-third of patients; ocular muscle paresis (abducens nerve) (frequency 4–9%). Very rare (individual case reports): paresis of the oculomotor and trochlear nerves, optic neuritis, papilloedema, acute sensorineural hearing loss, peripheral vestibulopathy (vestibulocochlear nerve), hypoglossal nerve paresis
- Meningitis (more common in children (frequency approx. 30%) than in adults (frequency 4–5%)): headache, meningism, photophobia, nausea, vomiting, fatigue, emotional instability; rarely chronic
- Neuritis of the peripheral nerves (extremely rare), most likely only in the context of acrodermatitis chronica atrophicans/axonal polyneuropathy with predominantly sensory symptoms
- Encephalitis (mostly late Lyme neuroborreliosis) (a systematic review indicates an encephalomyelitis rate of 3.3% (95% CI 2.2–4.4%) [26], 4–5% in older case series [15], [24]) paresis, speech and language disorders, coordination disorders, occasional epileptic seizures; organic brain syndrome with lack of concentration, loss of consciousness and hallucinations
- Myelitis (mostly late Lyme neuroborreliosis) (frequency, see encephalitis above): transverse disseminated sensory dysfunction, central and peripheral paresis, voiding dysfunction; frequently linked to encephalitis
- Borrelia-induced cerebral vasculitis: rare, mainly ischemic events in different areas of the bloodstream with corresponding neurological symptoms [95], [105]
- Borrelia-induced myositis: extremely rare [97], [98]

## 3 Diagnostic testing

### 3.1 Overview

**Figure 1:**
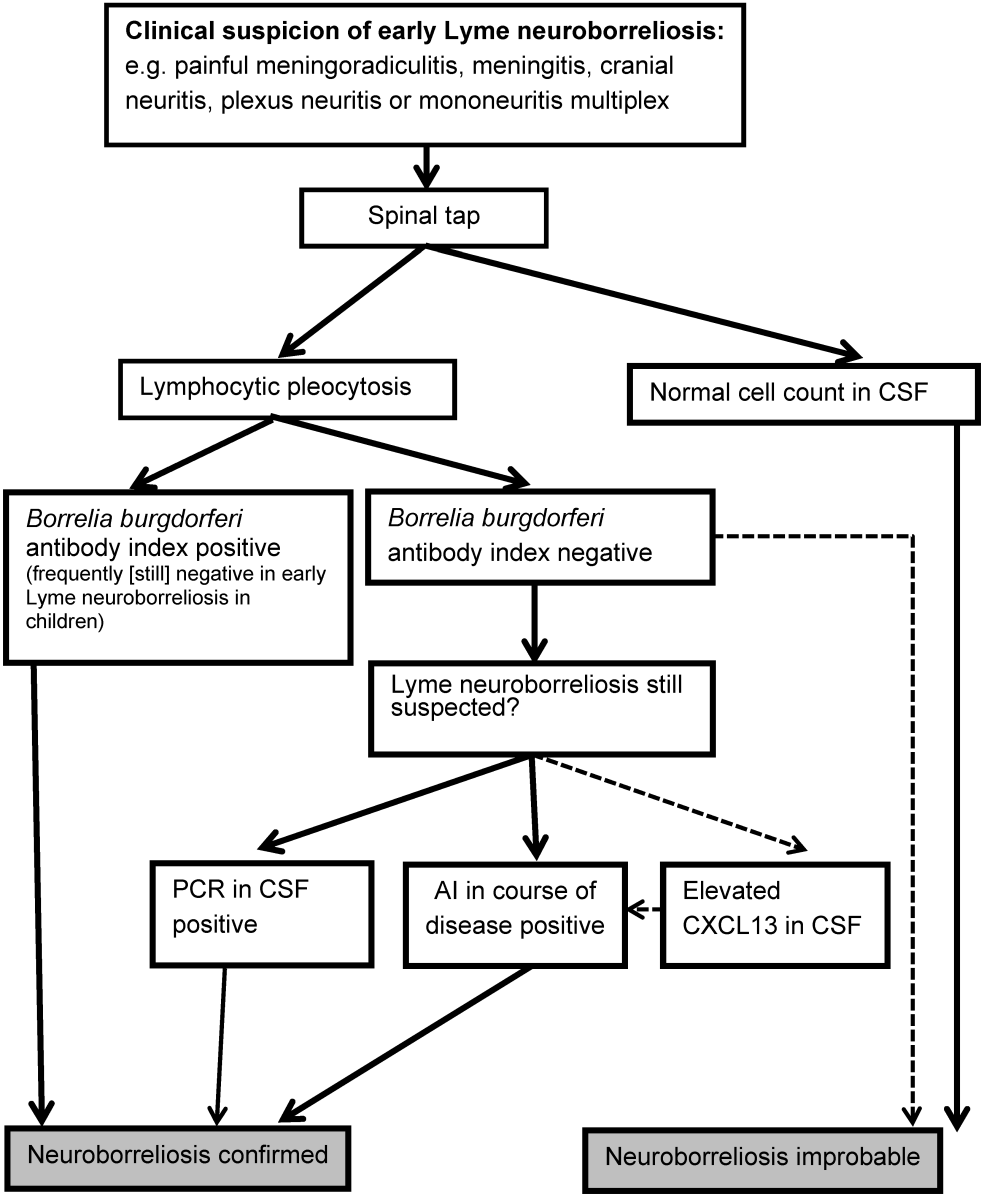
Diagnostic algorithm for early Lyme neuroborreliosis; modified according to Koedel [75]

**Figure 2:**
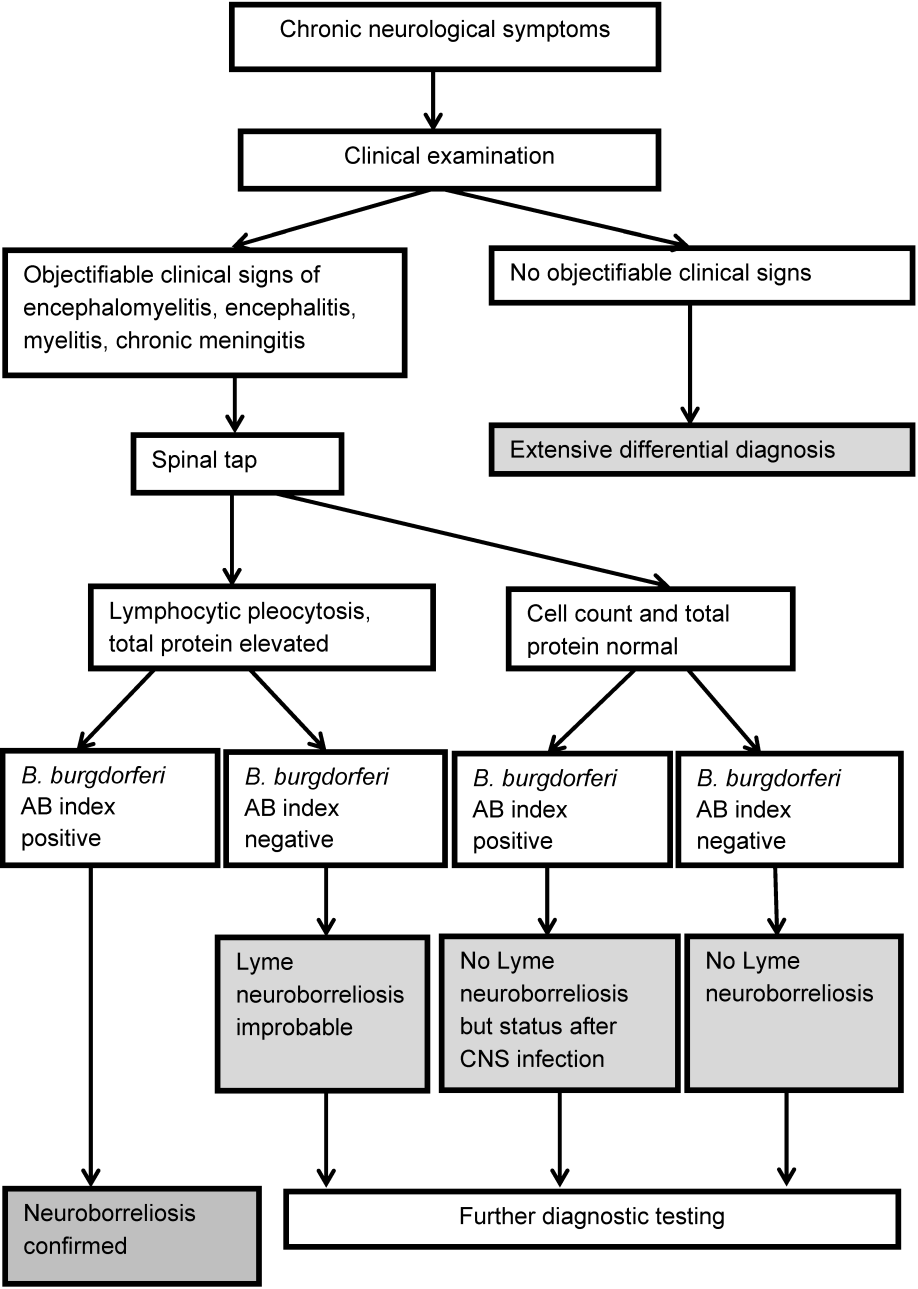
Diagnostic algorithm for late Lyme borreliosis; modified according to [75]

Lyme neuroborreliosis is indicated by typical clinical symptoms. These must be underpinned by subsequent laboratory tests (serum and cerebrospinal fluid tests) [11], [12]. The diagnostic algorithm is illustrated in Figure 1 (Fig. 1) and Figure 2 (Fig. 2).

### 3.2 Inflammatory CSF changes

Inflammatory CSF changes (pleocytosis, blood-cerebrospinal fluid barrier dysfunction and intrathecal immunoglobulin synthesis) can be expected in every case of Lyme neuroborreliosis (possible exceptions: very early stage of the disease or ACA-related polyneuropathy). 

The CSF typically shows a **lymphocytic pleocytosis** with plasma cells, activated lymphocytes and a significant **increase in the total protein** or albumin quotient (barrier dysfunction) [13], [14] (Table 1 (Tab. 1)). The average cell count is between 170 and 220/µl with a clear range from 6 cells/µl [14] to 1,100 cells/µl [15]. In addition, **intrathecal IgM synthesis** occurs in 80–100% of early manifestations and **IgG synthesis** in approx. 60% of patients [14], [106]. If the intrathecal IgG synthesis is qualitatively determined by isoelectric focusing (detection of oligoclonal IgG bands), results will be positive in 70–80% of patients [13], [14]. **Rates of intrathecal IgG and IgA** synthesis are higher and more frequent in late Lyme neuroborreliosis than in early Lyme neuroborreliosis (Table 1 (Tab. 1)). 

**CSF lactate levels** may be slightly elevated in individual patients with Lyme neuroborreliosis. Of the 118 patients with early Lyme neuroborreliosis, only 5 patients had significantly elevated CSF lactate levels (≥3.5 mmol/l) and the mean CSF lactate concentration across the entire cohort was not elevated (2.1±0.6 mmol/l) (Table 1 (Tab. 1)) [13].

**1. Recommendation (reviewed in 2023)**

*If Lyme neuroborreliosis is clinically suspected, CSF and serum testing (simultaneous collection) should be performed.*

EC ↑↑

Level of consensus: 100% (16/16)

**2. Recommendation (reviewed in 2023)**

*The CSF analysis should include cytology, protein chemical and serology tests (AI calculation, see below).*

EC ↑↑

Level of consensus: 100% (16/16)

### 3.3 Indirect pathogen detection in serum

#### 3.3.1 Serodiagnosis, antibody detection

In the case of early Lyme borreliosis, Borrelia-specific IgM antibodies can be detected starting in week 3 p.i. and IgG antibodies starting in week 6 p.i. [12]. However the use of VlsE and C6 peptide as test antigens means that IgG antibodies can now often be detected just as early as IgM antibodies [12]. High IgG antibody concentrations are usually found in late manifestations of Lyme borreliosis (Table 2 (Tab. 2)) [12], [107]. The course of the detectable humoral immune response often differs to that of other infectious diseases: as a result, a measurable antibody response may (still) be absent in an early, localised manifestation (erythema migrans) [12], or there may be no measurable IgM response, for example in the case of a reinfection [12], [108]. Antibiotic treatment very early on can also result in no measurable humoral immune response [109]. On the other hand, the positive detection of Borrelia-specific IgM and/or IgG antibodies alone is not an indication of a *Borrelia burgdorferi* infection, since

1. Borrelia infections can occur with asymptomatic seroconversion [48] and
2. healthy individuals often have elevated IgG and IgM antibody titres (in serum and/or CSF) for years after having received sufficient treatment for Lyme borreliosis [110], [111], [112].

Borrelia serology can therefore not be used as an acuteness parameter. It also follows that Borrelia serology is not suitable for monitoring the treatment of Lyme borreliosis with antibiotics and is therefore not recommended [12], [113].

The **serodiagnosis** of a systemic Borrelia infection is a 2-step process: a screening test (enzyme immunoassay) followed by a confirmation test (immunoblot) [12], [113]. The use of recombinant or purified native antigens represent improvements in the field of serodiagnosis. Thus, specific antigens can be selected, antigens not normally expressed in culture (in vitro) can be used, individual antigens from different genospecies can be combined, and cross-reactive epitopes can be eliminated. For example, recombinant forms of the highly sensitive protein VlsE, which is preferably only expressed *in vivo*, and the conserved immunodominant C6 region of this protein can be used [12], [114]. Of the confirmation tests (immunoblot) used to diagnose acute Lyme neuroborreliosis, the recombinant line immunoblot was reported to have a significantly higher sensitivity than the conventional immunoblot with a consistently high specificity (95%) [12], [115]. This was partly due to the new line immunoblot technique and partly to the widening of the antigen spectrum to include proteins only expressed by the Borrelia *in vivo* (in the host and not in culture).

#### 3.3.2 Diagnostically relevant Borrelia antigens

*Borrelia burgdorferi* has a large number of immunologically relevant antigens which, depending on the stage of disease, can be detected with varying degrees of sensitivity and which sometimes have a different specificity. They should be taken into account when interpreting serology test results (detailed description in MiQ Lyme borreliosis [12]).

**Early immune response** (particularly IgM) [115], [116], [117], [118]:

- Flagellar protein (Flagellin, p41 or internal recombinant fragment)
- OspC (associated with outer membrane)
- VlsE

**Late immune response** (particularly IgG) [116], [119], [120]:

- p83/100, p58, p43, p39, p30, p21, DbpA (Osp17) and p14 (on the whole reactive with around 80% of the sera [119])
- VlsE (detectable in more than 90% of the sera) [115]

**Non-specific antigens:**

- Flagellin
- Heat shock proteins

##### Summary

**7. Statement (reviewed in 2023)**

- *A positive antibody test is not evidence for a clinical case of Lyme borreliosis. *
- *A negative antibody test does not rule out an early manifestation of Lyme neuroborreliosis *
- *A negative antibody test largely rules out Lyme borreliosis in immune-healthy patients with a protracted case of the disease.*
- *An isolated positive result for IgM is an argument against a late manifestation of Lyme borreliosis *[12]*.*

EC

Level of consensus: 100% (16/16)

**3. Recommendation (reviewed in 2023)**

*Serology testing should only be ordered if there is sufficient clinical suspicion.*

*The testing should be done as a two-tier testing (screening test and confirmation test).*

[12]

Grade of recommendation ↑↑

Level of evidence: EC

Level of consensus: 100% (16/16)

### 3.4 Intrathecal antibody synthesis – Borrelia-specific antibody index (AI)

#### 3.4.1 Overview

For most patients with Lyme neuroborreliosis, the suspected clinical diagnosis can be confirmed by detecting Borrelia-specific intrathecal antibody synthesis associated with inflammatory CSF changes [12], [121], [122], [123]. The specific intrathecal antibody production is detected by determining the Borrelia-specific CSF/serum antibody index (Borrelia-specific AI) [24], [124], [125]. 

#### 3.4.2 Determination method

When **determining AI** as a dimensionless number, methods must be used that take into account the blood-cerebrospinal fluid barrier function, as this could otherwise produce false negative results [12]. The proven Reiber method should be used to determine the antibody index [12], [121], [126], [127]. The Borrelia-specific AI can be calculated using the following formula (here based on IgG, however it can also be used to calculate IgM and IgA):


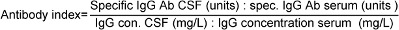


If the Reiber diagram shows intrathecal immunoglobulin synthesis, i.e. the total IgG ratio relative to the albumin ratio is above the limit (norm), the total IgG ratio must be replaced by the Q-Lim ratio (empirical limit value for the maximum IgG fraction derived from the serum as a function of the albumin ratio). In this case: 


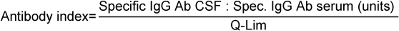


A value ≥1.5 is recommended as the **cut off** for a positive AI, unless assessed otherwise [12], [106], [126], [128]; the previously recommended higher limit value of 2.0 [129] is regarded as less sensitive, as long as a reliable test performance can be ensured [106]. When calculating the AI, quantitative measurement methods are usually used to determine the serological CSF and protein chemical parameters. These are calculated and interpreted using specific algorithms by means of validated commercial EDP-supported analysis systems [12]. 

It is important to note that there can be considerable fluctuations when calculating AI (both interrater-dependent using the same method and when comparing different methods) [106], which is why antibody testing and AI determination should be carried out by a designated specialist laboratory.

#### 3.4.3 AI throughout the course of the disease

Intrathecal *Borrelia burgdorferi*-specific antibody production develops in untreated patients from around week 2 and is detectable in over 99% of patients after 6–8 weeks [24], [121], [122], [123], [130]. During the course of the disease (acute disease), elevated CSF-Borrelia antibodies can sometimes be detected even though Borrelia antibodies are not detectable in serum [13], [130], [131]. Conversely, the borrelia-specific AI can (remain) inconspicuous in children with facial nerve paresis or when the duration of the disease is short [121], [130], [131]. Furthermore, very early antibiotic treatment can prevent the development of a measurable humoral immune response and cause the borrelia-specific AI to remain negative [132]. This is not an indication that treatment has been unsuccessful. 

After the Lyme neuroborreliosis has resolved, the Borrelia-specific AI can remain positive for months to years in symptom-free patients [14], [133], [134]. As a result, the borrelia-specific AI may only be interpreted in conjunction with clinical symptoms and other immunological and clinical chemistry parameters (e.g. protein, cell count, blood-CSF barrier dysfunction) and is not suitable per se for diagnosing an active case of Lyme neuroborreliosis or even as a way to determine treatment success [135].

##### Summary

**8. Statement (reviewed in 2023)**

- *The detection of borrelia-specific intrathecal antibody synthesis (positive borrelia-specific antibody index (AI)) in conjunction with inflammatory changes in the cerebrospinal fluid can confirm the suspected clinical diagnosis of Lyme neuroborreliosis.*
- *Borrelia-specific intrathecal antibody synthesis begins around week 2 of the disease and is detectable in over 99% of patients after 6–8 weeks. *
- *A Borrelia-specific AI without accompanying inflamma* *t* *o* *ry changes in CSF may remain positive for years after the Lyme neuroborreliosis has resolved and is therefore not suitable as a control parameter for antibiotic treatment and should not be interpreted as an indication of a current case of Lyme neuroborreliosis. *
  [12], [24], [121], [122], [123], [124], [125]

Level of evidence: EC

Level of consensus: 100% (15/15)

**4. Recommendation (reviewed in 2023)**

- *The Borrelia-specific AI should be determined (in conjunction with CSF-n) if Lyme neuroborreliosis is suspe* *c* *t* *ed. *
- *The Borrelia-specific AI should not be used to monitor treatment success. *
  [12], [24], [121], [122], [123], [124], [125]

Grade of recommendation ↑↑

Level of evidence: EC

Level of consensus: 100% (16/16)

### 3.5 Chemokine CXCL13

Recently it has been shown that chemokine CXCL13 levels increase significantly in the CSF of almost every patient with acute Lyme neuroborreliosis – even before a specific antibody response has been generated (if symptoms last <6 weeks, the Borrelia-specific AI can be negative in 10–30% of cases [136]). Once antibiotics are administered, chemokine levels drop very quickly, long before the CSF pleocytosis regresses [137], [138], [139]. A meta-analysis of pooled data from 18 studies found a diagnostic sensitivity of 89% (95% CI 85%–93%) and a specificity of 96% (95% CI 92%–98%) [140]. Results on a similar scale had previously been identified by a meta-analysis with pooled data from 7 studies [141], so that the parameter can be diagnostically useful in an inconclusive case of very early Lyme neuroborreliosis [12], [75]. 

It should be noted that the CXCL13 value is not specific to Lyme neuroborreliosis; elevated CSF values have also been found in neurosyphilis, tuberculous meningitis and CNS lymphomas [135], [137], [138], [139], [140], [142], [143], [144], [145]. Diagnostic testing currently lacks standardisation and internationally accepted cut-offs for the various assays. Therefore, the results of the various studies on this issue can only be compared to a very limited degree at the moment [135].

#### Summary

**9. Statement (reviewed in 2023)**

- *CXCL13 levels in cerebrospinal fluid correlate with “disease activity” (indication of an existing infection) of Lyme neuroborreliosis and can be diagnostically helpful in some cases of early Lyme neuroborreliosis. *
- *CXCL13 determination has not yet been standardised.*
- *Elevated CXCL13 values in CSF are not specific to Lyme neuroborreliosis.*
  [136], [137], [138], [139], [140]

Level Ia evidence

Level of consensus: 100% (15/15)

**5. Recommendation (reviewed in 2023)**

*CXCL13 can be determined in CSF when early Lyme neuroborreliosis is clinically suspected and the CSF cell count and/or Borrelia-specific AI are (still) inconspicuous.*

[136], [137], [138], [139], [140]

Grade of recommendation ↔

Level Ia evidence

Level of consensus: 100% (16/16)

### 3.6 Direct detection using molecular detection methods and culture

In certain cases (e.g. in immunocompromised patients (e.g. insufficient antibody production in patients with primary immunodeficiency or B-cell depletion)), Borrelia infections can be underpinned by pathogen detection in cerebrospinal fluid [12], [146]. However, in cases of acute Lyme neuroborreliosis, the sensitivity of pathogen detection by culture or PCR in CSF is only 10–30% [12], [107]. Pathogen detection is assumed to have a higher sensitivity when the duration of illness is short (patients often still seronegative) than when it is long. For example, 50% of patients with acute Lyme neuroborreliosis had positive PCR results compared to only 13% of patients with a prolonged illness [147]. Detection in CSF by PCR is generally preferred because results can be provided faster than for cultures. If the results are positive, a species diagnosis should be made by analysing the PCR products. Pathogen detection in blood is not recommended because this method is even less sensitive [12]. The specificity of PCR tests is highly dependent on the quality of the laboratory performing the test. Therefore, testing should be explicitly restricted to special, designated reference laboratories, especially as further molecular confirmation tests are required if the result is positive [12]. PCR results must always be interpreted in relation to the symptoms and serology results. For example, positive PCR test results in patients with a protracted disease and negative serology are highly likely to be false positives [12].

Attending physicians should ensure that the laboratories commissioned with performing the diagnostic tests do so in accordance with the current diagnostic standards (MIQ, RiliBäk), regularly participate in external quality control schemes (EQAs), and have valid certificates [135].

#### Recommendations for direct detection using molecular methods and culture

**6. Recommendation (reviewed in 2023)**

*Molecular detection and direct detection in culture using cerebrospinal fluid should only be employed for the differential diagnosis of ambiguous cases (e.g. insufficient antibody production in patients with a primary immunodeficiency or B-cell depletion). *(Grade of recommendation ↑)

*Molecular detection and the cultivation of *Borrelia burgdorferi* sensu lato in culture should only be carried out by specialist and reference laboratories. *(Grade of recommendation ↑)

*Molecular detection or direct detection in culture should not be used as a screening test if Lyme borreliosis is suspected.* (Grade of recommendation ↑↑)

*Lyme neuroborreliosis should not be ruled out even if the results of the molecular test or culture are negative for the pathogen.* (Grade of recommendation ↓↓)

*A positive molecular test or detection in culture should be confirmed by further molecular testing and the detected genospecies should be reported in the findings. *(Grade of recommendation ↑↑)

Guideline adaptation: MIQ 12: Lyme borreliosis, Quality Standards for the Microbiological Diagnosis of Infectious Diseases [12]

Level of evidence: EC

Level of consensus: 100% (16/16)

**7. Recommendation (reviewed in 2023)**

*If test results are positive for Borrelia DNA after guideline-compliant antibiotic treatment, and if there are no typical clinical manifestations (see 2.5), the patient should not receive another round of treatment.*

EC ↓↓

Level of consensus: 100% (17/17)

### 3.7 Routine laboratory testing parameters in blood

In routine laboratory testing, patients with Lyme neuroborreliosis are found to have normal or slightly elevated levels of ESR, CRP, leukocytes and transaminases. This indicates a systemic infection (see Table 3 (Tab. 3)). When testing for Lyme neuroborreliosis, routine laboratory testing only plays a role in the differential diagnosis. 

### 3.8 Diagnostic imaging – MRI

Due to the very rare involvement of the brain and spinal cord in early Lyme neuroborreliosis, the findings of magnetic resonance imaging (MRI) are usually unremarkable; in this case, an MRI is primarily used in making differential diagnoses. In contrast, an MRI that includes MR angiography is indispensable for diagnosing Borrelia-induced vasculitis; both cerebral ischaemia and intracranial vascular stenoses can be detected through MR imaging [15], [95], [96]. In addition, inflammatory lesions were detected by MRI in individual cases of encephalomyelitis manifestations [15], [75]. There are no controlled studies on the diagnostic value of MRIs for Lyme neuroborreliosis. 

### 3.9 Examination and testing

**8. Recommendation (reviewed in 2023)**

*The following examinations and tests should be condu* *c* *t* *ed if Lyme neuroborreliosis is clinically suspected (for symptoms see 2.5):*

- *Detailed medical history with questions about tick bites, time spent in endemic areas, early symptoms (erythema migrans, multiple erythema migrantia, Borrelial lymphocytoma (lymphadenosis cutis benigna), general symptoms), psychosocial history if necessary *
- *Neurological state, examination of the skin (erythema migrans may still be detectable when neurological symptoms appear)*
- *Basic lab tests with inflammation parameters *
- *CSF analysis: cell count, differential cell count, total protein, immunoglobulins, lactate *
- *Borrelia serology including Borrelia-specific CSF/serum antibody index (AI)*
- *Possible determination of CXCL13 in CSF if constellation is inconclusive *

Grade of recommendation ↑↑

Level of evidence: EC

Level of consensus: 100% (17/17)

### 3.10 Diagnostic criteria for Lyme neuroborreliosis

**10. Statement (reviewed in 2023)**

*Depending on the constellation of the clinical and laboratory findings, a Lyme borreliosis diagnosis can be classified as “possible”, “probable” or “confirmed” (see below) *[11]*, *[148]*. *

*Possible Lyme neuroborreliosis*

- *Typical clinical picture (cranial nerve deficits, meningi* *t* *i* *s/* *meningoradiculitis, focal nerve deficits; see 2.5)*
- Borrelia-specific IgG and/or IgM antibodies in serum*
- *CSF findings unavailable/spinal tap not performed*
- *Differentiated from other causes *

*The serology can [still] be negative in very early stages of the disease.

*Probable Lyme neuroborreliosis*

*As with “possible Lyme neuroborreliosis”, however also*

- *Inflammatory cerebrospinal fluid syndrome with lymphocytic pleocytosis, blood-CSF barrier dysfunction and intrathecal immunoglobulin synthesis *

*Confirmed Lyme borreliosis*

*As with “probable Lyme neuroborreliosis”, also*

- *Intrathecal synthesis of Borrelia-specific antibodies (positive IgG and/or IgM antibody index) in CSF or*
- *Positive culture or nucleic acid detection (PCR) in cerebrospinal fluid*

Level of evidence: EC

Level of consensus: 100% (17/17)

### 3.11 Testing methods not suitable for diagnosing Lyme neuroborreliosis

There are no prospective controlled studies for the methods listed below that would prove useful in diagnosing Lyme neuroborreliosis. The following negative recommendations have been taken from the Microbiological Guideline for the Diagnosis of Lyme Borreliosis [12].

**9. Recommendation (reviewed in 2023)**

*Antigen detection in bodily fluids* (Grade of recommendation ↓↓)

*PCR in serum and urin*e (Grade of recommendation ↓↓)

*Lymphocyte transformation tests (LTT) *[149]*, *[150]*, *[151]*, *[152]*, *[153]*, *[154] (Grade of recommendation ↓↓)

*Enzyme-linked immunospot assay (ELISPOT) *[153]*, *[154]*, *[155] (Grade of recommendation ↓↓)

*Visual contrast sensitivity test (VCS test or grey scale test): a lipophilic neurotoxin from Borrelia should be indirectly detected by measuring the recognition of grey shades *[156]*.* (Grade of recommendation ↓↓)

*Detection of so-called L forms or spheroplasts *[157] (Grade of recommendation ↓↓)

*Detection of immunocomplexes as markers of disease activity* (Grade of recommendation ↓↓)

*CD57 positive/CD3 negative lymphocyte subpopulation *[153]*, *[158] (Grade of recommendation ↓↓)

*Commercially available rapid serology tests (insufficient sensitivity (18–32%)) *[153]*, *[159] (Grade of recommendation ↓↓)

Guideline adaptation: MIQ 12: Lyme borreliosis, Quality Standards for the Microbiological Diagnosis of Infectious Diseases [12]

Level of evidence: EC

Level of consensus: 82% (14 yes, 3 no)

## 4 Chronic and atypical symptoms linked to Lyme neuroborreliosis

### 4.1 Introduction

In addition to the confirmed early and late manifestations of Lyme neuroborreliosis (such as radiculitis, meningitis or encephalomyelitis and/or the residual symptoms related to them), there is a broad range of persistent symptoms which are suspected to be causally linked to Lyme neuroborreliosis where no inflammatory or infectious process can be detected through laboratory testing on the basis of generally accepted criteria [75], [160], [161], [162], [163], [164]. The terms used for these chronic symptoms include “post-treatment Lyme disease syndrome” (PTLDS), “(post-)Lyme encephalopathy” or simply “chronic Lyme (neuro-) borreliosis” without a clear distinction made between what they entail. All three conditions are characterised by the fact that they are predominantly accompanied by general, non-specific symptoms. There is an ongoing debate as to whether additional courses of antibiotics are useful in such cases, even though no studies have provided reliable evidence for this [164], [165].

A systematic analysis was conducted to investigate the frequency and range of persistent symptoms following antibiotic treatment in patients who had had Lyme neuroborreliosis [27]. Of the 44 identified studies published between 1986 and 2014 (8 RCTs, 17 cohort studies, 2 case-control studies and 17 case series), 38 (n=1,469 patients) reported patients with residual symptoms. Overall, persistent or residual symptoms were identified in 28% of patients (95% CI 23–34%, n=34 studies). In studies where, according to the inclusion criteria (case definition), there was a “probable or definite” case of Lyme neuroborreliosis (inflammatory changes in CSF), the prevalence of persistent symptoms was significantly lower at 24% (95% CI 0.16–0.33; n=547) (p=0.0048) than in patients who only need to have a “possible” case of Lyme neuroborreliosis (CSF findings inconspicuous or unavailable) to be included in the study (31% (95% CI 0.25–0.37); n=922). In addition, the type of persistent symptoms also differed between the two patient groups. Non-specific symptoms, as typically reported for PTLDS (see 4.3), were statistically significantly more common in patients with “possible” Lyme borreliosis than in patients with “probable/definite” Lyme neuroborreliosis: fatigue (5.13% vs. 0%), cognitive disorders (16.67% vs. 1.6%), general pain (18.75% vs. 2.77%), headaches (8.33% vs. 1.75%) (see Table 4 (Tab. 4)). Even if a study bias or the presence of different stages of disease in the cohorts cannot be definitively ruled out, the authors conclude that the clear prevalence of persistent atypical symptoms after Lyme neuroborreliosis, as reported in the studies, is largely due to study artefacts resulting from unclear case definitions. 

An observational study, which retrospectively analysed 1,261 patients, also provides evidence that it is generally difficult to test for and diagnose Lyme borreliosis. All of study’s patients had presented with a suspected case of Lyme borreliosis at an outpatient clinic which specialised in infectious diseases at a US university [166]. The experts were unable to confirm the diagnosis of Lyme borreliosis in 911 (72.2%) of these patients, even though 764 (83.9%) of these patients with the unconfirmed diagnosis had already received antibiotic treatment. When the patients with unconfirmed Lyme borreliosis were compared to the patients with a confirmed diagnosis, the characteristics that were identified at a significantly higher rate were duration of illness (>3 months) and number of symptoms, the diagnosis of co-infections, gender (f>m) and the number of laboratory tests performed. 

### 4.2 Presumptive chronic Lyme neuroborreliosis

#### 4.2.1 Introduction

Confusingly, the terms “chronic Lyme borreliosis”’ and “chronic Lyme neuroborreliosis” are used in an overlapping sense and have very different meanings and correspondingly different therapeutic consequences. They mostly refer to non-specific symptoms such as fatigue, musculoskeletal pain, cognitive disorders and depression [163], [164], [165], [167], [168], [169], [170], [171], [172]. In terms of the pathophysiology of presumptive “chronic Lyme borreliosis” or “chronic Lyme neuroborreliosis”, current systematic reviews have found no scientific basis for the presumption of a persistent latent infection caused by *Borrelia burgdorferi* [163] or its morphological variants [157]. Likewise, no evidence has been found for chronic co-infections transmitted by tick bites in patients with non-specific symptoms [173]. Feder et al. assigned patients with presumptive “chronic Lyme borreliosis” to 4 clinical categories (see Appendix 1 in Attachment 2 for a complete list of the criteria according to Feder) [164].

- **Category 1 **includes patients with symptoms of an unknown origin without evidence of an infection with *Borrelia burgdorferi*.
- **Category 2** includes patients with symptoms of a known, well-defined illness without evidence of an infection with *Borrelia burgdorferi*. Here the original diagnosis is presumed to be false.
- **Category 3** describes patients with symptoms of an unknown origin, whose serology test was positive, but where there are no objective clinical findings of Lyme borreliosis.
- **Category 4** refers to patients with PTLDS-like symptoms (for PTLDS see section 4.3 and Appendix 2 in Attachment 2).

#### 4.2.2 Current study situation

Older studies, in which patients with presumed “chronic Lyme borreliosis” were re-evaluated at specialised academic centres, primarily featured category 1 and 2 illnesses according to Feder [174], [175], [176]. Later studies on this topic examined 240 US-American patients [177], 29 Norwegian patients [178], 95 German patients [179] and 200 Dutch patients [180]. In summary, Lyme borreliosis was confirmed in a smaller percentage of patients (13–24%). PTLDS was assumed in 6–20% of patients with no proven causal link to Lyme borreliosis and no indication for antibiotic treatment (see above). A diagnosis remained undetermined in 18–52% of cases. These studies suggest that an intensive differential diagnosis of both organic and psychosocial disease factors be carried out when “chronic Lyme borreliosis” is suspected [180], [181]. Further research is needed in light of the wide range of study results cited here.

#### 4.2.3 Practical approach

There is no rationale behind administering antibiotics to patients falling under categories 1 and 2 according to Feder. For category 4 patients, the current data does not indicate the need for antibiotic treatment (see section 4.3 on PTLDS). Probatory (oral) antibiotic treatment may be considered for patients with category 3 symptoms according to Feder [164]. However, these patients should be made aware that, in their situation, a diagnosis of Lyme borreliosis is very uncertain as the predictive value of borrelia serology is very low when symptoms are non-specific [182], [183] and the transient “treatment effects” can be caused by either the placebo effect [184] or by the anti-inflammatory side effects of antibiotics [185], [186], [187].

**11. Statement (reviewed in 2023)**

*None of the 4 categories according to Feder *[164]* corresponds to a disease entity.*

Level of evidence: EC

Level of consensus: 88% (15 yes, 2 abstentions)

**10. Recommendation (reviewed in 2023)**

*Patients in categories 1, 2 and 4 according to Feder should not be treated for Lyme neuroborreliosis with antibiotics; instead, a symptoms-based differential diagnosis should be carried out and treatment should be performed on the basis of the main symptoms.*

[164]

Grade of recommendation ↑↑

Level of evidence: EC

Level of consensus: 82% (14 yes, 3 abstentions)

**11. Recommendation (reviewed in 2023)**

*For category 3 patients according to Feder, a single course of antibiotics may be considered for 14–21 days in individual cases after a detailed differential diagnosis has been conducted and with indication of an unconfirmed diagnosis.*

[164]

Grade of recommendation ↔

Level of evidence: EC

Level of consensus: 88% (15 yes, 2 abstentions) 

### 4.3 Symptoms following treatment: post-treatment Lyme disease syndrome (PTLDS)

#### 4.3.1 Diagnostic criteria

Several studies have found that a certain percentage of Lyme borreliosis patients who have received guideline-compliant treatment continue to suffer from existing or newly occurring non-specific symptoms such as muscle and joint pain, paraesthesia, fatigue, as well as concentration and memory issues [188], [189], [190], [191].

Non-specific symptoms that last for more than 6 months are referred to by some authors as post-treatment Lyme disease syndrome (PTLDS) [53], [164], although it should be noted that this syndrome has yet to be universally defined. As a result, different definition criteria are sometimes used in studies, which makes classification more difficult. 

PTLDS-like symptoms after treatment occur both in patients with EM and in patients with disseminated disease such as Lyme neuroborreliosis. There are indications that non-specific symptoms occur more frequently after treatment in disseminated or late Lyme borreliosis than in patients with early manifestations such as EM [75].

Current studies do not present a uniform picture and some studies were unable to identify significant differences between certain symptoms when patients who received guideline-compliant treatment for Lyme Borreliosis were compared to the general population or control subjects [192], [193], [194], [195], [196], [197].

PTLDS must be distinguished from a confirmed late manifestation, objectifiable symptoms resulting from the persistence of reproduction-capable pathogens, and symptoms resulting from partial recovery. 

#### 4.3.2 Frequency

A non-systematic review found that 0–20% of patients treated for Lyme borreliosis with antibiotics had symptoms of so-called PTLDS; after treatment of Lyme neuroborreliosis the percentage was between 5 and 54% [75].

#### 4.3.3 Subjective symptoms in case-control studies 

The frequency of subjective symptoms was investigated in case-control studies that compared cohorts of patients who had had Lyme borreliosis with those who did not. Since PTLDS-like symptoms are non-specific and are common in the general population (Luo et al. [198]; Wessely [199]), it can be problematic to attribute them to Lyme neuroborreliosis in the sense of a causal secondary disease. The issue is also reflected in the very heterogeneous data: non-specific symptoms were not found at a higher frequency in German adults and Swedish and US children with long-term Lyme neuroborreliosis following treatment when compared to control subjects [193], [194], [192], [200]. The same was true for European patients after treatment of erythema migrans [197] and for American patients after various manifestations of Lyme borreliosis [195], [196]. In contrast, other case-control studies found a significant increase in non-specific symptoms in children and adults after treatment of Lyme neuroborreliosis [22], [172], [201] or after any form of manifestation of Lyme borreliosis [202], [203], [204]. A meta-analysis examined 5 of the studies cited above [195], [192], [201], [202], [203] and concluded that there was an overriding link between the chronic symptoms of PTLDS and a previous case of Lyme borreliosis [205]. This meta-analysis is criticised for taking into account various retrospective studies whose diagnostic criteria and antibiotic treatment no longer conform to current standards [206]. 

According to another study, fatigue and depression can lead to physical and psychological impairment in patients with PTLDS-like symptoms [207], which is why the authors recommend targeted treatment of these primary symptoms.

A large population-based prospective study from the Netherlands [208] analysed persistent symptoms following Lyme borreliosis. Patients with Lyme borreliosis (n=1,135) and patients who were bitten by a tick but did not develop Lyme borreliosis (n=2,405) were recruited using an online tool (https://www.tekenradar.nl). An additional comparison group comprised people who were randomly selected from the general population and who matched the Lyme borreliosis group in terms of age, gender, region and month of event (n=4,000). Patients and subjects filled in questionnaires on fatigue, cognition, quality of life and pain at the time of enrolment in the study and then every 3 months for a total of 12 months.

The primary endpoint was a higher rate of residual symptoms in patients with Lyme borreliosis (27.2%) compared to patients who were bitten by ticks but did not develop Lyme borreliosis (23.3%) and the general population (21.2%). 

In terms of bias, this study has an overall critical risk of bias according to the Cochrane Collaboration’s ROBIN-I tool for non-randomised studies. A methodologically critical point, as already noted by Dessau et al. [209], is the non-controllable confounder of patient self-inclusion (volunteer bias). As there was no prospective inclusion of patients, a bias towards patients with a higher risk of residual symptoms can be assumed here. In addition, there are concerns due to a relevant amount of missing data in the course of the study (up to >50%). Even though the sensitivity analyses show robust results, there is a lack of best and worst-case scenarios. Given the high level of missing data, a serious risk of bias can also be assumed. 

Fallon et al. [210] used data from national patient databases in Denmark to analyse the rate of psychiatric illnesses and suicidality following Lyme borreliosis. Here, data on the treatment of Lyme borreliosis in Danish hospitals were combined with psychiatric diagnoses and suicide attempts reported during the course of the respective patients’ illness. The remaining individuals included in the study were used as a control group, adjusting for age, gender, time of year, marital status, education, socioeconomic status and comorbidities. A total of 12,616 people were identified who were diagnosed with Lyme borreliosis in a hospital. 

Patients with Lyme borreliosis were found to have a 28% higher rate of psychiatric disorders. In addition, there was also a higher rate of affective disorders, suicide attempts and suicides in the group who were diagnosed with Lyme borreliosis in a hospital compared to the rest of the population. In contrast, a subgroup of patients classified as having Lyme neuroborreliosis showed no difference to the comparison cohort with regard to psychiatric illnesses, affective disorders or suicides. 

In terms of bias, this study has an overall critical risk of bias according to the Cochrane Collaboration’s ROBIN-I tool for non-randomised studies. A major critical point is the non-controllable confounder that treatment data from hospitals is used, as it can be assumed that a large proportion of patients with Lyme borreliosis, especially with the most common manifestation of erythema migrans, are not diagnosed and treated in hospitals. Thus, the external validity of the analysed cohort is limited. The subgroup of patients with Lyme neuroborreliosis did not show an increased rate of psychiatric illness and suicidality during the course of the disease, although a greater initial burden of disease can be assumed here compared to patients with erythema migrans. In addition, the rate of hospitalisations due to fractures in patients with Lyme borreliosis and in the other patients included in the study was investigated as an independent “negative control”. This showed an increased rate of fractures in the Lyme borreliosis group. The rate of fractures is not dependent on the psychiatric endpoints and is therefore indicative of a residual confounder in the composition of the group. The aforementioned objections thus indicate a non-representative composition of the patients included in the Lyme borreliosis group. 

Due to the critical risk of bias, these two studies [208], [210] are considered too methodologically problematic to provide valid evidence. 

#### 4.3.4 Neuropsychological symptoms in case-control studies

Current studies are contradictory with regard to the frequency of neuropsychological symptoms. In addition to subjective symptoms, objective neuropsychological impairments (verbal and visual memory, attention, executive functions) ≥30 months after treatment for neuroborreliosis have also been described as a possible consequence of the disease [211], [212]. However, this has not been confirmed by any other study [200] nor has it been confirmed in children who previously had Lyme neuroborreliosis (facial paresis) [201]. In addition, further studies showed – at least in subgroups – impaired memory performance, primarily with verbal tasks, compared to healthy controls or patients who had fully recovered [202], [213], [214], [215], [216], [217], [218]. However, these studies also had contradictory results [196], [203], [219], [220]. 

#### 4.3.5 Treatment studies on post-treatment Lyme disease syndrome (PTLDS)

A systematic literature search using a predefined search strategy was conducted on 31 July 2023 to find relevant primary studies on the treatment of PTLDS in the period up to July 2023. The literature search yielded a total of 1,274 studies. After screening the abstracts (RD), a total of 48 studies were identified for a full-text screening. Of these, 9 entries were selected for qualitative analysis. The entries include a total of 8 randomised controlled trials (RCTs) [218], [220], [221], [222], [223], [224], [225], [226]. Details of the studies can be found in Tab. 14 in Attachment 2, Appendix 10. Overall, there is a heterogenous picture of various interventions and clear differences in the patient populations included in the studies. 

##### Evaluation of the evidence

To assess the quality of the evidence, the risk of bias was first assessed for each individual study. Then two reviewers (RD, GT) independently rated the available body of evidence for individual endpoints using the GRADE approach. 

Two RCTs had a high risk of bias, so data from these studies were not used in further assessments [224], [226].

One RCT had an overall low risk of bias [221]. In the other RCTs, the risk of bias in various domains was determined to be “unclear” as a result of incomplete reporting. The other RCTs were rated as having an unclear risk of bias for some aspects due to incomplete reporting. The risk of bias assessment is shown in Fig. 9 of the Guideline Report in Attachment 1. 

Relevant endpoints for the analysis were quality of life, fatigue, depression and cognition. A quantitative evidence synthesis was not performed due to significant differences in the inclusion criteria, the interventions analysed, the duration of treatment, the measurement, and the time at which the individual endpoints were measured. The results of the included studies are therefore qualitatively and narratively summarised below. 

##### Narrative synthesis

**Quality of life**

Four RCTs with a total of 457 participants compared treatment with antibiotics to treatment with a placebo in patients with PTLDS [218], [221], [222], [223]. Quality of life was analysed in all RCTs using an SF-36 questionnaire. The antibiotics that were analysed were doxycycline, ceftriaxone and clarithromycin/hydroxychloroquine. The duration of treatment ranged from 3 to 12 weeks. 

All 4 RCTs found that, in terms of quality of life, there was no difference between treatment with antibiotics and treatment with a placebo in patients with PTLDS. 

Using the GRADE approach, the quality of the body of evidence available for this endpoint was rated “low” (Appendix 10 in Attachment 2, Tab. 15). 

**Fatigue**

Three RCTs with a total of 360 participants compared, with regard to fatigue, treatment with antibiotics to treatment with a placebo in patients with PTLDS [218], [221], [222]. Various instruments were used to assess fatigue. The antibiotics that were analysed were doxycycline, ceftriaxone and clarithromycin/hydroxychloroquine. The duration of treatment ranged from 4 to 12 weeks. 

Two RCTs with 317 participants, one of which had an overall low risk of bias, found that, with regard to fatigue, there was no difference between treatment with antibiotics and treatment with a placebo [218], [221]. One RCT with 48 participants with a somewhat unclear risk of bias reported a lower rate of fatigue in the patient group treated with antibiotics [222].

Using the GRADE approach, the quality of the body of evidence available for this endpoint was rated “very low” (Appendix 10 in Attachment 2, Tab. 15). 

**Depression**

Two RCTs with a total of 161 participants compared, with regard to depression, treatment with antibiotics to treatment with a placebo in patients with PTLDS [218], [220]. The BDI questionnaire was used in both studies to analyse depression. The antibiotics investigated were doxycycline and ceftriaxone. The duration of treatment ranged from 70 to 90 days. 

At the end of the observation period, both RCTs found that, in patients with PTLDS, there was no difference with regard to depression between treatment with antibiotics and treatment with a placebo. 

Using the GRADE approach, the quality of the body of evidence available for this endpoint was rated “low” (Appendix 10 in Attachment 2, Tab. 15). 

**Cognition**

Four RCTs with a total of 489 participants compared, with regard to cognition, treatment with antibiotics to treatment with a placebo in patients with PTLDS [218], [220], [221], [222]. A variety of different instruments were used to analyse cognition in the respective studies. 

The antibiotics analysed were doxycycline, ceftriaxone and clarithromycin/hydroxychloroquine. The duration of treatment ranged from 4 to 12 weeks. 

At the end of the observation period, all of the 4 RCTs found that, in patients with PTLDS, there was no difference with regard to cognition between treatment with antibiotics and treatment with a placebo. 

Using the GRADE approach, the quality of the body of evidence available for this endpoint was rated “low” (Appendix 10 in Attachment 2, Tab. 15). 

##### Summary

In summary, the analysed RCTs found that there was no significant difference between the verum and placebo groups with regard to the endpoints quality of life, depression and cognition. Thus, even though the body of evidence was classified as “low”, there is Level Ib evidence regarding the ineffectiveness of antibiotics when it comes to treating the symptoms of PTLDS on the basis of these studies. 

With regard to fatigue, a study with 48 participants showed a lower rate of fatigue in the patient group treated with antibiotics [222]. Critics contend that 1) the effect is very marginal (score improvement in FSS-11: 22% versus 9% verum/placebo (p<0,01)); 2) patients in the verum group still had very severe fatigue after treatment (mean FSS-11=4.4), meaning that they still met the inclusion criteria of the study; 3) the result of the 2^nd^ fatigue scale (Fatigue-VAS) was not significant and 4) the patients themselves did not perceive an improvement based on a health-related quality of life scale (first question of SF-36) [223]. In light of the very small effects and the fact that the study recorded a critically high number of protocol drop-outs (33% of placebo patients) [224], the validity of this study is called into question from a methodological point of view [223]. In addition, this study is in contrast to 2 RCTs with 317 participants, one of which was a methodologically high-quality study with a low risk of bias (Berende), which showed no difference between treatment with antibiotics and treatment with a placebo in relation to fatigue [218], [221]. Thus, with regard to fatigue, there is level Ib evidence for the ineffectiveness of antibiotics in treating PTLDS. 

A follow-up analysis of the PLEASE study reveals the complexity of possible influencing factors in controlled studies in connection with non-specific, long-term symptoms. The PLEASE study originally investigated the effects of different antibiotic regimens in patients with non-specific long-term symptoms associated with Lyme borreliosis. The study yielded a negative result with regard to the defined endpoints [221]. The follow-up study showed that the study subjects’ positive expectations of an improvement in symptoms as a result of the antibiotics correlated significantly with a better result in terms of health-related mental and physical quality of life. The authors conclude that the expectations of patients in treatment studies can influence the outcome of the study and should therefore be taken into account. They also note that, in the clinical setting, optimised information about the treatment prospects before starting antibiotic treatment could have a positive influence on treatment outcomes [227].

#### 4.3.6 Pathophysiology

The pathophysiology of so-called PTLDS is unclear and an autoimmune process has not been proven [164], [228], [229]. In light of the negative or marginal effects of repeated courses of antibiotic treatment (see Treatment, section 4.3.5), a chronic infection is unlikely. This assumption is further supported by the following aspects [164] no accompanying objective clinical signs of the disease and/or inflammation with progression [225], [230], persistence of symptoms irrespective of a positive Borrelia serology [225], [230], [231], no pathogen detection by culture and/or PCR [225], [232], no proven resistance of *Borrelia burgdorferi* sensu lato to the commonly administered antibiotics [161], [233].

**12. Statement (reviewed in 2023)**

*Due to inconsistent data, so-called PTLDS cannot be defined as a disease entity.*

*There are no controlled studies on the frequency of so-called PTLDS.*

*The data refute the assumption of a chronic infection with *Borrelia burgdorferi* in patients with symptoms of so-called PTLDS.*

Level of evidence: EC

Level of consensus: 82% (14 yes, 3 no) 

**12. Recommendation (reviewed in 2023)**

*Symptom-based differential diagnosis and treatment should be carried out when there are PTLDS-like symptoms. This also includes an orientating psychosocial patient history and an assessment of findings. A causal treatment for PTLDS is not known as it cannot be defined as a disease entity.*

Grade of recommendation ↑↑

Level of evidence: EC

Level of consensus: 71% (12 yes, 5 no)

**13. Recommendation (reviewed in 2023)**

*If so-called PTLDS is identified, no antibiotics should be given.*

Grade of recommendation ↓↓

Level Ib evidence

Level of consensus: 94% (16 yes, 1 no)

**Further guidelines exist for PTLDS-like symptoms:**

- DEGAM S3 guideline on fatigue, AWMF Register No. 053/002 [234]
- DIVS S3 guideline on fibromyalgia syndrome, WMF Register No. 041/004 [235]
- DEGAM S1 guideline on chronic pain, AWMF Register No. 053/036 [236]
- National Disease Management Guideline (S3) “Unipolar Depression”, AWMF Register No. nvl-005 [237]
- DGN guideline (S2e) on the diagnosis and treatment of memory disorders, AWMF Register No. 030/124 [238]
- DGPM/DKPM S3 guideline “Management of patients with non-specific, functional and somatoform physical complaints”, Register No. 051/001 [239]
- Update of the Swiss guidelines on post-treatment Lyme disease syndrome [240]

### 4.4 Lyme encephalopathy

The term “Lyme encephalopathy” was originally coined in the 1980s, when several clinical manifestations of Lyme borreliosis were first described. At the time, patients frequently suffered from an undiagnosed, detectably active Borrelia infection (e.g. arthritis or ACA) for months or even years, reporting cognitive symptoms including memory disorders, which usually regressed after antibiotic treatment [214], [241], [242], [243]. In these case series, encephalitis was identified in only a small number of patients who exhibited focal neurological deficits, corresponding changes in their cerebrospinal fluid and/or abnormalities in the imaging [242]. The majority of these patients experienced “toxic-metabolic” encephalopathy as found in systemic (non-neurological) infections or inflammatory diseases (sepsis, pneumonia, urinary tract infections, active rheumatoid arthritis etc.) [229], [244], [245]. As this is a non-specific reaction of the brain to a systemic inflammatory process, the term “Lyme encephalopathy” should only be used in connection with the historical publications cited above. 

Other authors use the term Lyme encephalopathy in connection with cognitive complaints in patients with PTLDS [207], [218]. Since it is not possible to distinguish the term “Lyme encephalopathy” from its more historical use in the 1980s, this term should currently not be used as a diagnosis or syndrome designation. 

**14. Recommendation (reviewed in 2023)**

*The term Lyme encephalopathy should not be used in diagnoses due to its unclear definition and contradictory use.*

Grade of recommendation ↓↓

Level of evidence: EC 

Level of consensus: 100% (16/16)

## 5 Treating Lyme neuroborreliosis

### 5.1 Introduction

According to a systematic review [16], [246], there is limited evidence for treating Lyme neuroborreliosis with medication. After screening 5,779 reports in three databases, 8 randomised controlled trials (RCTs) and 8 non-randomised studies (NRS) could be included in the evaluation. The authors state that the conclusions for clinical practice need to be weighed against the fact that there is only a small number of studies with, at times, small cohorts, and that there is a relevant risk of diverse study bias (Appendix 8 in Attachment 2) [16]. 

A literature search on antibiotic treatment for Lyme neuroborreliosis was conducted in January 2023 as part of the updating of this guideline. After screening 1,530 database entries, 7 new publications on treating Lyme neuroborreliosis with antibiotics were identified (Appendix 3, Tab. 6 of this guideline in Attachment 2, and Fig. 1–5, Tab. 2–4 in the Guideline Report in Attachment 1 ). Two of the studies were randomised controlled trials (RCTs) [1], [17], one was a prospective cohort study [5] and four were retrospective cohort studies [3], [247], [248], [249]. All of the studies were assessed for risk of bias using the Cochrane Risk of Bias Tool (RCTs) [250] and/or ROBINS-I [251] (cohort studies). One RCT has a low risk of bias in all domains [1], the other RCT has a high risk of bias in relation to blinding. Both of the RCTs were included in the respective quantitative meta-analyses. The cohort studies all have a high, i.e. critical, overall risk of bias and are considered or discussed in the relevant sections of the guideline [3], [5], [247], [248], [249]. 

Only 3 studies included patients who did not receive antibiotic treatment [252], [253], [254]. In 2 of the studies, these patients were compared with patients who received antibiotic treatment [253], [254]. The studies are methodologically heterogeneous and yield low-precision, contradictory results. Therefore, a meta-analysis of this data is not useful (Appendix 8 in Attachment 2) [16]. Despite this, when weighing up the risks and benefits, there is no doubt that antibiotic treatment is indicated, especially as it can accelerate the regression of symptoms and counteract the development of late manifestations [20], [23], [24], [200], [255]. 

### 5.2 Early Lyme neuroborreliosis

#### 5.2.1 Duration of treatment

Eight RCTs and eight prospective cohort studies predominantly examined patients with early Lyme neuroborreliosis. The duration of antibiotic treatment in the RCTs was 14–21 days (with one exception of 100 days [2]). Treatment duration in the NRSs varied between 10 and 30 days, if it was indicated at all. The treatment effect on the primary endpoint (residual neurological symptoms) varied considerably in the 8 RCTs (10–66%) and in the 2 prospective cohort studies (7–44%) (Appendix 3 in Attachment 2, Tab. 6). Non-standardised survey methods were the main reasons for this broad range of results (neurological status, score system, patient self-assessment) as well as different assessment timeframes, including a wide range of assessment times within the individual studies themselves (3 RCTs: 3–12 months; 3 RCTs 12 months; 2 RCTS >3 months) [16]. 

When comparing different treatment durations, no differences in clinical outcomes were found between a 14-day and a 6-week administration of doxycycline [1] (RCT without evidence of relevant bias using the GRADE approach, see Fig. 2 in the Guideline Report in Attachment 1 ). Furthermore, there is indirect evidence from a prospective controlled study of 152 patients with disseminated Lyme borreliosis (80% with predominantly early neuroborreliosis (43% confirmed, 37% possible)) [2]. Here patients were initially treated with 2 g of ceftriaxone i.v. per day for 3 weeks. This was followed by randomised treatment for 100 days with either 1 g of amoxicillin p.o. per day or a placebo. After 1 year, around 90% of patients in both groups exhibited excellent or very good results. This study provides evidence that there is no benefit in extending treatment past 3 weeks (Level Ib). A pooled evaluation of both studies as part of a meta-analysis found there was no clinical advantage in the group that received antibiotics beyond a period of 14 to 21 days. Because there is a lack of evidence for the efficacy of longer treatment durations and there is level 1a evidence that treatment lasting 14 to 21 days is not inferior, there is no scientific basis for deviating from the previously recommended treatment duration of 14 days [129], [256] for early Lyme neuroborreliosis.

#### 5.2.2 Choice of antibiotics and side effects

Controlled clinical trials have evaluated beta-lactam antibiotics (penicillin G, ceftriaxone and cefotaxime) and doxycycline for treating Lyme neuroborreliosis as they penetrate well into cerebrospinal fluid. According to a meta-analysis, oral doxycycline and intravenous beta-lactam antibiotics show no statistically significant difference in the regression of neurological symptoms after a study period of 4–12 months (RR 1.27, 95% CI 0.98–1.63, P=0.07) or after more than 12 months (RR 0.98, 95% CI 0.68–1.42, P=0.93) and are therefore deemed to be equally effective (Level Ia) [16]. After incorporating the recently published RCT by Kortela et al. [17] into this meta-analysis, the results presented above were confirmed with regard to residual symptoms after more than 12 months (RR 0.98, 95% CI 0.76–1.26) (see Guideline Report, Fig. 3, in Attachment 1). These results also confirm an earlier meta-analysis by American authors [18]. 

Secondary endpoints, such as quality of life and fatigue, were examined in a follow-up study of one RCT [19], [22]. After 30 months, there was no significant difference between the patients treated with beta-lactam antibiotics and those who had received doxycycline (Level Ib). Two RCTs have shown that there are also no differences between these two antibiotic treatment regimens when it comes to the regression of CSF pleocytosis [16], [19], [20] (Level Ib). Two RCTS [2], [21] showed there was also no statistically significant difference in terms of the reported side effects (RR 0.82, 95% CI 0.54–1.25, P=0.35) (Level Ia). A meta-analysis updated to include the RCT by Kortela [17] in addition to the two older RCTs [19], [20] also showed that there were no differences between doxycycline and beta-lactam antibiotics with regard to side effects (Level Ia) (RR 0.94, 95% CI 0.63–1.39) (see Guideline Report, Fig. 3, in Attachment 1). The following side effects were reported: diarrhoea, nausea, constipation, reddening of the skin, dizziness and thrombophlebitis. Serious side effects such as cholecystitis, stomatitis, allergic reactions and duodenal ulcers were not reported enough in the studies to be able to make valid comparisons (Tables in Appendices 4 and 5 in Attachment 2) [16]. 

No studies have been conducted on higher doses of doxycycline than 200 mg/d, which is why no statement can be made in this regard [16]. 

Two RCTs compared cefotaxime and penicillin [257], [258] and found that cefotaxime had a significant advantage in terms of a lower rate of residual neurological symptoms after 4–12 months (RR 1.81, 95% CI 1.10–2.97, P=0.02). In contrast, patients who were treated with penicillin had a significantly lower rate of side effects (RR 0.54, 95% CI 0.35–0.83, P=0.005). The most frequent side effects (41%) were mild diarrhoea and Herxheimer-like reactions (Tables in Appendices 4 and 5 in Attachment 2). Since serious side effects such as colitis, shock and allergic reactions (3%) were not reported enough to make a comparative analysis [258], and since both studies are also subject to a significant risk of bias (Appendix 8 in Attachment 2), no recommendation can be derived from this data with regard to a preference of one of the substances over the other [16]. 

No valid, analysable study data are available on the efficacy of combination antibiotic treatment and no study data are available on the efficacy of chloroquine, carbapenems and metronidazole [16].

#### 5.2.3 Course of disease following antibiotic treatment

Most studies report a significant improvement in neurological symptoms several weeks to a few months after a 10-to-14-day course of antibiotic treatment. In a prospective study of 77 patients with Bannwarth syndrome, 88% of patients had good results 12 months after antibiotic treatment (Level IIa) [259]. The reported frequency of residual neurological symptoms is consistent with previous cohort studies in which 78/86 (90.6%) of the patients were symptom-free 3 months after antibiotic treatment [23] and 178/187 of the patients had very good results after 4–72 months (median 33) [24]. Another cohort study found that the daily activities of 100/114 (88%) patients with predominantly early Lyme neuroborreliosis were not impaired after an observation period of 5 years [191]. A systematic review examined residual symptoms in 687 patients with Lyme neuroborreliosis confirmed through CSF testing (probable/confirmed Lyme neuroborreliosis) [27]. It found the following rates of residual neurological symptoms after antibiotic treatment: sensory disorders 5.24%; cranial nerve paresis 3.6%; extremity paresis 2.33%, pain 2.77%; unsteady gait/dizziness/ataxia 2.62% (Appendix 6 in Attachment 2).

In a Scandinavian study, patients with encephalitic manifestations of Lyme neuroborreliosis were examined as part of a systematic review (n=45) and as part of a retrospective cohort study (n=35) [26]. The data from this study are of particular interest since encephalitis in the context of Lyme neuroborreliosis is rarely or never seen in the controlled treatment studies and, in many cases, corresponds to the course of late Lyme neuroborreliosis. The patients in the retrospective cohort study (n=35) were treated with doxycycline p.o. (n=15), ceftriaxone i.v. (n=4), penicillin i.v. (n=3) or a combination of these antibiotics (n=13) over a median period of 14 days (interquartile range 10–21 days). Most patients responded to antibiotic treatment within one week (n=17) or one month (n=16). At a follow-up examination after (a median) 298 days (interquartile range 113–389 days), 65.6% of patients had residual symptoms. Relevant restrictions in daily activities were reported in 35.5% of patients (modified Rankin Score >2). Overall, the rapid response to treatment and the stable neurological symptoms following antibiotic treatment indicate that antibiotic treatment over a period of 14 to 21 days is generally sufficient even in cases of Lyme neuroborreliosis where there are central symptoms such as encephalitis. In addition, the data suggest that oral doxycycline treatment is also effective in treating Lyme neuroborreliosis with central involvement. 

#### 5.2.4 Combination treatment with antibiotics and steroids

One prospective cohort study [5] and two retrospective cohort studies [3], [4] investigated the influence of administering steroids in addition to antibiotics on the clinical outcome of facial nerve paresis in patients with Lyme neuroborreliosis. All three studies have a critical risk of confounding and overall bias; the two retrospective studies have a critical risk of bias for classifying the interventions [3], [4], and two studies have a critical risk of bias with regard to measuring clinical outcomes [4], [5]. In one study, steroid administration was associated with a poorer outcome for facial nerve paresis after 12 months compared to only administering antibiotics [3]. In two studies, there was no statistically significant difference between the two study groups [4], [5]. Despite methodological limitations, it can be concluded from these studies that there is no evidence that additional steroid administration benefits patients with facial paresis in the context of Lyme neuroborreliosis, with possible indications that this could even be harmful. However, as steroid administration is the standard treatment for idiopathic facial paresis (see the DGN guideline on the treatment of idiopathic facial paresis), a diagnosis of Lyme neuroborreliosis should, in this case, be underpinned or confirmed through CSF results that indicate a “probable” or “confirmed” case of Lyme neuroborreliosis (see section 3.10). 

**15. Recommendation (as of 2023)**

*Administering steroids in addition to antibiotics to treat facial paresis in the context of a probable or confirmed case of Lyme neuroborreliosis in accordance with* *the diagnostic* *criteria (see section 3.10) is not r* *ec* *om* *me* *n* *d* *ed.*

[3]*, *[4]*, *[5]

Grade of recommendation ↓↓

Level III evidence

Level of consensus: 100% (17/17)

### 5.3 Late Lyme neuroborreliosis

There are no controlled studies that explicitly investigate treating late manifestations of Lyme neuroborreliosis (myelitis, encephalitis, encephalomyelitis) with antibiotics. In the 16 systematically analysed treatment studies (RCTs and cohort studies) [16] only 15 patients reportedly had late Lyme neuroborreliosis (Appendix 3 in Attachment 2). A separate evaluation of this form of manifestation is not possible due to a lack of data in the primary studies. However, residual neurological symptoms appear to occur more frequently than with early Lyme neuroborreliosis (Level III). In a case series that included 15 patients, only 3 patients (20%) were completely symptom-free 6 months after antibiotic treatment; however, serious symptoms, such as paresis, ataxia and bladder dysfunction, had completely regressed in 10/15 patients (66%) [23]. In a further cohort study, 8/8 patients with encephalomyelitis caused by late Lyme neuroborreliosis experienced neurological residuals after 4–72 months (median 33), with 5/8 (62%) having severely disabling residual symptoms [24].

The controlled studies and cohort studies [16] as well as the larger case series [23], [24] have shown no evidence of treatment failure when beta-lactam antibiotics or doxycycline are administered for 2 to 3 weeks (Level III). In addition, there are no studies that show that antibiotic treatment for more than 3 weeks produces any additional benefits. Therefore, there is no scientific basis for deviating from the previous recommendation of administering antibiotics for 2 to 3 weeks to patients with late manifestations. 

Moreover, doxycycline has also been shown to be equally effective in reducing CSF pleocytosis in 26 patients with Lyme encephalitis and/or myelitis compared to 115 patients with a radicular manifestation (Bannwarth syndrome) (Level Ib) [25]. Based on the data, doxycycline is assumed to be effective regardless of the severity of the symptoms of Lyme neuroborreliosis – as the authors conclude – however this has not been proven. Please refer to section 5.2.3 for cohort data on the duration of antibiotic treatment and the efficacy of doxycycline in patients with encephalitic manifestations of Lyme neuroborreliosis. 

Polyneuritis associated with ACA improves clinically – albeit slowly – after antibiotic treatment, while electrophysiological abnormalities do not change significantly after a mean follow-up period of 18.5 months (range 11–50 months) [260]. The authors regard this finding as being a partial recovery rather than an indication of a persistent infection. 

### 5.4 Cerebral vasculitis resulting from Lyme borreliosis

There are no controlled studies on the treatment of – very rare – cerebral vasculitis resulting from Lyme borreliosis. Case reports, case series and narrative reviews have reported that early antibiotic treatment with ceftriaxone and/or doxycycline has very good outcomes [25], [95], [96], [261], [262], [263], [264] (Level IV). Several authors administered steroids in addition to antibiotics [105], [263], [265], [266] (Level IV). In 2 case reports, clinical stabilisation was not achieved despite antibiotic and steroid administration until after the patient received subsequent immunosuppressive cyclophosphamide therapy (Level IV); two cases with basilar artery involvement were lethal [267], [268]. In summary, in the case of cerebral vasculitis due to Lyme borreliosis, antibiotic treatment should begin as soon as possible; it remains unclear whether the additional administration of steroids and/or prophylactic platelet function inhibition with 100 mg of ASA, in line with the recommendations for autoimmune mediated cerebral vasculitis, is beneficial (DGN S1 guideline on cerebral vasculitis, AWMF Register No. 030-085 [269]). 

### 5.5 Treating Lyme neuroborreliosis in children

According to a systematic review [270], the scientific data on administering antibiotics to treat Lyme neuroborreliosis in children is very limited and existing studies are of poor quality. Two RCTs and four NRSs (one prospective and three retrospective cohort studies) were identified as being analysable studies. These are all older studies, some of which are several decades old, and do not meet current standards for treatment studies. Treatment lasted 14 days in the RCTs and 10–30 days in the NRSs. Different treatment durations were not compared with one another. Only one prospective cohort study required that a patient have a positive CSF finding indicating a “probable” case of Lyme neuroborreliosis in order to be included in the study; all other studies based their inclusion criteria on a “possible” case of Lyme neuroborreliosis, which does not require the detection of inflammatory changes in CSF for a diagnosis and thus carries the risk of recruiting false positive cases. Penicillin G was investigated most frequently (5 studies), followed by ceftriaxone (4 studies) and doxycycline (2 studies). There were no studies on hydroxychloroquine, azithromycin, minocycline or carbapenem. Three studies compared several beta-lactam antibiotics with one another, one study compared beta-lactam antibiotics with doxycycline, and two studies investigated various treatment regimens. Apart from one cohort study, all studies showed a critical overall risk for bias. This pertained to the recruitment process, randomisation, blinding, confounding of baseline data and data evaluation and/or data reporting, so that the results can only be used to a very limited degree to recommend treatment. When comparing beta-lactam antibiotics with doxycycline, none of the studies – including a pooled retrospective analysis of three studies [247] – showed a statistically significant difference in terms of clinical outcomes, although the large confidence intervals limit this assertion. The same applies to the comparison of penicillin G with ceftriaxone. In one study, no side effects were reported in the penicillin G group, however the ceftriaxone group reported a moderate allergic skin reaction (n=1), an increase in liver enzymes (n=2) and asymptomatic gallbladder concretions (n=6) in the ceftriaxone group. The gallbladder concretions were detected by ultrasound screening in the ceftriaxone group, but this was not carried out in the penicillin comparison group. The side effects reported in the other studies could not be attributed to the respective interventions and could therefore not be analysed. Differentiated clinical recommendations cannot be derived from the limited study data. However, the prognosis for Lyme neuroborreliosis in children appears to be favourable across all studies. Poor outcomes or an inadequate response to treatment was rarely reported, regardless of the antibiotic used. 

In the past, the opinion was held that doxycycline should only be given to children over the age of 8 due to a yellowing of the permanent teeth. However, this is probably an inadmissible transference of results from classic tetracyclines to doxycycline, which differs significantly as a second-generation tetracycline: The effective dose is lower, it is administered less frequently per day, the calcium binding capacity is lower, and it is more fat-soluble [271]. Doxycycline is currently recommended regardless of age to treat infectious diseases, such as Rocky Mountain spotted fever, rickettsioses, plague, Q fever and other diseases, when no other comparable treatment is available and when the patient is allergic to beta-lactam [272].

More recent data now show that teeth do not become discoloured, even in children under 8 years of age [6], [7], [8], [9], [10]. Some authors still urge caution [273] and advise against prescribing doxycycline to treat young children if, as in the case of erythema migrans, a good alternative like amoxicillin is available [274]. The American Academy of Pediatrics (AAP) notes that a course of doxycycline can be administered regardless of age for up to 3 weeks [275]. However, only one study examined the discolouration of teeth when administered intravenously [8]. After initial hesitation, the Infectious Disease Society of America also endorsed the statement made by the AAP [276]. The initially called-for caution [273] and advice against treating young children with doxycycline when, as in the case of erythema migrans, a good alternative in the form of amoxicillin is available, in no way applies to Lyme neuroborreliosis [274].

A recent retrospective study uses parental interviews to investigate tooth discolouration in young children (20 months to 7 years) after doxycycline was used to treat Lyme borreliosis [277]. Here, 2/18 parents reported that their children experienced tooth discolouration. Compared to earlier studies [8], [9], however, there was neither a control group nor any dental/medical objectification of tooth discolouration. Due to the retrospective design and the interview element, it can be assumed that this method produced a “relevant recall bias”. Overall, the authors conclude that doxycycline is a generally well-tolerated substance that can be used to treat Lyme borreliosis in young children, but at the same time recognise the need for prospective observational studies [277].

This suggests that doxycycline can also be used to treat Lyme neuroborreliosis in children under the age of 8. If treatment is to begin intravenously due to symptoms of encephalomeningitis, such as vomiting or dysphagia, third-generation cephalosporins should be administered first. Once these symptoms have subsided, an oral doxycycline should be administered for a total of 2 or 3 weeks. 

#### Recommendations for treating children and adults

**16. Recommendation (reviewed in 2023)**

*Patients should be treated with antibiotics if diagnosed* *with Lyme neuroborreliosis with inflammatory ce* *re* *bro* *spinal fluid syndrome (“probable” or “confirmed” Lyme borreliosis) (section 3.4).*

Grade of recommendation ↑↑

EC

Level of consensus: 100% (16/16)

**17. Recommendation (reviewed in 2023)**

*In the case of a “possible” Lyme neuroborreliosis (CSF not available or inconspicuous) (section 3.4), antibiotic treatment can be considered after a thorough differential diagnosis and if there is no evidence of another disease.*

Grade of recommendation ↔

EC

Level of consensus: 100% (14/14)

**18. Recommendation (modified in 2023)**

*Antibiotic treatment should take place over a period of 14 days in the case of early Lyme neuroborreliosis *[1]*, *[2]*, *[16]*.*

Grade of recommendation ↑

Level 1a evidence

Level of consensus: 100% (16/16)

**19. Recommendation (modified in 2023)**

*Antibiotic treatment should take place over a period of 14 to 21 days in the case of late Lyme neuroborreliosis. See section 5.3, *[16]*, *[23]*, *[24]*, *[25]*, *[26]*.*

Grade of recommendation ↑

Level IV evidence

Level of consensus: 100% (15/15)

**13. Statement (reviewed in 2023)**

*Reference is made to the S2k Guideline “Cutaneous Lyme Borreliosis” (AWMF Register No. 013-044 *[51]*) for the treatment of polyneuropathy associated with acrodermatitis chronica atrophicans (ACA). *

EC

Level of consensus: 100% (15/15)

**20. Recommendation (reviewed in 2023)**

*If a distally symmetrical polyneuropathy is suspected as a manifestation of Lyme neuroborreliosis without accompanying ACA (rare in Europe), the same procedure recommended for “possible” Lyme neuroborreliosis can be used.*

Grade of recommendation ↔

EC

Level of consensus: 100% (16/16)

**21. Recommendation (reviewed in 2023)**

*Cerebral vasculitis resulting from Lyme borreliosis should be treated with antibiotics in accordance with the recommendations for “late” Lyme neuroborreliosis.*

Grade of recommendation ↑↑

EC

Level of consensus: 93% (13 yes, 1 no)

**22. Recommendation (reviewed in 2023)**

*Analogous to the recommendations for cerebral vasculitis of another aetiology (DGN S1 guideline on cerebral vasculitis, AWMF Register No. 030-085, *[269]*), the additional administration of steroids and/or 100 mg/d of ASA can be considered for cerebral vasculitis resulting from Lyme borreliosis. *

Grade of recommendation ↔

EC

Level of consensus: 100% (16/16)

**23. Recommendation (reviewed in 2023)**

*If corresponding symptoms or deficits are present, symptomatic treatment (physiotherapy, physical therapy, occupational therapy, speech therapy, neuropsychological training, psychosocial measures, analgesics, rehabilitative measures) should be carried out in addition to antibiotic treatment.*

Grade of recommendation ↑

EC

Level of consensus: 100% (16/16)

#### Recommendations for choosing antibiotics for children and adults

See Table 5 (Tab. 5).

**14. Statement (reviewed in 2023)**

*The choice of antibiotic should be made after weighing up individual patient aspects (allergies, other tolerability, pregnancy, method and frequency of application, etc.).*

[2]*, *[16]*, *[17]*, *[18]*, *[19]*, *[20]*, *[21]*, *[270]*, *[278]

Grade of recommendation ↑

EC

Level of consensus: 94% (15 yes, 1 no) 

**24. Recommendation (reviewed in 2023)**

*Early* *Lyme neuroborreliosis should be treated with one of the following antibiotics: doxycycline, ceftriaxone, cefotaxime, penicillin G.*

[2]*, *[16]*, *[17]*, *[18]*, *[19]*, *[20]*, *[21]*, *[22]

Grade of recommendation ↑↑

Level IIb evidence

Level of consensus: 94% (15 yes, 1 no)

**25. Recommendation (reviewed in 2023)**

*Late* *Lyme neuroborreliosis should be treated with one of the following antibiotics: doxycycline, ceftriaxone, cefotaxime, penicillin G.*

*Section 5.3 *[16]*, *[23]*, *[24]*, *[25]*, *[26]

Grade of recommendation ↑↑

Level IV evidence

Level of consensus: 94% (15 yes, 1 no)

#### Recommendations for monitoring the treatment of children and adults

**26. Recommendation (reviewed in 2023)**

*Treatment success should be assessed on the basis of the clinical symptoms.*

Grade of recommendation ↑↑

EC

Level of consensus: 100% (16/16)

**27. Recommendation (reviewed in 2023)**

*If clinical deterioration occurs during or after treatment, the differential diagnoses should be reviewed using an interdisciplinary approach.*

Grade of recommendation ↑

EC

Level of consensus: 100% (16/16)

**28. Recommendation (reviewed in 2023)**

*If a patient continues to have impairing symptoms* *6 months* *after treatment, CSF testing should be repeated; if there are doubts that the symptoms are improving, an earlier CSF follow-up analysis can be considered; if pleocytosis persists, a new course of antibiotic treatment should be carried out after other diagnoses have been ruled out.*

Grade of recommendation ↑

EC

Level of consensus: 100% (16/16)

**29. Recommendation (reviewed in 2023)**

*The following parameters should* *not* *be used to monitor treatment:*

- *Borrelia-specific antibody concentrations (and/or titres) in serum *
- *Borrelia-specific CSF/serum antibody index *
- *Oligoclonal bands in CSF *
- *Total protein in CSF *
- *Band pattern in Lyme immunoblot*

Grade of recommendation ↓↓

EC

Level of consensus: 100% (17/17)

## Notes

### Disclaimer: No liability for errors in DGN e.V. guidelines

The medical and scientific guidelines of the German Society of Neurology (DGN) e. V. are systematically developed to help physicians make decisions in specific situations. They are based on the latest scientific findings and tried-and-tested procedures and ensure greater safety in medicine. They also aim to take economic aspects into account. The “guidelines” are not legally binding for physicians; the medical judgement exercised when examining and treating individual patients is always determinative. Guidelines therefore have neither – in the case of deviations – a liability-creating, nor – in the case of compliance – a liability-releasing effect.

The members of each guideline group, the Association of the Scientific Medical Societies and the scientific medical societies organised within it, such as the DGN, compile and publish the guidelines of the professional societies with the greatest possible care. Nevertheless, they assume no legal responsibility for the accuracy of the content. Particularly in the case of dosage information for medicinal products or specific active substances, the information provided by the manufacturer and printed in package inserts, as well as the patient’s risk-benefit ratio and illnesses at the time of treatment must always be taken into account by the attending physician! The release from liability applies in particular to guidelines whose period of validity has expired. 

### Guideline information

This is the English version of the German DGN S3 Guideline “Neuroborreliose”, AWMF Register Number: 030/071 [279].

- Development Stage: S3
- Coordinators: Prof Dr Sebastian Rauer, PD Dr Stefan Kastenbauer
- Published by the German Society of Neurology (DGN)

#### Version

- AWMF Version Number: 6.0
- Fully revised: 30 April 2024
- Valid until: 29 April 2027
- Chapter: Inflammatory and pathogen-related diseases

#### ICD-10 numbers

A69.2+, G01*, G63.0*

#### Synonyms

None

### Methods of guideline development

This guideline is based on an update of Guideline No. 030/071 “Lyme Neuroborreliosis” (development stage S3), which was prepared by a panel of experts in 2018. The guideline was prepared in accordance with the methodological requirements of the Association of the Scientific Medical Societies (AWMF) for the development and further development of guidelines for diagnosis and treatment and corresponds to an S3 guideline according to the AWMF’s three-stage concept. The composition of the guideline panel was interdisciplinary (IDA). 

Uniform formulations are used to standardise the recommendations in the guideline. 

The following gradations apply:

- Strong recommendation: “shall” ↑↑
- Recommendation: “should” ↑
- Open recommendation: “may be considered” ↔
- Recommendation against an intervention: “should not” ↓
- Strong recommendation against an intervention: “shall not” ↓↓

For the full methodology, see the Guideline Report in Attachment 1. 

### Participating medical societies and organisations

#### Steering committee 

Prof. Dr. med. Sebastian Rauer (coordinator)

PD Dr. med. Stefan Kastenbauer (coordinator)

PD Dr. med. Rick Dersch (evidence process)

German Society of Neurology (DGN)

Prof. Dr. med. Heidelore Hofmann 

German Dermatology Society (DDG)

Dr. med. Volker Fingerle

German Society for Hygiene and Microbiology (DGHM)

Prof. Dr. med. Hans-Iko Huppertz

German Society of Paediatrics and Adolescent Medicine (DGKJ) and

German Society for Paediatric Infectious Diseases (DGPI)

Prof. Dr. med. Klaus-Peter Hunfeld

The German Society for Clinical Chemistry and Laboratory Medicine (DGKL) and

INSTAND

Prof. Dr. med. Andreas Krause

German Society for Rheumatology and Clinical Immunology (DGRh)

Prof. Dr. med. Bernd Salzberger

German Society for Infectious Diseases (DGI)

#### Consensus group

(Alphabetically) (The steering committee and the mandate holders from the Austrian and Swiss medical societies are part of the consensus group.)

Prof. Dr. med. Karl Bechter

German Association for Psychiatry, Psychotherapy and Psychosomatics (DGPPN)

Prof. Dr. med. Christian Bogdan

Paul Ehrlich Society for Infection Therapy (PEG)

Astrid Breinlinger (participation in 1^st^ consensus conference); stepped down on 13 October 2023, succeeded by: Georg Heidelmann (participation in 2^nd^ consensus conference) 

Association for borreliosis and TBE in Germany (BFBD)

Ursula Dahlem

Action against tick-borne infections in Germany (OnLyme-Aktion)

Prof. Dr. med. Michael H. Freitag

German Society of General Practice/Family Medicine (DEGAM)

PD Dr med. Gudrun Gossrau

German Pain Society

Prof Dr med. Constanze Hausteiner-Wiehle, Prof Dr med. Jonas Tesarz 

German Society for Psychosomatic Medicine and Medical Psychotherapy (DGPM) and

German Congress of Psychosomatic Medicine (DKPM)

Prof Dr med. Rainer Müller

German Society of Oto-Rhino-Laryngology, Head and Neck Surgery (DGHNO-KHC)

Prof Dr med. Monika A. Rieger

German Society for Occupational Medicine and Environmental Medicine (DGAUM)

Dr Herbert Rixecker

German Borreliosis Society (DBG)

Prof Dr med. Stefan Thurau

German Society of Ophthalmology (DOG)

Prof Dr rer. nat. Reinhard Wallich

German Society of Immunology (DGI)

Dr med. vet. Hendrik Wilking

Robert Koch Institute (RKI)

#### Expert advisory group

(appointed by the DGN guideline committee)

Prof Dr H. W. Pfister, Neurology Clinic, Ludwig Maximilians University Munich 

Private lecturer Dr B. Pfausler, University Clinic for Neurology – NICU, Medical University of Innsbruck, Austria (voting member of the consensus conference for the Austrian Society of Neurology) 

Prof Dr M. Sturzenegger, Department of Neurology, Inselspital, University of Bern, Switzerland (voting member of the consensus conference for the Swiss Neurological Society) 

#### Other

Young Neurology (junior organisation of the DGN) was invited by the DGN to take part in the consensus conferences as an observer. 

The German Cardiac Society – Cardiovascular Research (DGK) was asked to participate, but did not nominate a representative for the guideline update. 

No contact could be established with the German Tick-borne Disease Association (BZK) for the guideline update. 

#### Moderated by

Prof Dr med. Ina B. Kopp

AWMF Institute for Medical Knowledge Management 

#### The boards of the following medical societies and organisations have approved this guideline

- German Society of Neurology (DGN)
- German Dermatology Society (DDG)
- German Society of General Medicine/Family Medicine (DEGAM)
- German Society for Occupational and Environmental Medicine (DGAUM)
- German Society of Oto-Rhino-Laryngology, Head and Neck Surgery (DGHNO-KHC)
- German Society for Hygiene and Microbiology (DGHM)
- German Society for Immunology (DGfI)
- German Society for Infectious Diseases (DGI)
- German Society of Paediatrics and Adolescent Medicine (DGKJ)
- German Society for Clinical Chemistry and Laboratory Medicine (DGKL)
- German Society for Paediatric Infectious Diseases (DGPI)
- German Association for Psychiatry, Psychotherapy and Psychosomatics (DGPPN)
- German Society for Psychosomatic Medicine and Medical Psychotherapy (DGPM)
- German Congress of Psychosomatic Medicine (DKPM)
- German Society for Rheumatology and Clinical Immunology (DGRh)
- German Pain Society
- German Society of Ophthalmology (DOG)
- INSTAND, Society for Promoting Quality Assurance in Medical Laboratories
- Paul Ehrlich Society for Infection Therapy (PEG)
- Robert Koch Institute
- Swiss Neurological Society (SNG)
- Austrian Society of Neurology (ÖGN)
- German Borreliosis Society (DBG)

#### The boards of the following organisations have not approved this guideline

- Action against tick-borne infections in Germany (OnLyme-Aktion) (see Appendix 4 of the Guideline Report in Attachment 1 for the dissenting opinion)
- Association for borreliosis and TBE in Germany (BFBD) (see Appendix 5 of the Guideline Report in Attachment 1 for commentary)

### Competing interests

See Appendix 1 in Attachment 1.

## Supplementary Material

Guideline report

Appendices

## Figures and Tables

**Table 1:**
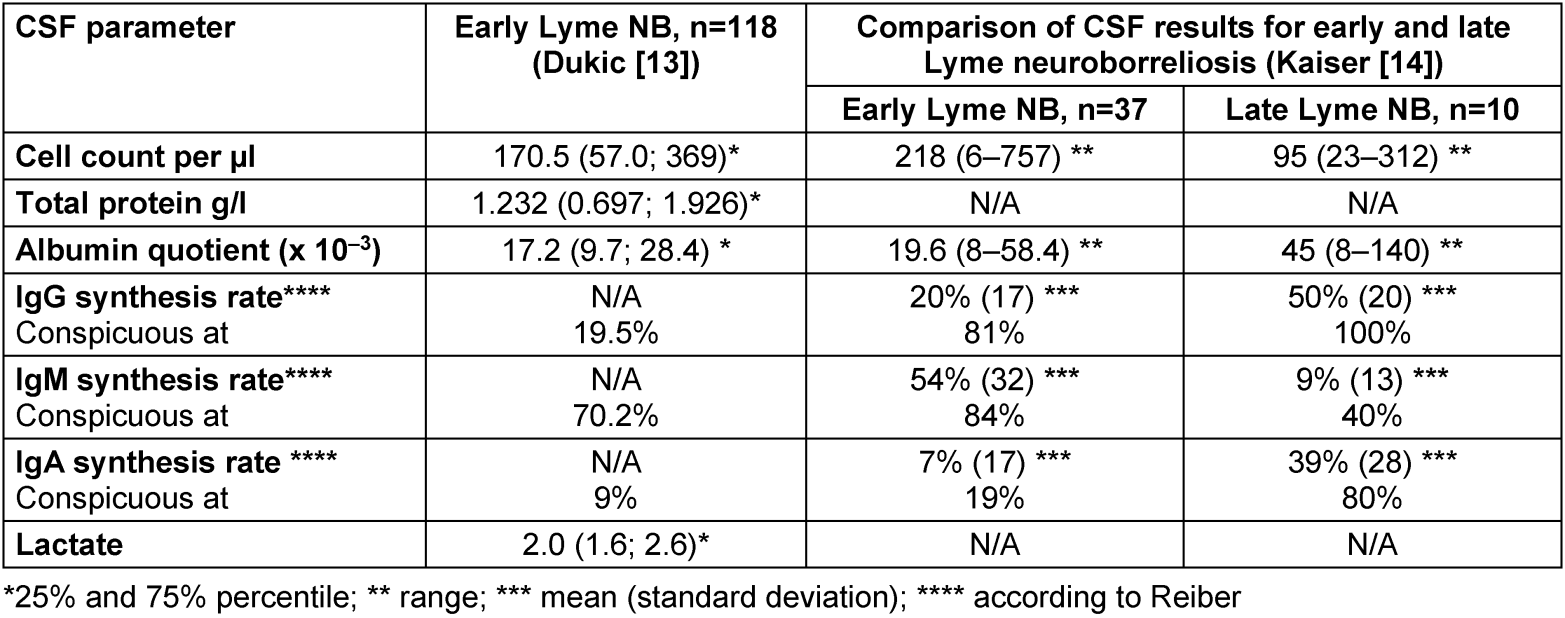
CSF results in early and late manifestations of Lyme neuroborreliosis prior to antibiotic treatment

**Table 2:**
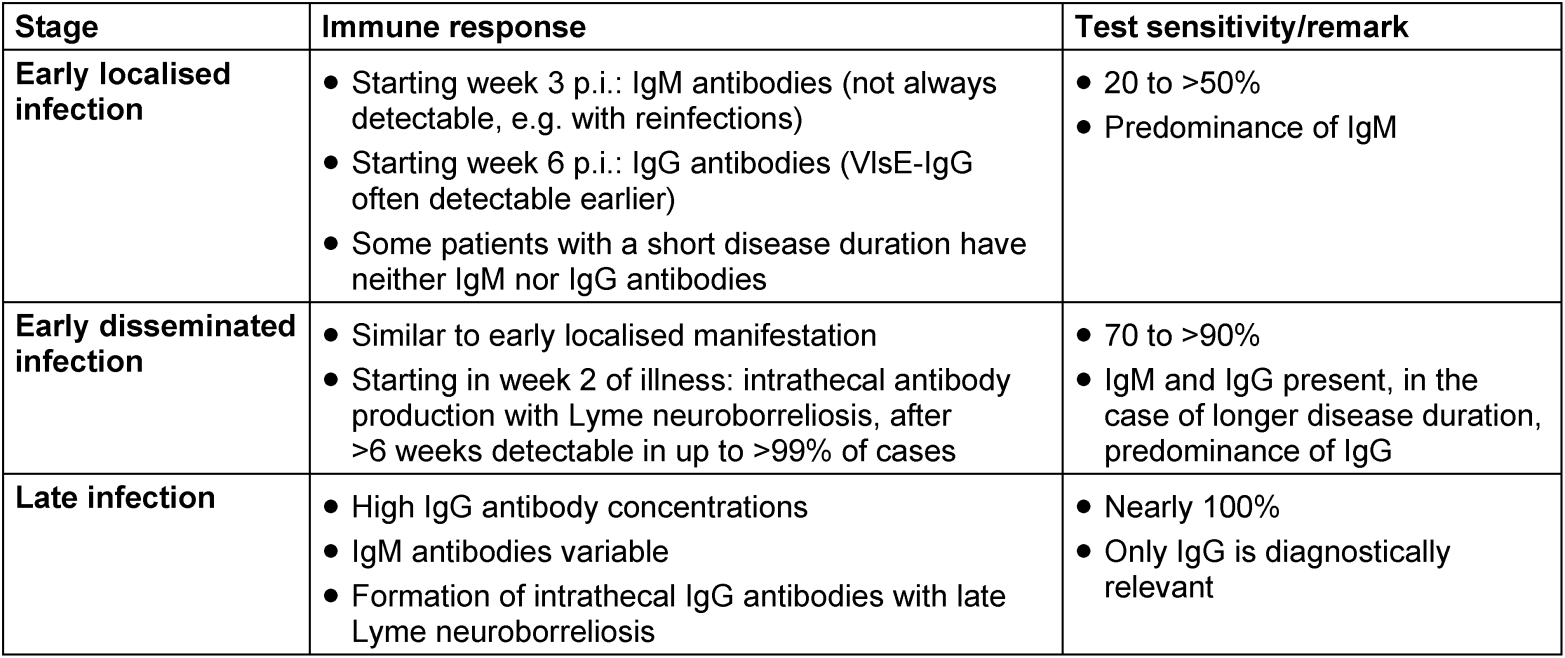
Antibody detection and test sensitivity in relation to the stage of disease (modified according to [12])

**Table 3:**
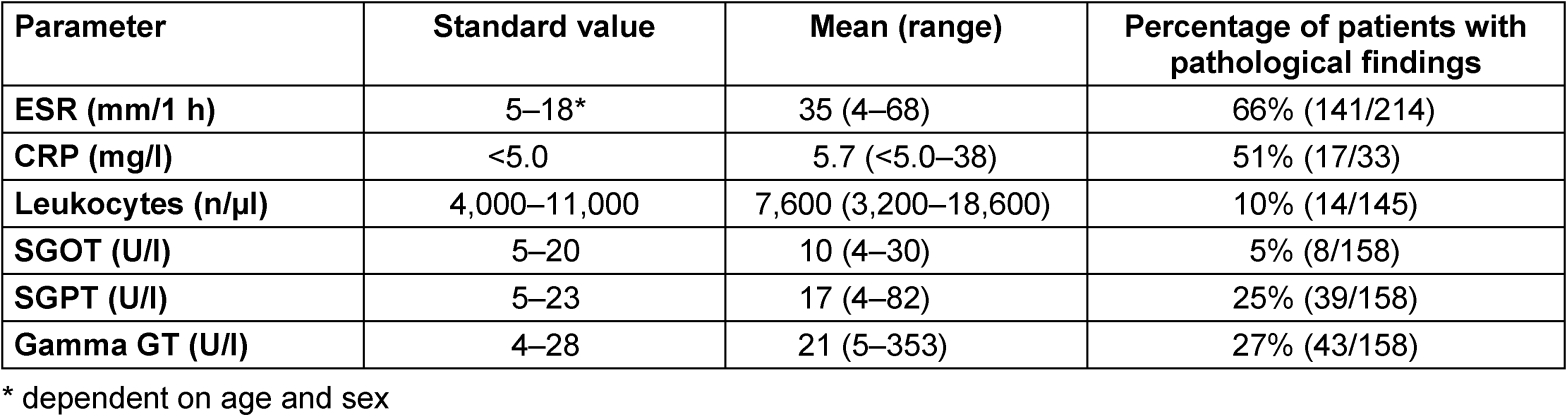
Routine laboratory testing parameters in patients with early and late manifestations of Lyme neuroborreliosis [15]

**Table 4:**
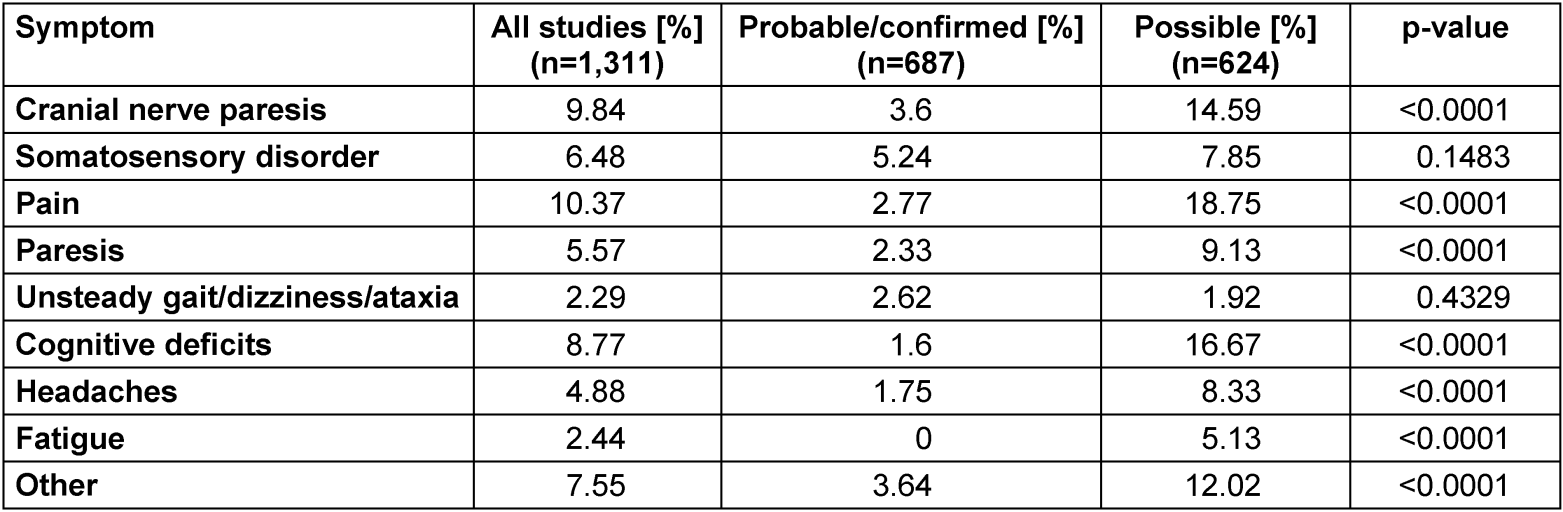
Systematic evaluation of the frequency of persistent symptoms following treatment for Lyme neuroborreliosis in relation to diagnostic certainty (probable/confirmed vs. possible), n=the number of evaluated patients (modified according to Dersch et al. [270])

**Table 5:**
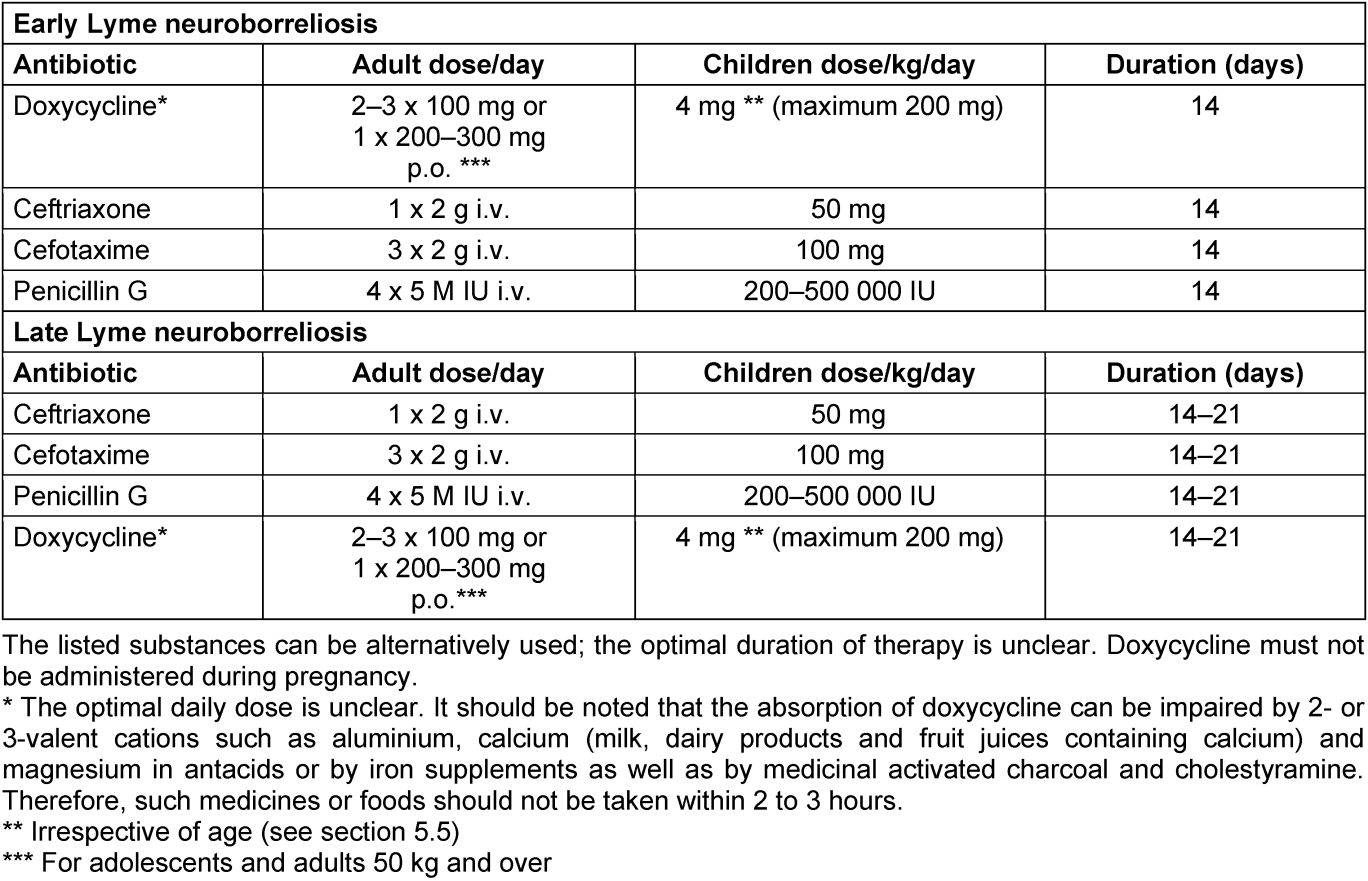
Overview of antibiotic treatment
