# Supplementary material for: Guidelines for diagnosis and treatment in neurology – Lyme neuroborreliosis
Source: Ger Med Sci. 2025 Oct 9;23:Doc13. doi: 10.3205/000349 (PMC12584707; doi:10.3205/000349)
Supplement: Guideline report [file GMS-23-13-s-001.pdf]

Attachment 1: Guideline report

Lyme neuroborreliosis

Guideline Report on Neuroborreliosis – Guidelines for Diagnosis and Treatment in Neurology

AWMF Register Number: 030/071

Coordinators: Prof Dr Sebastian Rauer
PD Dr Stefan Kastenbauer

Correspondence

sebastian.rauer@uniklinik-freiburg.de

On the internet

www.dgn.org
www.awmf.org

Disclaimer: No liability shall be assumed for errors in DGN e.V. guidelines

The medical and scientific guidelines of the German Society of Neurology (DGN) e. V. are systematically developed to help physicians make decisions in specific situations. They are based on the latest scientific findings and tried-and-tested procedures and ensure greater safety in medicine. They also aim to take economic aspects into account. The "guidelines" are not legally binding for physicians; the medical judgement exercised when examining and treating individual patients is always determinative. Guidelines therefore have neither – in the case of deviations – a liability-creating, nor – in the case of compliance – a liability-releasing effect.

The members of every guideline group, the Association of the Scientific Medical Societies and the scientific medical societies organised within it, such as the DGN, compile and publish the guidelines of the medical societies with the greatest possible care. Nevertheless, they assume no legal responsibility for the accuracy of the content. Particularly in the case of dosage information for medicinal products or specific active substances, the information provided by the manufacturer and printed in package inserts, as well as the patient's risk-benefit ratio and illnesses at the time of treatment must always be taken into account by the attending physician! The release from liability particularly applies to guidelines whose period of validity has expired.

Version

AWMF Version Number: 6.0
Fully revised: 30 April 2024
Valid until: 29 April 2027
Chapter: Inflammatory and pathogen-related disease

Keywords (German)

Lyme-Borreliose, Lyme-Neuroborreliose, *Borrelia-burgdorferi*-Infektion, Bannwarth-Syndrom, lymphozytäre Meningoradikulitis, Fazialisparese, Polyradikulitis, Meningitis, Enzephalomyelitis, Polyneuropathie, Schildzecken-Borreliose

Keywords (English)

Lyme disease, Lyme neuroborreliosis, *Borrelia burgdorferi* infection, Bannwarth's syndrome, lymphocytic meningoradiculitis, facial palsy, polyradiculitis, meningitis, encephalomyelitis, polyneuropathy, ixodid tick-borne borreliosis

Contents

1 Scope and purpose ... 3
1.1 Reasons for selecting the guideline topic ... 3
1.2 Objectives of the guideline ... 3
1.3 Patient target group ... 3
1.4 Area of care ... 3
1.5 Target users/audience ... 3
2 Composition of the guideline group: participation of interest groups ... 4
2.1 Guideline group representation: participating medical societies ... 4
2.2 Guideline group: participating patient organisations ... 4
2.3 Guideline group: participating medical societies and organisations ... 4
3 Methodological precision ... 6
3.1 Search, selection and evaluation of the scientific evidence (evidence-basing) ... 6
3.2 Formulating recommendations and structured consensus building ... 30
3.3 Adoption by the boards of the participating medical societies and organisations ... 31
4 Editorial independence ... 33
4.1 Guideline funding ... 33
4.2 Disclosing and dealing with conflicts of interest ... 33
5 Distribution and implementation ... 34
6 Period of validity and updating procedure ... 35
Appendix 1: Table on disclosing interests and dealing with conflicts of interest ... 36
Appendix 2: Consensus Meeting for the S3 Guideline on Lyme Neuroborreliosis ... 45
Appendix 3: Consensus Meeting for the S3 Guideline on Lyme Neuroborreliosis ... 46
Appendix 4: Dissenting opinion, OnLyme ... 47
Appendix 5: Comments by the BFBD ... 49
Literature ... 50

1 Scope and purpose

1.1 Reasons for selecting the guideline topic

Lyme borreliosis is the most common tick-borne infectious disease in Europe. The borrelia enter the skin during the sucking act of the *Ixodes ricinus* tick. There they are either inactivated by the innate immune system or cause a local infection. A small proportion of infected patients become ill. The most common result is inflammation of the skin, typically in the form of erythema migrans. As the disease progresses, the borrelia can disseminate and affect various organs such as the skin, nervous system, joints and heart. The nervous system is affected in 3–15% of all patients with Lyme borreliosis, usually manifesting as meningoradiculitis. Late or chronic cases are rare but can lead to encephalomyelitis with an unfavourable prognosis. In very rare instances, vasculitis affects the arteries to the brain which can result in consecutive strokes. If antibiotic treatment is not administered or is severely delayed, this may result in the persistence of serious residual neurological effects.

1.2 Objectives of the guideline

Aims of this Lyme neuroborreliosis guideline:

- Recommendations for confirming a clinical diagnosis; in particular, clarifying which clinical constellation warrants CSF testing
- Recommendations for stage-appropriate laboratory testing: serological detection of IgM and IgG Borrelia antibodies using a 2-step ELISA/immunoblot process
- Recommendations for determining Borrelia-specific intrathecal antibody synthesis (Borrelia-specific CSF/serum antibody index)
- Meaningful use of molecular testing and culture tests
- Recommendations for diagnostic certainty (possible, probable, confirmed Lyme neuroborreliosis)
- Treatment of early- and late-stage Lyme neuroborreliosis
- Recommendation for monitoring treatment
- Recommendations for treating persistent atypical or non-specific symptoms after antibiotic treatment
- Prevention of Lyme borreliosis
- Recommendations for observing the site of the tick bite
- Provision of patient information (appendix)
- The guideline does **not** include information on illnesses caused by *Borrelia recurrentis* (relapsing fever) or *Borrelia miyamotoi*.
- Issues relating to co-infections in connection with tick-borne diseases are **not** covered by the guideline

1.3 Patient target group

- Children and adults with a confirmed or suspected case of Lyme neuroborreliosis
- Patients presenting to a physician for diagnosis and treatment of Lyme neuroborreliosis
- Patients presenting to a physician with neurological symptoms that indicate Lyme neuroborreliosis
- Patients with persistent symptoms after antibiotic treatment for Lyme neuroborreliosis who need a differential diagnosis
- Patients presenting to a physician with questions about Lyme neuroborreliosis
- Patients presenting to a physician with a tick bite

1.4 Area of care

In- and outpatient care

1.5 Target users/audience

This guideline is directed at physicians in private practices and clinics specialising in various fields of medicine who are directly or indirectly involved in treating Lyme neuroborreliosis in children and adults. The wide range of interdisciplinary medical fields dealing with Lyme neuroborreliosis is reflected by the 22 medical societies involved in creating the guideline and by the participation of the Robert Koch Institute. The guideline also acts as a source

of information for patients and others interested in Lyme neuroborreliosis. These groups are represented in the consensus process by representatives from two patient and/or interest organisations.

2 Composition of the guideline group: participation of interest groups

2.1 Guideline group representation: participating medical societies

The German Society of Neurology (DGN), as the lead organiser, commissioned Prof Dr Sebastian Rauer of Freiburg, and PD. Dr Stefan Kastenbauer of Munich, to draft the initial update to the guideline, organise the consensus building, editorially develop the decisions of the guideline group as part of the consensus-building process, and produce the guideline report. The original idea of integrating this S3 guideline on Lyme neuroborreliosis as a second module in a future interdisciplinary S3 guideline on ‘Diagnosis and treatment of Lyme borreliosis’ has now been suspended.

A first draft of the guideline update, written after the key issues had been agreed upon, was first revised and assessed by the DGN expert group using a modified Delphi process. In addition to a national representative (Prof Dr H.-W. Pfister), the expert group also included a representative from Austria (PD Dr B. Pfausler) and a representative from Switzerland (Prof M. Sturzenegger).

Due to the high complexity and interdisciplinary nature of the topic, neurologists were joined by other physicians, a natural scientist (Prof. Dr rer. nat. R. Wallich) and a veterinarian (Dr med. vet. Hendrik Wilking) in developing the guideline and building consensus. In total, 22 medical societies, 18 of which are members of the AWMF, the Robert Koch Institute, and two patient organisations participated in developing the guideline. The German Cardiac Society - Cardiovascular Research (DGK) participated in the consensus-building process for the first edition of the guideline but was not involved in developing in the guideline update.

2.2 Guideline group: participating patient organisations

Representatives from two patient organisations were actively involved in the consensus-building process:

- Action Alliance Against Tick-Borne Infections Germany (OnLyme-Aktion)
- Association for Borreliosis and TBE in Germany (BFBD)

2.3 Guideline group: participating medical societies and organisations

Steering committee

Prof Dr med. Sebastian Rauer (coordinator)
PD Dr med. Stefan Kastenbauer (coordinator)
PD Dr med. Rick Dersch (evidence process)
German Society of Neurology (DGN)

Prof Dr med. Heidelore Hofmann
German Dermatology Society (DDG)

Dr med. Volker Fingerle
German Society for Hygiene and Microbiology (DGHM)

Prof Dr med. Hans-Iko Huppertz
German Society of Paediatrics and Adolescent Medicine (DGKJ) and
German Society for Paediatric Infectious Diseases (DGPI)

Prof Dr med. Klaus-Peter Hunfeld
The German Society for Clinical Chemistry and Laboratory Medicine (DGKL) and INSTAND

Prof Dr med. Andreas Krause
German Society for Rheumatology and Clinical Immunology (DGRh)

Prof Dr med. Bernd Salzberger
German Society for Infectious Diseases (DGI)

Consensus group

(Alphabetically) (The steering committee and the representatives from the Austrian and Swiss medical societies are part of the consensus group.)

Prof. Dr med. Karl Bechter
German Association for Psychiatry, Psychotherapy and Psychosomatics (DGPPN)

Prof Dr med. Christian Bogdan
Paul Ehrlich Society for Infection Therapy (PEG)

Astrid Breinlinger (participated in the 1st consensus meeting); stepped down on 13 October 2023, succeeded by:
Georg Heidelmann (participated in the 2nd consensus meeting)
Association for Borreliosis and TBE in Germany (BFBD)

Ursula Dahlem
Action Alliance Against Tick-Borne Infections Germany (OnLyme-Aktion)

Prof Dr med. Michael H. Freitag
German Society of General Practice/Family Medicine (DEGAM)

PD Dr med. Gudrun Gossrau
German Pain Society

Prof Dr med. Constanze Hausteiner-Wiehle, Prof Dr med. Jonas Tesarz
German Society for Psychosomatic Medicine and Medical Psychotherapy (DGPM) and
German Congress of Psychosomatic Medicine (DKPM)

Prof Dr med. Rainer Müller
German Society of Oto-Rhino-Laryngology, Head and Neck Surgery (DGHNO-KHC)

Prof Dr med. Monika A. Rieger
German Society for Occupational Medicine and Environmental Medicine (DGAUM)

Dr Herbert Rixecker
German Borreliosis Society (DBG)

Prof Dr med. Stefan Thurau
German Society of Ophthalmology (DOG)

Prof Dr rer. nat. Reinhard Wallich
German Society of Immunology (DGI)

Dr med. vet. Hendrik Wilking
Robert Koch Institute (RKI)

Expert Advisory Group
(appointed by the DGN guideline committee)

Prof Dr H. W. Pfister, Neurology Clinic, Ludwig Maximilians University Munich

Private lecturer Dr B. Pfausler, University Clinic for Neurology – NICU, Medical University of Innsbruck, Austria (voting member at the consensus meeting on behalf of the Austrian Society of Neurology)

Prof Dr M. Sturzenegger, Department of Neurology, Inselspital, University of Bern, Switzerland (voting member at the consensus meeting on behalf of the Swiss Neurological Society)

Other
Young Neurology (junior organisation of the DGN) was invited by the DGN to observe the consensus meeting.

Moderated by

Prof Dr med. Ina B. Kopp
AWMF Institute for Medical Knowledge Management

3 Methodological precision

3.1 Search, selection and evaluation of the scientific evidence (evidence-basing)

The systematic literature searches and evaluations were carried out by Dr Rick Dersch, who was responsible for conducting the systematic literature searches for the first edition of the guideline at the German Cochrane Centre Freiburg (Cochrane Germany). The process was conducted in line with the **PICO** framework (**P** = patient characteristics, clinical problem; **I** = intervention; **C** = comparison [comparison with alternatives]; O = outcome [target criteria]).

Formulating key issues

The key issues for the updated literature searches for the guideline update were taken from the first edition of the guideline. In addition to the aspects of efficacy and tolerability of antibiotic treatment, the key issues were expanded to include the efficacy and tolerability of supplementary steroid administration, as new study data have now been published on this topic. A systematic search and evaluation of the literature on the treatment of post-treatment Lyme disease syndrome (PTLDS) was carried out for the first time as part of the guideline update. The procedure for this was discussed and informally agreed to at the 1st consensus meeting on 18 July 2023.

a) *Definition of Lyme neuroborreliosis (**P**ICO) (taken from the first edition of the guideline):*

In infectiology, the microbiological detection of pathogens is considered the "gold standard" for defining an infectious disease. Since the test for detecting the pathogen in CSF is not sensitive enough for Lyme neuroborreliosis (10–30% sensitivity), diagnostic criteria have been agreed on which define the disease based on a combination of typical clinical symptoms, CSF findings and Borrelia serology as well as on recommendations from previous reviews and existing guidelines [1], [2], [3], [4], [5]. This definition makes a distinction between a "possible", "probable" and "confirmed" case of Lyme neuroborreliosis (see Section 3.11 of the guideline).

Discussion on the process mentioned above:

- "Seronegative cases" are not taken into account in clinical definitions (controversially discussed). Decision: These cases should be included in the descriptive review and discussed as part of the recommendations for transferability to extended patient groups.
- There is no standard validation process for serology tests. Decision: the type of serology test should be listed in the extracted studies (validity).
- "NB without CSF pleocytosis" in relation to possible biomarkers (e.g. cytokines like CXCL13).
  Decision: this should be included in the descriptive review.

Strong consensus: 13/13

*b) Intervention, comparison with alternatives (P**IC**O) (taken from the first edition of the guideline):*

- Antibiotic treatment vs. placebo
- Comparison of different types of antibiotic treatment with regard to: classes/substance, form of application, dosage, duration, drug levels
- The following antibiotics should be examined/compared: amoxicillin, azithromycin, cefotaxime, ceftriaxone, cefuroxime, clarithromycin, doxycycline, penicillin, metronidazole, minocycline, bactrim, erythromycin, quinolone, hydroxychloroquine
- Non-steroidal anti-inflammatory drugs vs. a placebo or no non-steroidal anti-inflammatory drugs
- Steroids vs. a placebo or no steroids
- Phytotherapeutic drugs should be included in the descriptive review (frankincense, curcumin, Artemisia annua, Samento, Banderol)

Strong consensus: 13/13

*c) Patient-relevant endpoints (outcomes) (PIC**O**) (taken from the first edition of the guideline):*

- Neurological condition (general)
- Neurological condition (specific)
  - Facial paresis
  - Hearing impairment
  - Vision impairment
  - Paresis of the extremities
  - Spinal symptoms
  - Dysesthesia/paraesthesia
  - Dizziness
- Scales
  - Quality of life (SF36)
  - Cognition (CVLT, TMT)
  - Depression (BDI)
  - Pain (SF36)
  - Fatigue (SF36)
  - Sleeping disorders
  - Ability/capacity to work %
  - Degree of disability

Endpoints should generally be measured using validated scales (the scales listed above should be regarded as examples).

Strong consensus: 13/13

Utilising existing guidelines on Lyme neuroborreliosis

An updated literature search was carried out in the electronic MEDLINE database (via OVID, 9 Feb.) and in the databases of three guideline networks with the aim of finding other relevant guidelines that could be used to update the content (the International Guideline Library of the Guidelines International Network [https://guidelines.ebmportal.com/], the National Institute for Health and Care Excellence [NICE, www.nice.org.uk/guidance/published?type=guidelines], the Association of Scientific Medical Societies [AWMF, www.awmf.org/leitlinien/leitlinien-suche.html]).

All of the guidelines published in German or English in these databases between 2015 and 2024 were considered (see Appendix for search strategy). A total of 148 entries were initially found, four of which were guidelines containing relevant information. The “Appraisal of Guidelines for Research and Evaluation II” survey (AGREE II) was used to analyse and evaluate the methodological quality of these guidelines [6]. Assessments are made in a total of 6 domains on the basis of predefined assessment criteria (scope, participation of interest groups, stringency of guideline development, clarity of design, applicability, and editorial independence). These domains are used to calculate a rating in per cent (%). Domain 3: “Stringency of guideline development” is particularly relevant, as it assesses the methodological aspects of the evidence-basing (systematic literature search, selection and evaluation of the literature, linking recommendations to the evidence). Guidelines that receive a rating of <50% in this domain are regarded as being of low methodological quality.

Table 1 contains the AGREE-II appraisal of the included guidelines. Two of the guidelines identified in the updated literature search had a rating of >50% in the domain “Stringency of guideline development”. Reviews of various international guidelines on Lyme borreliosis show a high degree of congruence between the respective recommendations [7], [8]. Since it was not possible to include any new significant evidence that had since been added to the guidelines being examined [9], [10], the existing recommendations were not adapted and our own updated literature search was conducted with an updated evidence synthesis.

Table 1: Guideline assessment using AGREEII

	Scope and purpose	Participation of interest groups	Stringency of the guideline development	Clarity of design	Applicability	Editorial independence
**Oteo et al. (Spain) [11]**	0.38	0.27	0.06	0.55	0	0.25
**Gocko et al. (France) [12]**	0.72	0.5	0.23	0.55	0	0.16
**Lantos et al. (USA) [13]**	1	1	1	1	0.66	1
**Cruickshank et al. (UK) [14]**	1	1	0.91	0.94	0.66	1

Appendix 1: Guideline search strategy in MEDLINE (OVID)

1. exp Lyme Disease/
2. lyme*.mp.
3. exp Borrelia burgdorferi Group/
4. borrel*.mp.
5. 1 or 2 or 3 or 4
6. exp practice guideline/
7. Health Planning Guidelines/
8. guideline*.ti.
9. (practice adj3 parameter*).ti,ab.
10. clinical protocols/
11. guidance.ti,ab.
12. care pathway*.ti,ab.
13. critical pathway/
14. (clinical adj3 pathway*).ti,ab.
15. algorithms/
16. consensus development conference.pt.
17. consensus development conference nih.pt.
18. 6 or 7 or 8 or 9 or 10 or 11 or 12 or 13 or 14 or 15 or 16 or 17
19. 5 and 18

Search strategy in guideline portals:
All results for “lyme” or “borrel*” were screened.

Utilising existing guidelines on "Post treatment Lyme disease syndrome" (PTLDS)

Two existing guidelines on PTLDS were submitted for evaluation by the patient organisations involved in the guideline creation process [15], [16]. Both documents are narrative reviews without a formalised evidence process. The formal AGREE II-based assessment of guideline quality revealed that both documents had clear limitations in all domains, but particularly in the methodologically relevant domain "Stringency of guideline development". According to the AGREE II assessment, both guidelines had an overall quality of <50%, which is why no further adaptation of the corresponding content was carried out. As part of the systematic literature search on PTLDS (search algorithm analogous to the chapter "Therapy for post-treatment Lyme disease syndrome" on page 23 of the guideline report), 1,274 entries were screened but no entries for evidence-based guidelines were found which could be used for further evaluation or adaptation.

Systematic literature search for pharmacological treatments for Lyme Neuroborreliosis in children and adults [17], [18]

In order to assess pharmacological treatments for Lyme neuroborreliosis, an updated literature search was performed for the period of 2015 to 2023 [18] using the same procedure used to conduct the literature search for the first edition of the guideline [19].

The search strategy and methodology of this systematic review were checked and published in advance as part of a peer-review process for the first edition of the guideline [19]. The literature was compiled and evaluated separately for adults and children.

Diagnosis had to be made in a transparent way using the internationally agreed case definitions (see above). The studies had to include data on the medicinal treatment of patients with Lyme neuroborreliosis and had to have a control group.

The literature search was conducted in three literature databases for the first edition of the guideline: MEDLINA (via Ovid), EMBASE (via Scopus) and the Cochrane Central Register of Controlled Trials (CENTRAL). Two databases were used in the literature search for the updated guideline: MEDLINE (via Ovid) and the Cochrane Central Register of Controlled Trials (CENTRAL).

The search strategy for the respective literature databases is listed in Appendix 2.

Appendix 2. Medline (OVID) search strategy

1. exp Lyme Disease/
2. lyme*.mp.
3. neuroborreliosis.mp.
4. borreli*.mp.
5. exp Borrelia/
6. (erythem* adj2 migran*).mp.
7. or/1-6
8. exp Brain/
9. brain*.mp.
10. mening*.mp.
11. spinal*.mp.
12. exp Nervous System Diseases/
13. encephal*.mp.
14. radiculi*.mp.
15. radiculo*.mp.
16. Facial Paralysis/
17. facial pal*.mp.
18. facial par*.mp.
19. Myelitis/
20. myel*.mp.
21. (nervous system adj5 dis*).mp.
22. neur*.mp.
23. polyneur*.mp.
24. polyradicul*.mp.
25. mononeur*.mp.
26. (nerve adj5 damage*).mp.
27. (nerve adj5 involvement).mp.
28. bannwarth*.mp.
29. vasculitis/
30. exp vasculitis, central nervous system/
31. vasculiti*.mp.
32. cranial nerve*.mp.
33. or/8-32
34. 7 and 33

SCOPUS search strategy

1. TITLE-ABS-KEY(lyme*) OR TITLE-ABS-KEY(neuroborreliosis) OR TITLE-ABS-KEY(borreli*) OR TITLE-ABS-KEY(erythema migrans)

2. TITLE-ABS-KEY(brain*) OR TITLE-ABS-KEY(mening*) OR TITLE-ABS-KEY(spinal*) OR TITLE-ABS-KEY(encephal*) OR TITLE-ABS-KEY(radiculi*) OR TITLE-ABS-KEY(radiculo*) OR TITLE-ABS-KEY(facial pal*) OR TITLE-ABS-KEY(facial par*) OR TITLE-ABS-KEY(myel*) OR TITLE-ABS-KEY(nervous system dis*) OR TITLE-ABS-KEY(neur*) OR TITLE-ABS-KEY(polyneur*) OR TITLE-ABS-KEY(polyradicul*) OR TITLE-ABS-KEY(mononeur*) OR TITLE-ABS-KEY(nerve AND damage*) OR TITLE-ABS-KEY(nerve AND involve*) OR TITLE-ABS-KEY(bannwarth*) OR TITLE-ABS-KEY(vasculiti*) OR TITLE-ABS-KEY(cranial nerve*)

3. 1 AND 2

Cochrane CENTRAL search strategy

1. MeSH descriptor: [Borrelia] explode all trees
2. MeSH descriptor: [Lyme Disease] explode all trees
3. *borreli*
4. erythem* near/2 migran*
5. lyme*
6. 1 OR 2 OR 3 OR 4 OR 5

Treating Lyme neuroborreliosis in children and adults – update [17], [18]

Evidence selection

In the course of updating the guideline, a literature search for antibiotic treatment of Lyme neuroborreliosis was performed in January 2023 for the period spanning 2015 to 2023. After screening 1,530 database entries, 7 new publications examining antibiotic treatment of Lyme neuroborreliosis were identified (see Figure 1, Table 2) [18]. Two of these were randomised controlled trials (RCTs) [9], [10], one was a prospective cohort study [5] and four were retrospective cohort studies [20], [22], [23]. All of the studies were assessed for risk of bias using either the Cochrane Risk of Bias Tool (RCTs) [24] or ROBINS I (cohort studies) [25]. One of the newly identified RCTs showed a low risk of bias in all domains [9], while the other RCT showed a high risk of bias for blinding [10]. Both RCTs were included in the respective meta-analyses. The cohort studies showed a high, i.e. critical overall risk of bias and are included or discussed in the relevant sections of the guideline [20], [21], [22], [23], [26].

Evidence assessment

The quality of the individual RCTs was examined and assessed using the Cochrane Risk of Bias Tool (www.handbook.cochrane.org). The quality of the non-randomised studies (cohort studies) was assessed using the ROBINS I tool [25]). The GRADE approach (Grading of Recommendations Assessment, Development and Evaluation) was used to evaluate the entire body of evidence [27]. For the meta-analysis of the existing studies, pooled effect estimates of treatment effects were calculated based on a fixed-effects model using the Mantel-Haenszel method. None of the studies on Lyme neuroborreliosis treatment compared antibiotic treatment with a placebo.

Creation of evidence tables

The GRADE approach was used to assess the quality of the evidence for the individual comparisons. The assessment of the individual comparisons is summarised in evidence tables (Tables 4–7).

Figure 1: PRISM chart of the updated literature search in MEDLINE (via OVID) and CENTRAL

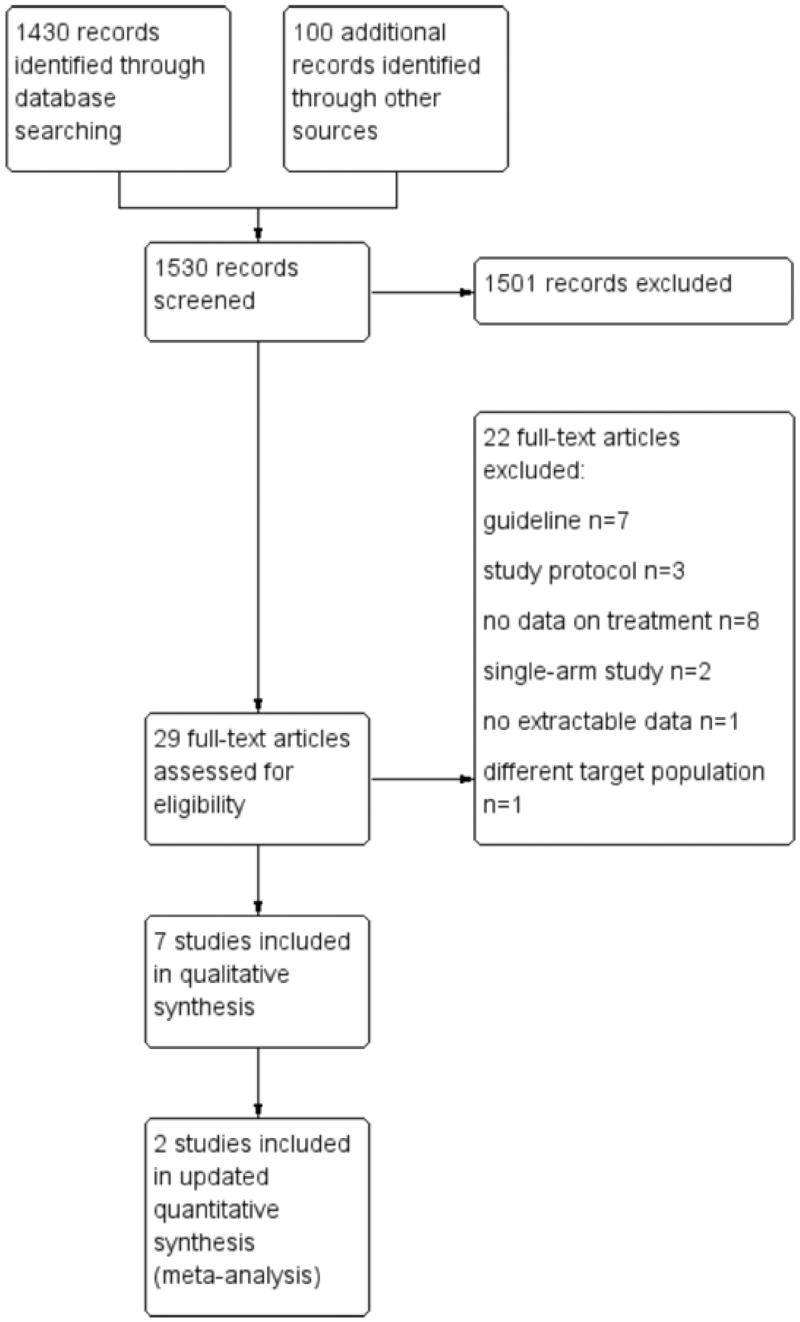

Table 2: Study characteristics

Study	Size	Case definition	Intervention	Treatment length	Country of origin
**RCTs**					
**Kortela 2021**	187	Confirmed (52%)<br>Possible (48%)	2 x 100 mg of doxycycline (n = 104)<br>2 g of ceftriaxone (n = 106)	Doxycycline 4 weeks<br>Ceftriaxone 3 weeks	Finland
**Solheim 2022**	121	Confirmed (83%)<br>Possible (17%)	200 mg of doxycycline	2 weeks vs.<br>6 weeks	Norway
**Prospective cohort studies**					
**Avellan 2021**	57	Confirmed (48%)<br>Probable (52%)	2 x 200 mg of doxycycline<br>2 x 200 mg of doxycycline + 60 mg of prednisolone	10 days<br>(60 mg of prednisolone for 5 days, then tapering down for 5 days)	Sweden
**Retrospective cohort studies**					
**Arnason 2022**	321	Confirmed (71%)<br>Possible (29%)	4mg/kg of doxycycline<br>50–100 mg/kg of ceftriaxone	10–14 days	Sweden
**Stupica 2021**	311	Confirmed (44%)<br>Possible (56%)	2 x 100 mg of doxycycline<br>2g of ceftriaxone	14 days	Slovenia
**Marques 2022**	44	Possible	Antibiotics (doxycycline or ceftriaxone)<br>vs. antibiotics + steroids (dosage unclear in each case)	Unclear	USA
**Jowett 2016**	51	Possible	Antibiotics vs.<br>antibiotics + steroids vs. antibiotics + steroids + acyclovir<br>(dosage unclear in each case)	Unclear	USA

Figure 2: Risk of bias - RCT

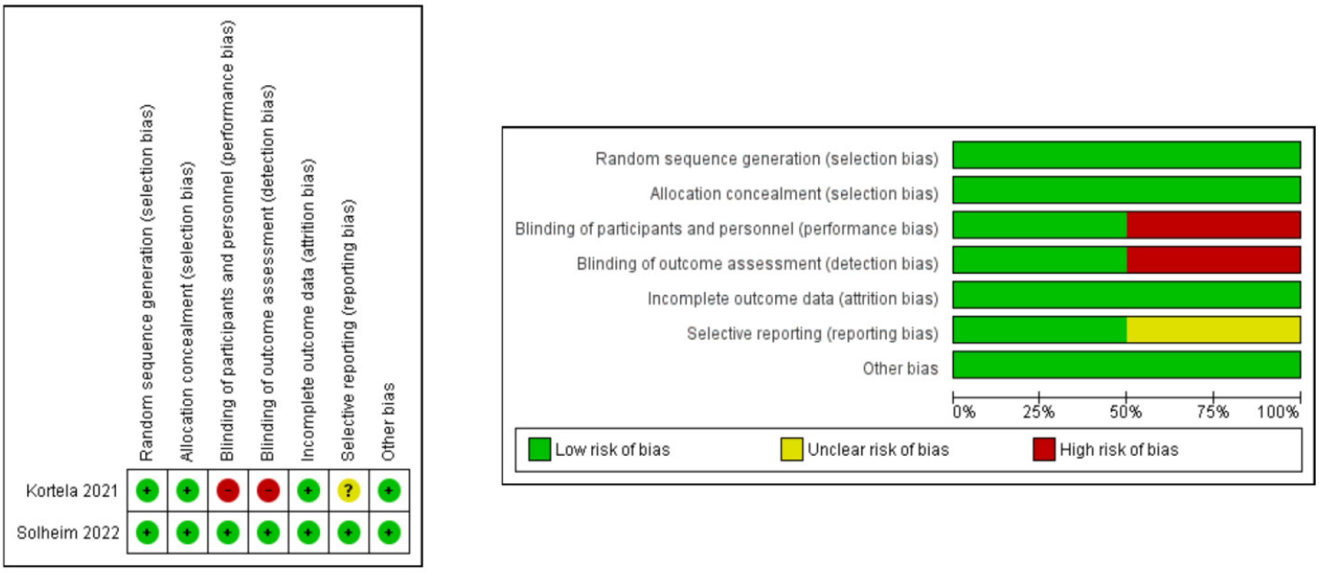

Table 3: Risk of bias – cohort studies

	Marques 2021	Avellan 2021	Jowett 2016	Stupica 2021	Arnason 2022
Bias due to confounding	critical	Critical	critical	Critical	Critical
Bias in selection of participants	low	low	critical	Low	Low
Bias in classification of interventions	critical	low	critical	critical	critical
Bias due to deviations from intended interventions	no information	low	no information	no information	no information
Bias due to missing data	serious	low	no information	serious	no information
Bias in measurement of outcomes	critical	critical	low	low	critical
Bias in selection of reported results	no information	no information	no information	no information	no information
Overall bias	critical	critical	critical	critical	critical

Assessment according to ROBINS-I (Sterne et al. 2016)

Figure 3: Results – beta lactams versus doxycycline

1.1 Residual neurological symptoms after 12 months

Study or Subgroup	Beta-lactams Events	Beta-lactams Total	Doxycycline Events	Doxycycline Total	Weight	Risk Ratio M-H, Random, 95% CI
Karlsson 1994	3	23	4	31	3.2%	1.01 [0.25, 4.08]
Kohlhepp 1989	11	36	12	39	13.6%	0.99 [0.50, 1.96]
Kortela 2021	36	84	35	82	50.9%	1.00 [0.71, 1.43]
Ljostad 2008	19	41	22	44	32.2%	0.93 [0.60, 1.44]
**Total (95% CI)**		**184**		**196**	**100.0%**	**0.98 [0.76, 1.26]**
Total events	69		73			

Heterogeneity: Tau² = 0.00; Chi² = 0.08, df = 3 (P = 0.99); I² = 0%
Test for overall effect: Z = 0.18 (P = 0.86)

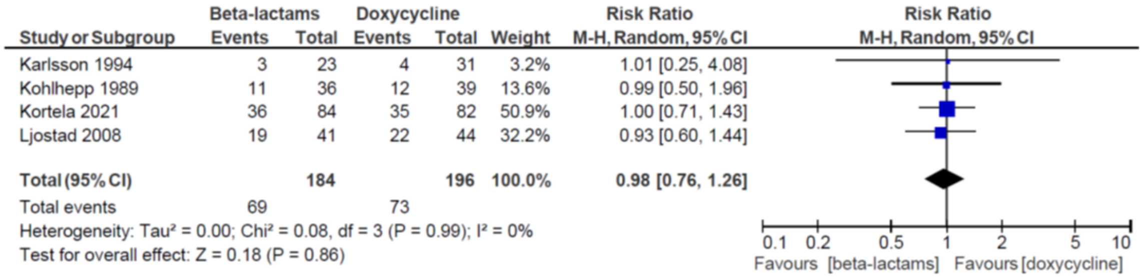

1.2 Any adverse events

Study or Subgroup	Beta-lactams Events	Beta-lactams Total	Doxycycline Events	Doxycycline Total	Weight	Risk Ratio M-H, Fixed, 95% CI
Karlsson 1994	3	23	4	31	9.7%	1.01 [0.25, 4.08]
Kortela 2021	9	93	5	82	15.2%	1.59 [0.55, 4.55]
Ljostad 2008	21	57	26	56	75.0%	0.79 [0.51, 1.23]
**Total (95% CI)**		**173**		**169**	**100.0%**	**0.94 [0.63, 1.39]**
Total events	33		35			

Heterogeneity: Chi² = 1.52, df = 2 (P = 0.47); I² = 0%
Test for overall effect: Z = 0.33 (P = 0.74)

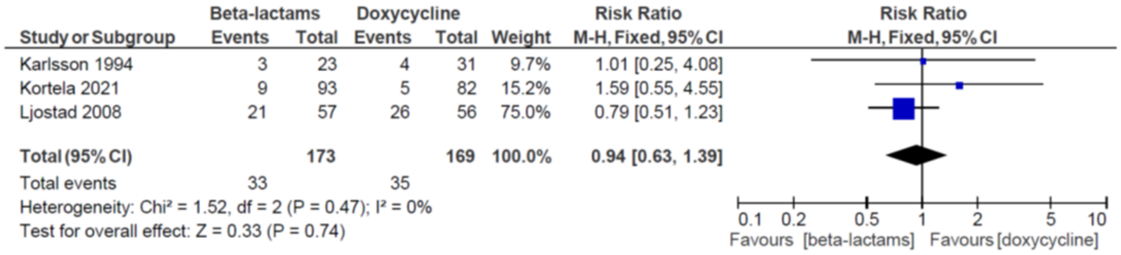

Figure 4: Results – additional steroids for fascial paresis in the context of Lyme neuroborreliosis

2 Antibiotics vs. antibiotics + steroids

2.1 Residual neurological symptoms after 12 months

Study or Subgroup	antibiotics Events	antibiotics Total	antibiotics + steroids Events	antibiotics + steroids Total	Risk Ratio M-H, Fixed, 95% CI
Avellan 2021	6	30	2	27	2.70 [0.59, 12.26]
Marques 2021	1	14	3	26	0.62 [0.07, 5.41]

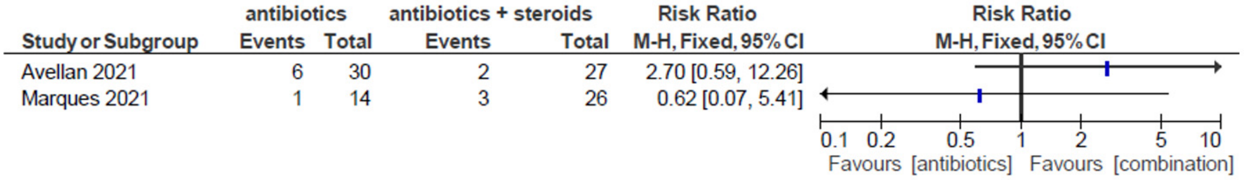

Figure 5: Results – treatment duration of 2 versus 6 weeks of doxycycline

3.2 Residual neurological symptoms after 12 months

	two weeks		six weeks		Risk Ratio
**Study or Subgroup**	**Events**	**Total**	**Events**	**Total**	**M-H, Fixed, 95% CI**
Solheim 2022	6	46	10	46	0.60 [0.24, 1.51]

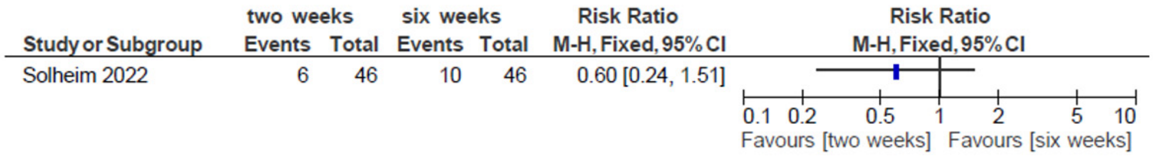

3.3 Residual neurological symptoms after 6 months

	two weeks		six weeks		Risk Ratio
**Study or Subgroup**	**Events**	**Total**	**Events**	**Total**	**M-H, Fixed, 95% CI**
Solheim 2022	12	52	11	51	1.07 [0.52, 2.20]

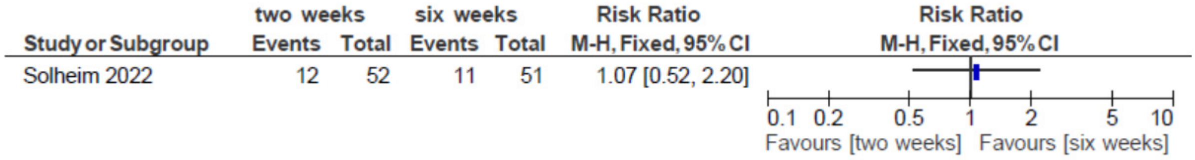

Figure 6: Results – 2 weeks of antibiotic treatment versus extended antibiotic treatment

4 Short vs. extended antibiotic treatment

4.1 Residual neurological symptoms after 12 months

	short		extended			Risk Ratio
**Study or Subgroup**	**Events**	**Total**	**Events**	**Total**	**Weight**	**M-H, Fixed, 95% CI**
Oksi 2007	17	72	14	75	57.8%	1.26 [0.67, 2.37]
Solheim 2022	6	46	10	46	42.2%	0.60 [0.24, 1.51]
**Total (95% CI)**		**118**		**121**	**100.0%**	**0.98 [0.59, 1.64]**
Total events	23		24			

Heterogeneity: Chi² = 1.71, df = 1 (P = 0.19); I² = 41%
Test for overall effect: Z = 0.06 (P = 0.95)

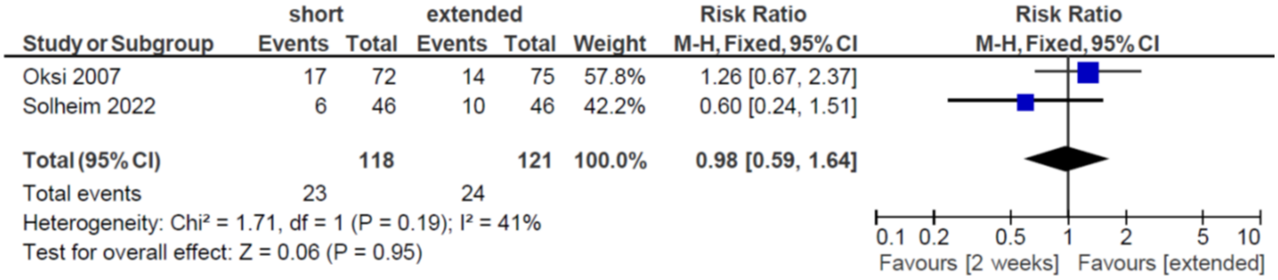

Table 4: GRADE – beta lactams versus doxycycline

Certainty assessment							Number of patients		Effects		Certainty
**No. of studies**	**Study design**	**Risk of bias**	**Inconsistency**	**Indirectness**	**Lack of precision**	**Other factors**	**Beta lactams**	**Doxycycline**	**Relative risk (95% CI)**	**Absolute (95% CI)**	
**Neurological symptoms > 12 months (follow-up period: 12 months)**											
4	Randomised controlled trials	Serious[a]	Not serious	Not serious	Serious[b]	None	69/184 (37.5%)	73/196 (37.2%)	**RR 0.98** (0.76 to 1.26	**7 fewer per 1,000** (from 89 fewer to 97 more)	⊕⊕◯◯ Low
**Adverse events**											
3	Randomised controlled trials	Serious[a]	Not serious	Not serious	Serious[b]	None	33/173 (19.1%)	35/169 (20.7%)	**RR 0.94** (0.63 to 1.39	**12 fewer per 1,000** (from 77 fewer to 81 more)	⊕⊕◯◯ Low

a: three non-blinded studies, partial bias due to non-disclosure of the allocation coverage and selective reporting
b: low case numbers

Table 5: GRADE – additional administration of steroids for fascial paresis in the context of Lyme neuroborreliosis

Certainty assessment							Number of patients		Effects	Certainty
No. of studies	Study design	Risk of bias	Inconsistency	Indirectness	Lack of precision	Other factors	Additional steroids	Monotherapy with antibiotics		
**Residual fascial paresis following treatment (dichotomous, follow-up period: 12 months)**										
2	Observational studies	Very serious[a]	Serious[b]	Serious[c]	Serious[d]	None	53	44	Not pooled (see text)	⊕◯◯◯ Very low
**Residual symptoms of fascial paresis following treatment (follow-up period: 12 months; assessed using: eFACE Dynamic)**										
1	Observational studies	Very serious[a]	Serious[e]	Serious[f]	Serious[d]	None	18	17	See text	⊕◯◯◯ Very low
**Residual symptoms of fascial paresis following treatment (follow-up period: 12 months; assessed using: eFACE Synkinesis)**										
1	Observational studies	Very serious[a]	Serious[e]	Serious[f]	Serious[d]	None	18	17	See text	⊕◯◯◯ Very low
**Residual symptoms of fascial paresis following treatment (follow-up period: 12 months; assessed using: eFACE Composite)**										
1	Observational studies	Very serious[a]	Serious[e]	Serious[f]	Serious[d]	None	18	17	See text	⊕◯◯◯ Very low

a. critical risk of bias in all of the studies according to ROBIN-I; b. relevant differences in effect estimates, two studies show no differences, one study shows disadvantage for steroids; c. very heterogenous patient population; d. low case numbers; e. single study; f. intervention insufficiently described

Table 6: GRADE – Doxycycline for 2 weeks versus 6 weeks

Certainty assessment							Number of patients		Effects		Certainty
**No. of studies**	**Study design**	**Risk of bias**	**Inconsistency**	**Indirectness**	**Lack of precision**	**Other factors**	**Doxycycline for 2 weeks**	**Doxycycline for 6 weeks**	**Relative (95% CI)**	**Absolute (95% CI)**	
**Residual neurological symptoms (follow-up period: 12 months)**											
1	Randomised controlled trial	Not serious	Serious[a]	Not serious	Serious[b]	None	6/46 (13.0%)	10/46 (21.7%)	**RR 0.60** (0.24 to 1.51)	**87 fewer per 1,000** (from 165 fewer to 111 more)	⊕⊕◯◯ Low

a. single study
b. low case numbers

Table 7: GRADE – 2 weeks of antibiotic treatment versus extended antibiotic treatment

Certainty assessment							Number of patients		Effects		Certainty
**No. of studies**	**Study design**	**Risk of bias**	**Inconsistency**	**Indirectness**	**Lack of precision**	**Other factors**	**2 weeks of antibiotics**	**Extended antibiotic treatment**	**Relative (95% CI)**	**Absolute (95% CI)**	
**Residual neurological symptoms (follow-up period: 12 months)**											
2	Randomised controlled trials	Not serious	Not serious	Very serious[a]	Serious[b]	None	23/118 (19.5%)	24/121 (19.8%)	**RR 0.98** (0.59 to 1.64)	**4 fewer per 1,000** (from 81 fewer to 127 more)	⨁⨁◯◯ Low

a. heterogenous population, one study includes additional patients with non-neurological manifestations of Lyme borreliosis. Case definition not consistently implemented in one study. Different types of antibiotics and treatment durations.
b. low case numbers, wide confidence interval

Treatment of Lyme neuroborreliosis in children [8], [28]

The evidence base for the pharmacological treatment of children with Lyme neuroborreliosis was also examined as part of the updated literature search [18]. No new studies were found for the period of 2015 to 2023 that specifically addressed the pharmacotherapy of Lyme neuroborreliosis in children and met the inclusion criteria of the systematic literature search [18]. Therefore, reference is made to the guideline report of the 1st edition of the S3 guideline on Lyme neuroborreliosis for the methodology used to determine the evidence for the pharmacotherapy of Lyme neuroborreliosis in children.

Treatment of post-treatment Lyme disease syndrome (PTLDS)

Evidence selection

A systematic literature search for relevant primary studies was conducted in the period up to July 2023 using a predefined search strategy (Appendix 3). Since PTLDS is a controversially discussed syndrome without a clear case definition, a broad range of items was deliberately used as part of the search strategy in order to adequately reflect the scale of the research field. The literature search was carried out using the Medline database (via OVID). The bibliographies of the included studies were also iteratively searched for possible relevant entries and integrated, and additional references submitted by the guideline panel were also included.

The literature search was conducted on 31 July 2023. The literature search yielded a total of 1,274 results. After screening the abstracts, a total of 48 entries were identified for a full-text screening. Of these, 9 entries were identified for qualitative analysis. The entries correspond to a total of 8 randomized controlled trials (RCTs). The characteristics of the studies are shown in Table 8.

Evidence evaluation

The risk of bias was assessed independently by two reviewers using the Cochrane Risk of Bias Tool. The 2011 version of this tool was used when updating the guideline to ensure compatibility with the pre-existing evidence evaluation.

Two RCTs had a high risk of bias (Cameron 2008, Murray 2022); data from these studies were therefore not used for further assessments. One RCT had an overall low risk of bias (Berende 2016). The assessment of the risk of bias is shown in Figure 9.

Relevant endpoints for the analysis were quality of life, fatigue, depression, cognition and side effects. Data reported in the identified studies were extracted. A quantitative evidence synthesis was not carried out due to significant differences in the inclusion criteria, the clinical interventions investigated, the duration of treatment, the measurement procedure, and the time at which the individual endpoints were assessed. Therefore, the results of the included studies were narratively summarised.

Creation of evidence tables

The GRADE approach was used to assess the quality of the evidence for the individual comparisons. If there were heterogenous inclusion criteria, no stringent case definition, as well as differences in clinical interventions and follow-up periods, the item “indirectness” received a “serious” rating in the GRADE assessment for all outcomes.

As outcomes were reported in a heterogeneous manner, no valid quantitative synthesis of the results could be made. Therefore, “lack of precision” received a “serious” rating for all outcomes. Heterogeneous effect estimates were identified in the individual studies for the outcome “depression”. Therefore, the item “inconsistency” was given a “serious” rating in accordance with the GRADE approach. The results for the other outcomes were similar for all of the studies included in the assessment, which is why “inconsistency” did not receive a lower rating here. The GRADE assessment was checked and validated by a second independent reviewer. The evaluation of evidence is presented in evidence tables (Tables 9 and 10).

Appendix 3. Search strategies in Medline (OVID)

1. exp Post-Lyme Disease Syndrome/
2. Post-Lyme disease syndrome.mp.
3. (post adj2 lyme).mp.
4. ptlds.mp.
5. (residual adj2 symptoms).mp.
6. (persisting adj 2 symptoms).mp.
7. (persistent adj2 symptoms).mp.
8. (residual adj2 symptoms).mp
9. exp Lyme Disease/
10. lyme disease.mp.
11. borreliosis.mp.
12. borrel*.mp.
13. neuroborreliosis.mp.
14. exp Lyme Neuroborreliosis/
15. 9 or 10 or 11 or 12 or 13 or 14
16. 5 or 6 or 7 or 8
17. 15 and 16
18. 1 or 2 or 3 or 4 or 17

Figure 7: PRISM chart of the literature search for PTLDS in MEDLINE (via OVID)

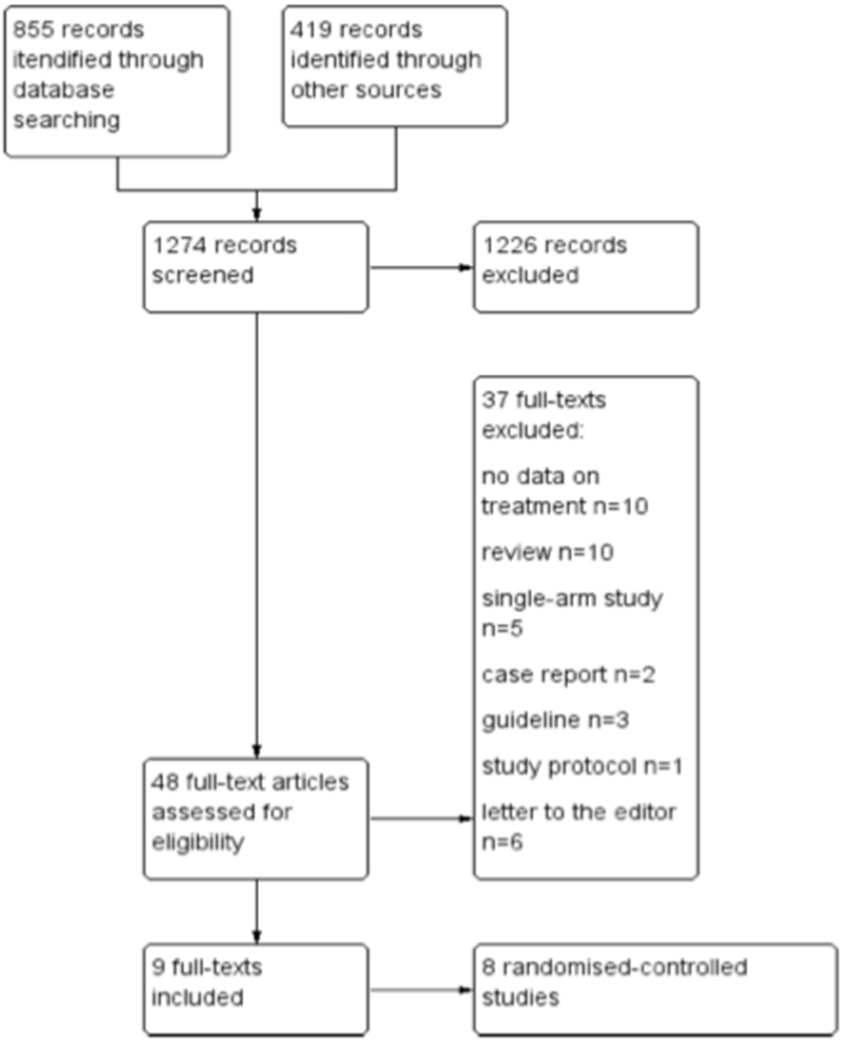

Table 8: Characteristics of the PTLDS studies

Study	Size	Case definition	Pre-treatment	Intervention	Treatment length	Country of origin
**Fallon 2007**	37	1. Status after erythema migrans or LB according to CDC criteria<br>and<br>positive or borderline ELISA with positive western blot<br>2. IgG western blot currently positive<br>3. Treatment with ceftriaxone i.v. for at least three weeks<br>4. Subjective memory impairment after onset of LB<br>5. Inconspicuous findings for Wechsler Memory Scale III	At least 3 weeks of ceftriaxone i.v.	Ceftriaxone i.v. vs. placebo	10 weeks	USA
Sjöwall 2012	15	1. LNB according to EUCALB criteria<br>2. >6 months of persistent symptoms (fatigue, fascial paresis, headaches, radiculitis, cognitive impairments)	Ceftriaxone or doxycycline for at least 10–14 days	2 x 100 mg of doxycycline orally vs. placebo	3 weeks	Sweden
**Berende 2016**	280	1. Persistent symptoms attributed to LB (musculoskeletal pain, arthritis/arthralgia, neuralgia, sensory disorders, dysaesthesia, neuropsychological abnormalities, cognitive disorders, fatigue)<br>2. Plausible timescale with respect to erythema migrans or another clinically confirmed LB or positive serology	30–40 days of antibiotics, unclear which type	2 weeks of ceftriaxone, then 12 weeks of doxycycline vs. clarithromycin + hydroxychloroquine vs. placebo	12 weeks	Netherlands

Study	Size	Case definition	Pre-treatment	Intervention	Treatment length	Country of origin
**Kaplan 2003**	129	1. Erythema migrans, early neurological/cardiological symptoms, radiculopathy, arthritis<br>2. Patient received antibiotic treatment<br>3. Persistent symptoms that occur within 6 months after LB lasting >6 months, e.g. musculoskeletal pain, cognitive impairment, radiculopathy, paraesthesia/dysesthesia/ fatigue<br>seronegative + seropositive patients included	Previous antibiotic treatment, duration/type unclear	2g of ceftriaxone i.v. for 30d, then 200 mg of doxycycline orally for 60d vs. placebo i.v. for 30d, then placebo orally for 60d	90 days	USA
**Klempner 2001**	107	1. Erythema migrans, early neurological/cardiological symptoms, radiculopathy, arthritis<br>2. Patient received guideline-compliant antibiotic treatment<br>3. Persistent symptoms that occur within 6 months after LB lasting >6 months, e.g. musculoskeletal pain, cognitive impairment, radiculopathy, paraesthesia/dysesthesia/ fatigue<br>seronegative + seropositive patients included	Previous antibiotic treatment, type unclear<br>Median duration 50 days (placebo) – 66 days (verum)	2g of ceftriaxone i.v. for 30d, then 2x100 mg of doxycycline orally for 60d vs. placebo i.v. for 30d, then placebo orally for 60d	90 days	USA
**Krupp 2003**	48	1. 18–70 years old<br>2. Erythema migrans or late manifestation of LB according to CDC criteria with positive ELISA and western blot<br>3. Patient received guideline-compliant antibiotic treatment for 6 months before entering study<br>4. Currently experiencing fatigue	Minimum of 3 weeks of 2 x 100mg of doxycycline or 3 x 500 mg of amoxicillin or 2 g/d of ceftriaxone	2 g of ceftriaxone i.v. vs. placebo	28 days	USA

Study	Size	Case definition	Pre-treatment	Intervention	Treatment length	Country of origin
**Cameron 2008**	84	1. >18 years old<br>2. Recurrence of LB symptoms after previously successful antibiotic treatment	All patients had received antibiotic treatment, duration and type not stated	3 x 1000 mg of amoxicillin vs. placebo	3 months	USA
**Murray 2022**	29	1. > 18 years old<br>2. Clinical diagnosis of LB at least 6 months before entering study<br>3. Initial guideline-compliant antibiotic treatment<br>4. Persistent symptoms that started within 6 months after LB diagnosis which have persisted >6 months<br>5. Persistent pain and fatigue	Guideline-compliant pre-treatment was inclusion criterion, but was not specified	Kundalini Yoga vs. waiting list	8 weeks	Netherlands

Figure 8: Risk of bias for PTLDS

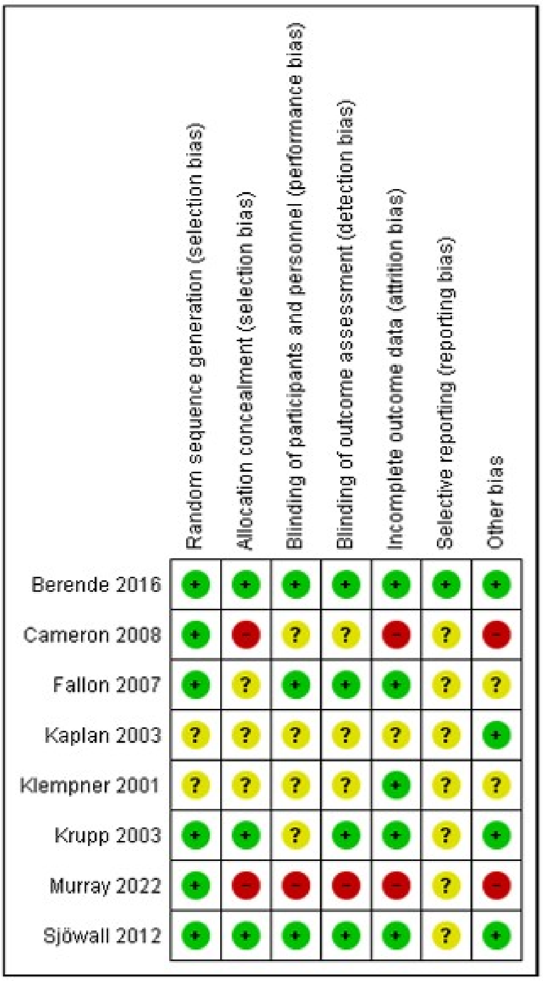

Table 9: GRADE assessment - effectiveness of antibiotics in the context of PTLDS

Certainty assessment								Result	Certainty
No. of studies	Study design	Risk of bias	Inconsistencies	Indirectness	Lack of precision	Antibiotics n=	Placebo n=		
**Fatigue (various means of measurement)**									
3	Randomised controlled trials	Not serious	Serious[a]	Serious[b]	Serious[c]	228	132	Two RCTs found no statistically significant difference with regard to fatigue (including one study with an overall low risk of bias).<br>One RCT found less fatigue after antibiotic treatment.	⊕◯◯◯ Very low
**Depression (assessed using: BDI)**									
2	Randomised controlled trials	Not serious	Not serious[a]	Serious[b]	Serious[c]	84	77	No statistically significant difference with regard to depression.	⊕⊕◯◯ Low
**Quality of life (assessed using: SF36)**									
4	Randomised controlled trials	Not serious	Not serious	Serious[b]	Serious[c]	274	183	No statistically significant difference with regard to quality of life.	⊕⊕◯◯ Low
**Cognition (various means of measurement)**									
4	Randomised controlled trials	Not serious	Not serious	Serious[b]	Serious[c]	292	197	No statistically significant difference with regard to cognition.	⊕⊕◯◯ Low

a. heterogeneity in terms of the direction of outcome
b. relevant difference in terms of case definition, intervention, treatment duration and follow-up period
c. pooled analysis not possible

Table 10: GRADE assessment – side effects of antibiotics for PTLDS

Certainty assessment						Number of patients		Effects	Certainty
No. of studies	Study design	Risk of bias	Inconsistency	Indirectness	Lack of precision	Antibiotics	Placebo	Relative risk (95% CI)	
**Side effects (AE)**									
4	RCTs	Not serious	Not serious	Very serious[a]	Serious[b]	111/284 (39.1%)	47/192 (24.5%)	**RR 1.47** (1.11-1.95)	⊕⊕◯◯ Low
**Serious side effects (SAE)**									
4	RCTs	Not serious	Not serious	Very serious[a]	Serious[c]	7/289 (2.4%)	3/202 (1.5%)	**RR 1.51** (0.44-5.12)	⊕◯◯◯ Very low

[a] relevant differences with regard to case definition, intervention, treatment duration and follow-up period
[b] low case numbers
[c] low case numbers and low event frequency

3.2 Formulating recommendations and structured consensus building

Formal consensus building: process and implementation

A first draft of the revised guideline was prepared by Prof. Sebastian Rauer following the updated systematic literature searches. This was then agreed on in the expert group using a modified Delphi process and put to a vote in the consensus group using the nominal group technique. Two consensus meetings were held which were independently moderated by the AWMF (Prof I. Kopp).

The steps of the nominal group technique were as follows:

- Presentation of the statements/recommendations to be agreed upon
- Opportunity to pose questions to the authors and moderator
- Silent note: which recommendation/grade of recommendation do you disagree with?
- Formulation of alternatives or, if necessary, amendments
- Registration of comments in the individual round robin sessions and
- Summary of the comments by the moderator
- Preliminary vote on each recommendation and all alternatives, determination of whether a discussion was needed
- Debate/discussion, development of the proposed solutions
- Final vote
- Steps repeated for each recommendation, final determination of the strength of consensus (“consensus”: >75% agreement, “strong consensus”: ≥ 95% agreement in relation to the number of participants with voting power)
- A presentation of the dissenting opinions was possible (even when a consensus/strong consensus had been established).
- The minutes of the meetings are available from the DNG’s guideline office.

**Classification of the strength of consensus**	
Strong consensus	> 95% of participants with voting power
Consensus	> 75 – 95% of participants with voting power
Majority consensus	> 50 – 75% of participants with voting power
No majority consensus	< 50 of participants with voting power

Consideration of benefits, side effects, relevant outcomes

The systematic review found that no reliable data on placebo-controlled treatments were available [17], [18]. However, there are analysable studies that compare different substance classes of antibiotics in terms of their efficacy and range of side effects [17], [18]. These are presented in Section 5 of the guideline. The relevant studies are summarised in a table in Appendices 3, 4 and 5 of the guideline. Appendix 8 of the guideline presents the evaluation of evidence of these studies using the GRADE approach.

Formulating recommendations and grading evidence and/or recommendations

Microbiological pathogen detection in culture or by microscopy or PCR is the gold standard in infectious disease medicine. There is no reliable gold standard for diagnosing Lyme neuroborreliosis (see Section 3.11. of the guideline) because pathogen detection in cerebrospinal fluid has a very low sensitivity (10–30%). As a result, consensus-based case definitions are used and controlled studies on diagnostic test procedures can only be carried out to a very limited degree from a methodological standpoint.

The evidence levels for all **treatment recommendations** are provided on the basis of the systematic reviews [17], [19], [28]. The basis for the presentation of evidence is the classification in the British NICE-SCIE guideline.

Levels of evidence: studies on treatment interventions

Ia Evidence from a meta-analysis containing at least three randomised controlled trials (RCTs)

Ib Evidence from at least one randomised controlled trial or one meta-analysis containing fewer than three RCTs

IIa Evidence from at least one methodologically sound controlled trial without randomisation

IIb Evidence from at least one methodologically sound, descriptive, quasi-experimental study.

III Evidence from methodologically sound, non-experimental observational studies, such as comparative studies, correlation studies and case studies

IV Evidence from expert committee reports or an expert opinion and/or the clinical experience of a recognised authority

Uniform formulations are used to standardise the recommendations in the guideline.
The following gradations apply:

Strong recommendation: “shall” ↑↑

Recommendation: “should” ↑

Open recommendation: “may be considered” ↔

Recommendation against an intervention: “should not” ↓

Strong recommendation against an intervention: “shall not” ↓↓

The recommendation grades were determined in formal consensus meetings. In addition to the quality of the underlying evidence, the following criteria were also explicitly taken into account:

- Consistency of the study findings, directness of evidence, precision of the effects estimate (see GRADE profile)
- Clinical relevance of the endpoints (outcomes) and effect strengths
- Risk-benefit ratio
- Legal considerations (approval status)
- Patient preferences
- Feasibility of implementation in clinical care.

Based on the consensus aspects mentioned above, a higher or lower grade of recommendation was given in individual cases relative to the level of evidence.

Participation and voting rights in the consensus meeting (web-based)

See Appendix 2 and Appendix 3 of the Guideline Report

3.3 Adoption by the boards of the participating medical societies and organisations

3.3.1 Feedback and suggestions from the medical societies on the first version of the manuscript

Feedback from 3 medical societies (DGKJ and DGPI were represented by a joint reviewer) on the first version of the guideline is summarised below. The suggestions made by the reviewers were largely implemented:

a) DGKJ and DGPI: On the recommendation of administering doxycycline to children under 8 years of age: The remark that this treatment is off label and requires written parental consent has been added. The publication by Brown et al. [29] was included in the section and discussed. The suggestion to change the citation format was forwarded to the DGN’s editorial office for review.

b) Comments by DGHNO:

1) *Geschmack/Geschmackssinn/Geschmacksstörungen – replaced by Schmecksinn/Schmeckstörungen*
2) *Recommendation 15: "Administering steroids in addition to antibiotics for fascial paresis for Lyme neuroborreliosis is not recommended."*
   - here there is no classification suitable for everyday clinical use: Borreliosis confirmation takes a considerable amount of time; however, steroids must be administered within 72 hours in the case of idiopathic paresis.
   It should therefore read: ... If steroids have already been administered, they should be discontinued if Lyme neuroborreliosis is confirmed as being the cause of the facial paresis (by the way: [the German spelling is] with a z, not with a c*).*
3) *On page 20 under 2.5 paragraph 2, instead of "hearing loss, dizziness", it would be better to write "acute sensorineural hearing loss and peripheral vestibulopathy".*

Response from the guideline coordinators: Points 1) and 3) have been incorporated into the text in line with the recommendations by the DGHNO. Point 2) overlaps with DEGAM's feedback and a response can be found under c).

c) Comments by DEGAM: *...I am not entirely convinced by the negative recommendation against administering cortisone for facial paresis. The justification based on one prospective and two retrospective cohort studies is too weak for me. There is otherwise good evidence that corticoids are beneficial for fascial paresis. If the recommendation is to be retained, it needs to be clearer that there is a confirmed correlation between the paresis and the Lyme neuroborreliosis (which, according to my clinical experience, is not usually the case).*

Response from the guideline coordinators: The negative recommendation regarding the administration of steroids for fascial paresis in the context of Lyme neuroborreliosis was revised in line with the suggestions. In particular, the recommendation explicitly pointed out that if steroids are not administered, the diagnosis of Lyme neuroborreliosis should be supported or confirmed by corresponding CSF findings in the sense of "probable" or "confirmed" Lyme neuroborreliosis (see Section 3.10). The experts from both medical societies agreed with this revision.

d) The statements by the Action Alliance Against Tick-Borne Infections Germany (OnLyme-Aktion) and the Association for Borreliosis and TBE in Germany (BFBD) can be found in Appendices 4 and 5 at the end of the Guideline Report.

3.3.2 The boards of the following medical societies and organisations have approved the guideline

- German Society of Neurology (DGN)
- German Dermatology Society (DDG)
- German Society of General Practice/Family Medicine (DEGAM)
- German Society for Occupational Medicine and Environmental Medicine (DGAUM)
- German Society of Oto-Rhino-Laryngology, Head and Neck Surgery (DGHNO-KHC)
- German Society for Hygiene and Microbiology (DGHM)
- German Society for Immunology (DGfI)
- German Society for Infectious Diseases (DGI)
- German Society of Paediatrics and Adolescent Medicine (DGKJ)
- German Society for Clinical Chemistry and Laboratory Medicine (DGKL)
- German Society for Paediatric Infectious Diseases (DGPI)
- German Association for Psychiatry, Psychotherapy and Psychosomatics (DGPPN)
- German Society for Psychosomatic Medicine and Medical Psychotherapy (DGPM)
- German Congress of Psychosomatic Medicine (DKPM)
- German Society for Rheumatology and Clinical Immunology (DGRh)
- German Pain Society
- German Society of Ophthalmology (DOG)
- INSTAND e. V., Society for Promoting Quality Assurance in Medical Laboratories
- Paul Ehrlich Society for Infection Therapy (PEG)
- The Robert Koch Institute
- Swiss Neurological Society (SNG)
- Austrian Society of Neurology (ÖGN)
- German Borreliosis Society (DBG)

3.3.3 The boards of the following organisations have not approved this guideline

- The Action Alliance Against Tick-Borne Infections Germany (OnLyme-Aktion) (see Appendix 4 for the dissenting opinion)
- The Association for Borreliosis and TBE in Germany (BFBD)
(see Appendix 5 for commentary)

4 Editorial independence

4.1 Guideline funding

Significant costs were incurred in preparing this guideline due to the work of PD Dr Rick Dersch in conducting the systematic reviews for the guideline update. DGN provided € 12,000 of funding for this work. There was no financial support over and above the funding mentioned here.

4.2 Disclosing and dealing with conflicts of interest

When work began on updating the guideline, the interests of all persons involved in the guideline (members of the steering group, the group of experts, the consensus group) were documented on a structured AWMF form (website for declarations of interest). The interests were assessed independently by 2 members of the guideline group (Prof Dr Reinhard Wallich and PD Dr Rick Dersch) at the behest of the DGN. This assessment is summarised in a table at the end of this report. The interests of Prof. Sebastian Rauer and PD Dr Stefan Kastenbauer were assessed by conflict-of-interest specialists at DGN.

The information was analysed with regard to connection to the topic, relevance of the topic, type and intensity of the relationship, and the amount of remuneration.

Assessment criteria

The following was categorised as *minor* conflicts of interest: lectures and papers on products from the pharmaceutical industry or third-party funding from state subsidies, which are recommended in the guideline.

The following was deemed to be a *moderate* conflict of interest: AdBoard, consulting and reviewing interests in pharmaceutical products that are discussed in the guideline and holding a responsible position in relation to third-party industry funding that is recommended in the guideline.

The following were categorised as *high* conflicts of interest: ownership interests; ownership of business shares; patent ownership for processes or products related to the guideline; family relationships with a company that markets a product that is covered in the guideline.

Statement by the panel on the assessment of conflicts of interest

Sebastian Rauer: His income from Data Safety and Monitoring Board (DSMB) memberships for a Lyme borreliosis vaccination and his 50% co-ownership in the company ravo, which manufactures diagnostic tests for various infectious diseases including Lyme borreliosis are topically related to the guideline on Lyme borreliosis.

RE vaccination: This primary prophylactic topic is not discussed in the guideline and does not fall under the category of disease diagnosis or treatment. Furthermore, Mr Rauer is not acting as an investigator with a possible interest in the outcome of the study, but as an observing DSMB member. Assessment: no conflict with the guideline topic.

RE the company ravo: Products from this company play a role in diagnosing the disease. The laboratory-based confirmation of Lyme neuroborreliosis (AI serum/CSF) is a topic of the guideline. A second guideline coordinator has therefore been appointed alongside Mr Rauer. Mr Rauer was not allowed to take part in deliberations or to vote on issues relating to the laboratory testing of Lyme borreliosis or neuroborreliosis. Assessment of the degree of the conflict of interest: “high” with respect to diagnostic testing.

However, it should also be emphasised that, due to his many years of experience (see previous DGN assessments in the 2018 version of the guideline), Professor Rauer is in no way personally suspected of having any relevant conflict of interest as an author and, as **the** national expert on the topic, cannot be excluded from the group of authors of such a guideline.

Dr Stephan Kastenbauer (no interests) was named the 2nd coordinator by the DGN and voted on behalf of the DGN.

Professor H. Hofmann received lecture fees on the topic of Lyme borreliosis from the pharmaceutical industry. The degree of her conflict of interest was categorised as "low", which is why her involvement in the consensus process was not restricted.

Four members of the guideline group stated that they had received fees as industry consultants. No connection to the topic of the guideline was found for two of these members (Prof. Dr A. Krause and Prof. Dr M. Mehling). In the case of Prof. Dr H. P. Hunfeld, the fees were connected to specific commercial tests, including Lyme borreliosis tests. Since no specific diagnostic tests were discussed, evaluated or recommended in the guideline, his neutrality was not regarded as being affected in this respect and therefore no restrictions on his voting rights were imposed. Dr V. Fingerle was briefly a member and PD Dr Dersch remains a member of an advisory board for vaccine development in the pharmaceutical industry. As no vaccine has been approved or is available for use in humans, there are no recommendations related to vaccines in the guideline. Therefore, this activity is not regarded as affecting their neutrality and no restrictions were therefore imposed. Furthermore, PD Dr Dersch was not entitled to vote in the consensus process, as PD Dr Kastenbauer voted on behalf of the DGN.

The risk of bias due to conflicts of interest was countered by:

- An interdisciplinary, pluralistic composition of the guideline group with the involvement of representatives with different points of view
- A systematic search and evaluation of evidence analogous to the methods used by the German Cochrane Centre
- Structured consensus-building that was moderated by Prof Ina Kopp, an independent guideline consultant from the AWMF

Because there is an overwhelming majority of conflict-free members in the guideline group (see table for summary), the measures to limit potential conflicts of interest are considered to have been sufficiently met, ensuring that there was independent decision-making in the preparation of the guideline in accordance with the criteria of the DGN and AMWF. The group of authors is considered to be well-balanced.

5 Distribution and implementation

Concept for distributing and implementing the guideline

Websites of the AWMF and DGN; presentation of the guideline at medical conferences.

Supporting material for applying the guideline

Supporting literature from the evidence process: [17], [18], [19], [28], [30], [31]

Discussion on possible organisational and/or financial obstacles to applying the guideline recommendations

Applying the guideline recommendations

As the recommended diagnostic tests and treatment can be carried out on either an inpatient or outpatient basis depending on the severity of the symptoms, and as the recommended antibiotics can be administered both orally and intravenously, there are unlikely to be any organisational problems in implementing the recommendations. As the recommended antibiotics are also available in generic form, the insurance companies should have no difficulties with respect to applying the guideline.

6 Period of validity and updating procedure

The guideline is valid for 3 years from the date of the revision (until 29 April 2027); a literature search for available systematic reviews will be carried out for the period following this and systematically evaluated 6 months before the guideline expires.

The updated manuscript will be discussed as part of a new consensus process and the key recommendations will be reviewed on this basis to ensure that they are up to date.

Prof Dr S. Rauer and PD Dr R. Dersch are responsible for updating the guideline in consultation with the DGN's Guideline Steering Group.

The Guideline Editorial Office at the DGN can be contacted at: leitlinien@dgn.org

Appendix 1: Table on disclosing interests and dealing with conflicts of interest

Disclosures of interest are summarised in the table below alongside the results of the conflict-of-interest assessment and the measures which were approved of by the guideline group, following a discussion of the issues, and implemented at the consensus meeting.

Guideline coordinators: Rauer, Sebastian; Kastenbauer, Stefan
Guideline: Lyme neuroborreliosis
Register number: 030/071

	**Activity as a consultant and/ or expert**	**Participation in a scientific advisory board**	**Paid lecturing or training activity**	**Paid author-ship or co-authorship**	**Research projects/ clinical studies**	**Ownership interests (patent, copyright, shares)**	**Indirect interests**	**Guideline topics affected by COI**<br>**Degree of relevance**<br>**Consequence**
Prof. Dr med. Bechter, Karl	Wellcome Trust London	Journal of Affective Disorders Reports, Elsevier Publishing House	Various scientific conferences	Various scientific journals	EU study MOODSTRATIFICATION, donation to the CSF Studies research project by Dr Hans Huber, Stuttgart	None	Memberships: various scientific societies that conduct psychiatric and especially psycho-immunological research, e.g. DGPPN, DGBP, EPA, ECNP, WPA; chair of the CSF Research Department at DGBP, co-chair of the WPA Immunology Psychiatry Department, member of the board of trustees of the neuropsychiatric congress Mind INPC Pula<br>Scientific activities: psychiatric, especially psycho-immunological research, reviewer of articles submitted to various scientific journals, conference organiser<br>Clinical activities: psychiatric/ psychotherapeutic outpatient treatment<br>Involvement in further education/training: Psychoimmunology Expert Meetings for 20 years (see www.psychoimmunology-experts.de)<br>Mind INPC Pula congresses	None<br>None<br>None
Prof. Dr Bogdan, Christian	German Research Foundation (DFG), Leibniz-Gemeinschaft	AdBoard: advising the PEI on scientific issues and further development	Bavarian Medical Association	Springer Publishing House, Thieme Publishing House	German Research Foundation (DFG), Manfred Roth Foundation	National Institutes of Health, Bethesda, USA	Memberships: president of the Paul Ehrlich Society for Infection Therapy (PEG), Standing Committee on Vaccination (STIKO) at the Robert Koch Institute, German Society for Immunology, German Society for Hygiene and Microbiology<br>Scientific activities: basic research on infection immunology (activation	None<br>None<br>None

	Activity as a consultant and/ or expert	Participation in a scientific advisory board	Paid lecturing or training activity	Paid author-ship or co-authorship	Research projects/ clinical studies	Ownership interests (patent, copyright, shares)	Indirect interests	Guideline topics affected by COI<br>Degree of relevance<br>Consequence
							and deactivation of macrophages, NK cells, innate lymphoid cells, nitric oxide synthase, arginase 1); experi-mental cutaneous and visceral leish-maniasis; immunological effects and side effects of COVID-19<br>Clinical activities: clinical-microbio-logical testing and treatment of non-viral infectious agents; vaccinations (incl. COVID-19); epidemiology, diagnostic testing and treatment of cutaneous and visceral leishmaniasis; Whipple's disease<br>Involvement in further education/ training: co-organiser of a micro-biology, virology and infectiology training series at University Hospital Erlangen	
Dr jur. Breinlinger, Astrid	No	No	No	No	No	No	Memberships: chair of the Association for Borreliosis and TBE in Germany	None<br>None<br>None
Dahlem, Ursula	No	No	No	No	No	No	Memberships: OnLyme-Aktion.org Action Alliance Against Tick-Borne Infections Germany (patient associ-ation), representative and chair of OnLyme-Aktion.org	None<br>None<br>None
Dr Dersch, Rick	Pfizer	No	Roche, Sanofi, Novartis, Bayer, Argenx, Alexion, Merck	Thieme Publishing House; Hans-Christoph Diener	No	No	Memberships: DGN, German Society for Cerebrospinal Fluid Diagnostics and Neurochemistry, German Society for Clinical Neuro-physiology and Functional Imaging<br>Scientific activities: Lyme neuroborreliosis, neuroinfectiology, neuroimmunology, evidence-based medicine, cerebrospinal fluid testing<br>Clinical activities: neuroimmunology/ neuro-infectiology, cerebrospinal fluid testing	Member of the Data Adjudication Committee for Lyme borreliosis vaccinations. Vaccination not currently covered by the guideline<br>None<br>No voting rights planned
Dr med. Fingerle, Volker	QCMD (quality control for molecular diagnostics)	Pfizer	Bavarian State Office for Health and Food Safety, Academy for Health and Food Safety – AGL,	No	National Reference Centre for Borrelia, Bavarian Health and Food Safety Authority	No	Memberships: Instand, EQA expert for Borrelia PCR and serology, steering committee of ESGBOR (ESCMID study group for Lyme borreliosis), DGHM<br>Scientific activities: head of the	None<br><br>Brief member of an advisory board on vaccine develop-pment for a pharmaceutical

	Activity as a consultant and/ or expert	Participation in a scientific advisory board	Paid lecturing or training activity	Paid authorship or co-authorship	Research projects/ clinical studies	Ownership interests (patent, copyright, shares)	Indirect interests	Guideline topics affected by COI<br>Degree of relevance<br>Consequence
			med update GmbH, Infektio Update 2020 Wiesbaden, Young DGHM/ Organising team of a medical specialist revision course, medical supply centre Dr Fenner + Colleagues, German Society for Tropical Medicine, Travel Medicine and International Health, medical specialist revision course on microbiology, virology and infection epidemiology, 2022 symposium in Überlingen; medical supply centre of laboratory physicians Singen, 23rd annual meeting of the working group Dermatological Infectiology and Tropical Dermatology, scientific meeting<br>4th Labuda's Days, 32nd ECCMID, Lisbon, Academy for Infection Medicine, QCMD: international advisory board meeting in 2023				German National Reference Centre for Borrelia, various publications on Borrelia, Lyme Borreliosis, SARS-CoV-2 etc. e.g.: Borrelia Ecology and Evolution: Ticks and Hosts and the Environment, Epidemiological Surveillance of Lyme Borreliosis in Bavaria, Incidence of notified Lyme borreliosis in Germany, Guidelines for diagnosis and treatment in neurology – Lyme neuroborreliosis, Controversies in bacterial taxonomy: The example of the genus Borrelia, Tick-borne Diseases, Borreliosis, Lyme Borreliosis, Characteristics of Borrelia burgdorferi sensu lato, Laboratory Diagnosis of Lyme Borreliosis, Bavarian SARS-CoV-2-Public Health Laboratory Team. Comparison of nine different commercially available molecular assays for detection of SARS-CoV-2 RNA, Detection of the new SARS-CoV-2 variants of concern, SARS-CoV-2 Sentinel Surveillance in Primary Schools, Kindergartens, and Nurseries; and many more…<br><br>Involvement in further education/training: Virtual ESGBOR – ICLB symposium | International Conference on Lyme Borreliosis and other Tick-borne diseases (iclb2022.org). Lead organiser of Tick Webinar, 17 November 2021	company. As there is currently no vaccine approved for use in humans, this is not a topic of the guideline<br>None

	Activity as a consultant and/ or expert	Participation in a scientific advisory board	Paid lecturing or training activity	Paid author-ship or co-authorship	Research projects/ clinical studies	Ownership interests (patent, copyright, shares)	Indirect interests	Guideline topics affected by COI<br>Degree of relevance<br>Consequence
Prof. Dr med. Freitag, Michael	No	DAK-Gesundheit health insurance	No	No	Innofonds Project HOMERN and Projekt KOPAL	No	Memberships: DEGAM (general medicine), guideline work at SLK (Lyme borreliosis, asthma/COPD, irritable bowel syndrome)<br>Scientific activities: Lyme borreliosis, nursing home residents, emergency room, on-call service, antibiotics, prostate cancer screening, gout<br>Clinical activities: general medicine	None<br>None<br>None
PD D med. Gossrau, Gudrun	Social court, Saxony police	Novartis, Lilly, Teva, Lundbeck	Saxon State Medical Association, Novartis, Lilly, Teva, no	Teva	Novartis	No	Memberships: member of the executive committee of the German Migraine and Headache Society<br>Scientific activities: member of the Paediatric Pain Working Group at the German Pain Society<br>Clinical activities: member of the International Headache Society<br>Involvement in further education/ training: member of the DGN	None<br>None<br>None
Heidelmann, Georg	No	No	No	No	No	No	Memberships: patient organisation at the Association for Borreliosis and TBE in Germany,	None<br>None<br>None
Prof. Dr med. Hausteiner-Wiehle, Constanze	DGUV	No	CARUS Qualification Programme for Clinical Research, Psychosomatic Clinic Windach	Deutsche Medizinische Wochen-schrift, Deutsches Ärzteblatt, various specialist book publishers (Schattauer/ Klett-Cotta, Elsevier), VG Wort	No	No	Memberships: German Congress of Psychosomatic Medicine DKPM and German Society for Psychosomatic Medicine and Medical Psychotherapy DGPM, steering group for the AWMF guideline “Functional Body Complaints” (with patient guideline), author of AWMF guideline “Cutaneous Lyme Borreliosis”; member and board of the Functional Neurological Disorders WG, Journal of Psychosomatic Research (editorial board)<br>Scientific and clinical activities: consultation and liaison psychosomatics, functional physical complaints	None<br>None<br>None
Prof. Dr med. Hofmann, Heidelore	Infecto-pharm, expert opinions for arbitration board of the medical associations	No	No	No	No	No	Memberships: German Dermatology Society, Dermatological Infectiology WG, Paediatric Dermatology WG<br>Clinical activities: Vaccinating physician for SARS-CoV-2 vaccination	Contract fees from the pharmaceutical industry on a guideline topic<br>Low<br><br>None

	Activity as a consultant and/ or expert	Participation in a scientific advisory board	Paid lecturing or training activity	Paid author-ship or co-authorship	Research projects/ clinical studies	Ownership interests (patent, copyright, shares)	Indirect interests	Guideline topics affected by COI<br>Degree of relevance<br>Consequence
Prof. Dr med. Hunfeld, Klaus-Peter	Roche, Diasorin	Instand	No	Springer Publishing House	No	No	Memberships: DGHM, DGKL, PEG, BÄMI, Instand<br>Scientific activities: vector-borne pathogens, public health, sepsis, microbiology testing<br>Clinical activities: laboratory medicine and microbiology, hygiene in hospitals	None<br>Consultant/expert for the pharmaceutical industry: specific/commercial tests, including Lyme borreliosis testing, are neither discussed nor evaluated/recommended in the guideline: therefore, no limitations<br>None
Prof. Dr med. Huppertz, Hans-Iko	No	Pfizer, GSK, Biontech	No	No	No	No	Memberships: German Academy of Child and Adolescent Health, Alliance for Child and Adolescent Health, representative for children’s rights<br>Scientific activities: paediatric infectiology, rheumatology, immunology<br>Clinical activities: general paediatrics, rheumatology, immunology, infectiology	None<br>None<br>None
PD Dr med. Kastenbauer, Stefan	No	No	No	No	No	No	Scientific activities: neuroinfectiology<br>Clinical activities:<br>neurology practice	None<br>None<br>None
Prof. Dr Kopp, Ina	German Accreditation Body (DAkkS)	Institute for Quality Assurance and Transparency in Healthcare (IQTIG), German Agency for Quality in Medicine (ÄZQ)	EBM Frankfurt, WG of the Institute of General Medicine in the Faculty of Medicine at Johann Goethe University Frankfurt, European Federation of Periodontology, European Society of Endotontology (ESE), German Society for Paediatric Infectious Diseases (DGPI)/ German Academy for Child	VG-Wort	German Cancer Aid (DKH), Federal Ministry of Health (BMG), the Federal Joint Committee (G-BA),<br>Innovation Fund, Federal Ministry of Education and Research (BMBF)	None	Memberships: Steering committee for the oncology guideline programme of the German Cancer Society, German Cancer Aid and AWMF, Standing Commission on Guidelines of the AWMF (deputy chair), primary contact on behalf of the AWMF in the Guidelines International Network, German Network for Evidence-based Medicine, German Society of Surgery, Advisory Board for National Healthcare Guideline Programme of the German Medical Association, National Association of Statutory Health Insurance Physicians and AWMF, Cohort 1: SCIANA-Health Leaders Network, funded by the Robert Bosch Foundation, Health Foundation, Careum Foundation, board of trustees of the Institute for	None<br>None<br>None

	Activity as a consultant and/ or expert	Participation in a scientific advisory board	Paid lecturing or training activity	Paid author-ship or co-authorship	Research projects/ clinical studies	Ownership interests (patent, copyright, shares)	Indirect interests	Guideline topics affected by COI<br>Degree of relevance<br>Consequence
			Development and Health, European Business School (EBS) of the University for Business and Law gGmbH, British Society for Periodon-tology (BSP), European Society for Con-tact Dermatitis (ESCD), Euro-pean Association of Dental Implan-tologists (BDIZ)				Quality Assurance and Transparency in Healthcare (IQTIG), German Society for Senology<br>Scientific activities: guidelines, quality management, healthcare research<br>Involvement in further education/training: seminars AWMF guidelines for guideline developers and the curriculum for guideline advisors, methods workshops for the oncology guideline programme	
Prof. Dr. Krause, Andreas	BMS, Valneva/Pfizer	AbbVie, Amgen BMS, Boehringer Ingelheim, Gilead Janssen, Lilly, MSD,<br>Mylan, Novartis, Pfizer, Sanofi	AbbVie, Amgen, Berlin Chemie, BMS,<br>Boehringer Ingelheim,<br>Gilead, Janssen, Lilly, Medac, MSD,<br>Novartis, Pfizer, Roche,<br>Sanofi, UCB	Boehringer Ingelheim	AbbVie, Lilly, Novartis,	None	Memberships: board of the German Society for Rheumatology and Clinical Immunology, board of the Association of Acute Rheumatology Clinics, Professional Association of German Rheumatologists, Deutsche Rheumaliga, German Society of Internal Medicine<br>Scientific activities: pulmonary involvement in rheumatic diseases, healthcare<br>Clinical activities: internal medicine rheumatology	None<br>Member of an advisory board for the pharmaceutical industry on a topic not directly related to the guideline (vaccination), therefore irrelevant<br>None
Prof. Dr Mehling, Matthias	Merck, Roche, Novartis	No	University of Basel, Canton of St. Gallen Hospital, Swiss Multiple Sclerosis Society, Biogen, Merck, Roche, Biogen	No	Swiss National Science Foundation, Roche, Merck	University of Würzburg	Memberships: Medical and Scientific Advisory Board of the Swiss Multiple Sclerosis Society, DRG commission of the Swiss Neurological Society<br>Scientific activities: immune response in MS patients receiving immunotherapy, role of atypical chemokine receptors in multiple sclerosis, role of cytokine GDF-15 in multiple sclerosis<br>Clinical activities: clinical care of patients in the general neurological ward at the University Hospital of Basel<br>Involvement in further education/ training: training officer in the	None<br><br>Advisor/expert for the pharmaceutical industry on a topic not directly related to the guideline, therefore irrelevant<br>None

	Activity as a consultant and/ or expert	Participation in a scientific advisory board	Paid lecturing or training activity	Paid author-ship or co-authorship	Research projects/ clinical studies	Ownership interests (patent, copyright, shares)	Indirect interests	Guideline topics affected by COI<br>Degree of relevance<br>Consequence
							Neurological Clinic of the University Hospital of Basel, coordination and participation in the structured training programme	
Prof. Dr med. Müller, Rainer	No	No	No	No	No	No	Memberships: DGHNO, DGPP<br>Clinical activities: ENT consultant, phoniatrics and paedaudiology consultations	None<br>None<br>None
PD Dr Pfausler, Bettina	No	No	Talk on complex CNS infections	No	No	No	Memberships: Austrian Society of Neurology<br>Scientific and clinical activities: neuroinfection, neurointensive medicine	None<br>None<br>None
Prof. Dr Pfister, Hans-Walter	No	No	No	No	No	No	Memberships: DGN<br>Scientific activities: neuroinfectiology<br>Clinical activities: neuroinfectiology, neurointensive medicine	None<br>None<br>None
Prof. Dr med. Rauer, Sebastian	Roche Pharma AG, Novartis Pharma GmbH, Bristol-Myers Squibb GmbH, Hexal AG, Valneva Austria GmbH, Pfizer USA	Roche Pharma AG, Novartis Pharma GmbH, Bristol-Myers Squibb GmbH, Hexal AG, Valneva Austria GmbH, Pfizer USA	Roche Pharma AG, Novartis Pharma GmbH, Bristol-Myers Squibb GmbH, Biogen GmbH, Merck Healthcare Germany GmbH, Sanofi Aventis Deutschland GmbH	Thieme Publishing House, Stuttgart	Novartis Pharma GmbH, Roche Pharma AG, Biogen GmbH, Clinvices	Co-owner (50%) of the company ravo Diagnostika GmbH Freiburg	Memberships: DGN<br>Scientific activities: German Society for CSF Diagnostics and Clinical Neurochemistry (DGLN) c/o Kornelia Hauser<br>Clinic for Neurology at University Hospital Ulm, neuroimmunology, neuroinfectiology, general neurology	Co-owner of a company that manufactures serology tests for diagnosing Lyme borreliosis<br>High (testing)<br>No deliberations, no voting rights<br>Remark: Mr. Kastenbauer is co-coordinator without topically relevant conflicts of interest in the steering group
Prof. Rieger, Monika A.	No	No	No	No	None, funded by BMG, funded by the Ministry of Science, Research and Arts of Baden-Wuerttemberg	No	Memberships: guideline officer for the board of DGAUM, DGAUM representative for the Cutaneous Lyme Borreliosis Guideline and the Lyme Neuroborreliosis Guideline, DGAUM representative in the general meeting of the German Network for Healthcare Research<br>Scientific activities: occupational healthcare research and use of physiological methods to understand work-related stress and design good working practices<br>Clinical activities: company physician for various companies and	None<br>None<br>None

	Activity as a consultant and/ or expert	Participation in a scientific advisory board	Paid lecturing or training activity	Paid author-ship or co-authorship	Research projects/ clinical studies	Ownership interests (patent, copyright, shares)	Indirect interests	Guideline topics affected by COI<br>Degree of relevance<br>Consequence
							institutions, particularly the University of Tübingen<br>Personal relationship: brother is a lawyer at Allianz Private Health Insurance	
Dr med. Dr med. dent. Rixecker, Herbert	No	No	No	No	No	No	Memberships: German Society for Oral and Maxillofacial Surgery, German Society for Implantology, German Society for Dentistry, Oral and Maxillofacial Surgery, member and first chair of the German Lyme Disease Association<br>Clinical activities:<br>oral and maxillofacial surgery	None<br>None<br>None
Prof. Dr med. Salzberger, Bernd	No	No	No	No	No	No	Memberships: first chair/board of the German Society for Infectiology<br>Scientific activities: viral infections (HIV, CMV, Influenza, SARS-CoV-2)<br>Clinical activities: treatment of patients with infections, including Lyme borreliosis	None<br>None<br>None
Prof. Dr Sturzenegger, Mathias	No	No	No	No	No	No	Memberships: vice president of the Swiss Parkinson's Association, president of the awards committee of the Annemarie Opprecht Foundation for Parkinson's research	None<br>None<br>None
Adjunct Prof. Dr med. Tesarz, Jonas	No	EMDRIA	German Society for Pain Psycho-therapy, German Pain Society	Neuro-Aktuell, KlettCotta Publishing House	Federal Ministry of Education and Research, German Research Foundation	No	Memberships: German Society for Psychosomatic Medicine and Medical Psychotherapy (DGPM)<br>Scientific activities: German Congress of Psychosomatic Medicine (DKPM)<br>Clinical activities: German Pain Society (DGSS)<br>Involvement in further education/training: International Association for the Study of Pain (IASP)<br><br>Personal relationship: EMDR professional association (EMDRIA)	None<br>None<br>None

	Activity as a consultant and/ or expert	Participation in a scientific advisory board	Paid lecturing or training activity	Paid author-ship or co-authorship	Research projects/ clinical studies	Ownership interests (patent, copyright, shares)	Indirect interests	Guideline topics affected by COI<br>Degree of relevance<br>Consequence
Prof. Dr Thurau, Stephan	No	Alimera, Allergan, Kiora/Panoptes, Vienna	Alimera, AbbVie, Allergan, Novartis, Kiora/ Panoptes, Wien,<br>KWHC GmbH, MedKom-Akademie, Forum für-medizinische-Fortbildung-FomFGmbH, Takeda	Benemed	Kiora/Panoptes, Vienna	No	Memberships: German Ophthal-mological Society, Association for Research in Vision and Ophthal-mology,<br>Scientific activities: Uveitis – cures, new active substances<br>Clinical activities: diagnosis and treatment of ocular inflammation for all age groups, with a particular focus on intraocular inflammation (uveitis), retinopathy in premature infants	None<br>None<br>None
Prof. Dr rer.nat. Wallich, Reiner	No	No	No	No	No	No	No	None<br>None<br>None
Dr med. Wilking, Hendrik	Federal Ministry of Health	No	No	No	No	No	Scientific activities: infection epidemiology, public health<br>Involvement in further education/ training: head of instruction for infectious disease epidemiology at Charité Berlin	None<br>None<br>None
da Silva, Marianna	No	No	No	No	No	No	No	None<br>None<br>None

Appendix 2: Consensus Meeting for the S3 Guideline on Lyme Neuroborreliosis

18 July 2023, 1:00 pm to 7:00 pm, ZOOM

**Representative**	**Medical society/organisation**	**Present**
Prof. Sebastian Rauer, coordinator	German Society of Neurology (DGN), no voting rights	Yes
PD Dr Stephan Kastenbauer, coordinator	German Society of Neurology (DGN)	Yes
PD Dr Rick Dersch, evidence process	No voting rights	Yes
Prof. Dr med. Heidelore Hofmann	German Dermatology Society (DDG)	Yes
Dr med. Volker Fingerle	German Society for Hygiene and Microbiology (DGHM)	Yes
Prof. Dr med. Hans-Iko Huppertz	German Society of Paediatrics and Adolescent Medicine (DGKJ)<br>German Society for Paediatric Infectious Diseases (DGPI)	Yes
Prof. Dr med. Klaus-Peter Huhnfeld	The German Society for Clinical Chemistry and Laboratory Medicine (DGKL)<br>INSTAND	Yes
Prof. Dr med. Andreas Krause	German Society for Rheumatology and Clinical Immunology (DGRh)	Excused
Prof. Dr med. Bernd Salzberger	German Society for Infectious Diseases (DGI)	Excused
Prof. Dr med. Karl Bechter	German Association for Psychiatry, Psychotherapy and Psychosomatics (DGPPN)	Yes
Dr med. Herbert Rixecker	German Borreliosis Society (DBG)	Yes
Ursula Dahlem	Action Alliance Against Tick-Borne Infections Germany (OnLyme-Aktion)	Yes
Dr Astrid Breinlinger	Association for Borreliosis and TBE in Germany	Yes
Prof. Dr med. Michael H. Freitag	German Society of General Practice/Family Medicine (DEGAM)	Yes
PD Dr med. Gudrun Gossrau	German Pain Society (DGSS)	Yes
Prof. Dr med. Christian Bogdan	Paul Ehrlich Society for Infection Therapy (PEG)	Yes
Prof. Dr med. Monika A. Rieger	German Society for Occupational Medicine and Environmental Medicine (DGAUM)	Yes
Prof. Dr med. Constanze Hausteiner-Wiehle	German Society for Psychosomatic Medicine and Medical Psychotherapy (DGPM)	Yes
Prof. (adjunct) Dr med. Jonas Tesarz	German Congress of Psychosomatic Medicine (DKPM)	Yes
Prof. Dr med. Stephan Thurau	German Society of Ophthalmology (DOG)	Yes
Prof. Dr rer. Nat. Reinhard Wallich	German Society for Immunology (DGfI)	Yes
Dr med. vet. Hendrik Wilking	Robert Koch Institute (RKI)	Excused
PD. Dr med. Bettina Pfausler	Austrian Society of Neurology (ÖGN)	Excused
Prof. Dr med. Klaus Sturzenegger	Swiss Neurological Society (SNG)	Excused
Marianna da Silva	Young Neurology (no voting rights)	Excused
Prof. Dr med. Ina. B. Kopp	AWMF Institute for Medical Knowledge Management, moderator	Yes

Appendix 3: Consensus Meeting for the S3 Guideline on Lyme Neuroborreliosis

28/11/2023, 1:00 pm – 7:00 pm, ZOOM

Mandate holder	Medical society/organisation	Present
Prof. Sebastian Rauer, coordinator	German Society of Neurology (DGN), no voting rights	Yes
PD Dr Stephan Kastenbauer, coordinator	German Society of Neurology (DGN)	Yes
PD Dr Rick Dersch, evidence process	No voting rights	Yes
Prof. Dr med. Heidelore Hofmann	German Dermatology Society (DDG)	Yes
Dr med. Volker Fingerle (2 votes)	German Society for Hygiene and Microbiology (DGHM)	Yes
Prof. Dr med. Hans-Iko Huppertz	German Society of Paediatrics and Adolescent Medicine (DGKJ)<br>German Society for Paediatric Infectious Diseases (DGPI)	Excused
Prof. Dr med. Klaus-Peter Hunfeld (represented by: H. Fingerle)	The German Society for Clinical Chemistry and Laboratory Medicine (DGKL)<br>INSTAND	Excused (represented by H. Fingerle)
Prof. Dr med. Andreas Krause	German Society for Rheumatology and Clinical Immunology (DGRh)	Yes until 3:30 pm, voting rights transferred to H. Kastenbauer
Prof. Dr med. Bernd Salzberger	German Society for Infectious Diseases (DGI)	Yes
Prof. Dr med. Karl Bechter	German Association for Psychiatry, Psychotherapy and Psychosomatics (DGPPN)	Yes
Dr med. Herbert Rixecker (represented by Dr Bennefeld)	German Borreliosis Society (DBG)	Yes
Ursula Dahlem	Action Alliance Against Tick-Borne Infections Germany (OnLyme-Aktion)	Yes
Dr Astrid Breinlinger (stepped down, new representative: G. Heidelmann)	Association for Borreliosis and TBE in Germany	Yes
Prof. Dr med. Michael H. Freitag	German Society of General Practice/Family Medicine (DEGAM)	Yes
PD Dr med. Gudrun Gossrau	German Pain Society (DGSS)	Yes
Prof. Dr med. Christian Bogdan	Paul Ehrlich Society for Infection Therapy (PEG)	Yes
Prof. Dr med. Monika A. Rieger	German Society for Occupational Medicine and Environmental Medicine (DGAUM)	Excused
Prof. Dr med. Constanze Hausteiner-Wiehle	German Society for Psychosomatic Medicine and Medical Psychotherapy (DGPM)	Excused
Prof. (adjunct) Dr med. Jonas Tesarz	German Congress of Psychosomatic Medicine (DKPM)	Yes
Prof. Dr med. Stephan Thurau	German Society of Ophthalmology (DOG)	Yes
Prof. Dr rer. Nat. Reinhard Wallich	German Society for Immunology (DGfI)	Yes
Dr med. vet. Hendrik Wilking	Robert Koch Institute (RKI)	Excused
PD. Dr med. Bettina Pfausler	Austrian Society of Neurology (ÖGN)	Yes
Prof. Dr med. Matthias Sturzenegger	Swiss Neurological Society (SNG)	Yes
Marianna da Silva	Young Neurology (no voting rights)	No
Prof. Dr med. Ina. B. Kopp	AWMF Institute for Medical Knowledge Management, moderator	Yes

Appendix 4: Dissenting opinion, OnLyme

Dissenting opinion to the Lyme Neuroborreliosis Guideline

OnLyme-Aktion.org
Aktionsbündnis gegen zeckenübertragene Infektionen Deutschland e. V.

c/oUrsula Dahlem
Am Haag 21
65812 Bad Soden
E-Mail: vorstand@onlyme-aktion.org
Fax: +49 61969504840
www.Onlyme-Aktion.org

The originally planned AWMF S3 guideline on Lyme borreliosis, which was to cover all manifestations of Lyme borreliosis, has been withdrawn by the relevant medical associations. However, this was a fundamental basis for our cooperation. As the patients we represent are not sufficiently considered in the two resulting guidelines, our General Assembly voted on 4 May 2024 not to approve this version of the guideline.

Even though the guideline on Lyme neuroborreliosis represents an important pillar alongside the Guideline on Cutaneous Manifestations of Lyme Borreliosis and can counteract the lack of awareness by physicians for Lyme borreliosis, the proportion of clearly defined neuroborreliosis diseases presented here is small in the overall context of Lyme borreliosis.

If Lyme borreliosis is diagnosed too late or is insufficiently treated, patients run the risk of developing chronic conditions that easily fall outside the scope of this guideline. There are still large diagnostic gaps in the early detection and treatment of Lyme borreliosis, which are not addressed by this guideline. Current test methods fall short in determining with sufficient certainty the disease activity of Lyme borreliosis. Lyme neuroborreliosis cannot always be diagnosed with certainty in the early stages; in the case of a reinfection, an overlapping of old and newly produced antibodies can blur the results and lead to misinterpretations.

We refuse to be collateral damage because the treatment for confirmed cases of Lyme neuroborreliosis is only sufficiently effective in 85–90% of the cases. Targeted therapies cannot be derived from the new efforts relating to the results of the systemic review on persistent symptoms after antibiotic treatment (“post-treatment Lyme disease syndromes”, PTLDS for short). The discrepancy cannot be resolved by referring to other guidelines, especially as treating these mostly complex clinical symptoms is becoming increasingly difficult given the current shortage of GPs and specialists. This dilemma is in line with the experience of many patients.

We are well aware that patient experience alone, which is not supported by research findings due to a lack of studies, cannot fulfil the scientific requirements of an S2k or S3 guideline; however, even after 10 years in which we have been intensively involved in guideline activities, there has been no scientific progress in Germany or even a substantiation of the need for research that can contribute to improving the treatment situation for the patients we represent. We see this as the task of the scientific societies and attending physicians – not as that of the patients.

Patients with persistent symptoms following antibiotic treatment continue to receive inadequate support during their often years-long ordeal. This can have existential consequences. Unfortunately, we do not believe that this guideline provides the necessary steps towards improving care. Simply referring to differential diagnoses and the treatment of symptoms is becoming increasingly difficult due to the lack of points of contact for patients. Unfortunately, we are not aware of any research results or practical examples that prove that this approach is actually successful.

Our objections to the important points on PTLDS, the corresponding need for research, the shortcomings of testing, and the inadequate care of patients with persistent symptoms are only partially addressed by the guideline but are not resolved. At our Annual General Meeting on 4 May 2024, a large majority of our members felt that this guideline did not improve the “current situation” as much as those affected would require. With this dissenting opinion, OnLyme-Action.org expresses its intention not to support the final version of the guideline.

However, we would like to signal our continued willingness to cooperate. We hope for urgently needed improvements in the care of affected patients and would also very much welcome new research efforts in Germany.

Bad Soden, 12 May 2024

Ursula Dahlem

First chair

Appendix 5: Comments by the BFBD

A) According to Section 2(2) of the resolution by the GBA to the Long Covid Guideline, https://www.g-ba.de/themen/qualitaetssicherung/beratungen-versorgungsangebot-long-covid/, the guideline can also pertain to patients with PTLDS or persistent symptoms of Lyme borreliosis or Lyme neuroborreliosis.

The guideline also covers suspected or diagnosed diseases that have a similar cause or manifestation like long COVID. In this sense, the guideline also covers patients of all age groups who

1. have post-acute symptoms similar to long COVID as a result of an infection or

2. have ME/CFS.

(3) The diagnosis is made based on the guideline or in accordance with the current state of medical and scientific knowledge on the basis of a symptom-orientated differential diagnosis or as *a diagnosis by exclusion.*

The differential diagnosis and in particular the diagnosis by exclusion should not hinder the prompt start of treatment if there is sufficient probability that disease is present in accordance with Section 2....

This guideline went into effect in April 2024.
https://www.bundesgesundheitsministerium.de/ministerium/meldungen/3-runder-tisch-long-covid

Accordingly, patients with disseminated Lyme borreliosis or symptoms of a persistent disease following guideline-compliant Lyme borreliosis treatment (PTLDS) can also be included, regardless of whether Lyme borreliosis can still be serologically detected.

B) Recent research findings indicate that borrelia can survive in the cell and exist in morphologically different forms or can trigger autoimmune reactions. However, further research is needed to clarify the mechanisms involved. As a patient association, we are calling for further research.

We refer, for example, to the following articles:

- Karvonen K, Tammisto H, Nykky J, Gilbert L. *Borrelia burgdorferi* Outer Membrane Vesicles Contain Antigenic Proteins, but Do Not Induce Cell Death in Human Cells. Microorganisms. 2022 Jan 19;10(2):212. doi: 10.3390/microorganisms10020212.
- Karvonen K, Nykky J, Marjomäki V, Gilbert L. Distinctive Evasion Mechanisms to Allow Persistence of *Borrelia burgdorferi* in Different Human Cell Lines. Front Microbiol. 2021 Oct 12;12:711291. doi: 10.3389/fmicb.2021.711291. PMID: 34712208; PMCID: PMC8546339.

The decision to try exploratory antibiotics or alternative forms of treatment should be carefully weighed up by the physician. In our experience, there are always patients who can benefit from antibiotic treatment even in the advanced or late stages of the disease, including off-label use of antibiotics.

Furthermore, Lyme borreliosis is mostly diagnosed clinically, as the ELISA or Western blot test can be positive or negative depending on the test set used by the laboratory.

Patients with a negative or even positive serology and manifested symptoms without EM are repeatedly stigmatised as having psychosomatic symptoms, even though they have Lyme borreliosis or are suffering from a co-infection or another infection (bacteria, viruses or worms). The guideline also still lacks the acknowledgement of serology tests for borreliosis disease activity. We call for improved diagnosis through exclusion of other infections and a broader awareness of the symptoms of co-infections, especially among neurologists and general practitioners.

Sincerely,
Georg Heidelmann

BFBD representative on the guideline committee

BORRELIOSE UND FSME BUND DEUTSCHLAND
PATIENTENORGANISATION BUNDESVERBAND

Literature

1. Mygland A, Ljøstad U, Fingerle V, Rupprecht T, Schmutzhard E, Steiner I; European Federation of Neurological Societies. EFNS guidelines on the diagnosis and management of European Lyme neuroborreliosis. Eur J Neurol. 2010 Jan;17(1):8-16, e1-4. DOI: 10.1111/j.1468-1331.2009.02862.x
2. Kaiser R. Neuroborreliosis. J Neurol. 1998 May;245(5):247-55. DOI: 10.1007/s004150050214
3. Halperin JJ, Shapiro ED, Logigian E, Belman AL, Dotevall L, Wormser GP, Krupp L, Gronseth G, Bever CT Jr; Quality Standards Subcommittee of the American Academy of Neurology. Practice parameter: treatment of nervous system Lyme disease (an evidence-based review): report of the Quality Standards Subcommittee of the American Academy of Neurology [RETIRED]. Neurology. 2007 Jul 3;69(1):91-102. DOI: 10.1212/01.wnl.0000265517.66976.28
4. Rauer S, Kaiser R, Kölmel HW, Pfister HW, Schmutzhard E, Sturzenegger M, et al. Neuroborreliose. In: Diener HC, Weimar C, editors. Leitlinien für Diagnostik und Therapie in der Neurologie. Stuttgart, New York: Georg Thieme Verlag; 2012. p. 513-22.
5. Stanek G, Fingerle V, Hunfeld KP, Jaulhac B, Kaiser R, Krause A, Kristoferitsch W, O'Connell S, Ornstein K, Strle F, Gray J. Lyme borreliosis: clinical case definitions for diagnosis and management in Europe. Clin Microbiol Infect. 2011 Jan;17(1):69-79. DOI: 10.1111/j.1469-0691.2010.03175.x
6. Brouwers MC, Ako-Arrey D, Spithoff K, Vukmirovic M, Florez ID, Lavis JN, Cluzeau F, Permanand G, Bosch-Capblanch X, Chen Y; AGREE-HS Research Team. Validity and usability testing of a health systems guidance appraisal tool, the AGREE-HS. Health Res Policy Syst. 2018 Jun 20;16(1):51. DOI: 10.1186/s12961-018-0334-9
7. Eldin C, Raffetin A, Bouiller K, Hansmann Y, Roblot F, Raoult D, Parola P. Review of European and American guidelines for the diagnosis of Lyme borreliosis. Med Mal Infect. 2019 Mar;49(2):121-32. DOI: 10.1016/j.medmal.2018.11.011
8. Eikeland R, Henningsson AJ, Lebech AM, Kerlefsen Y, Mavin S, Vrijlandt A, Hovius JW, Lernout T, Lim C, Dobler G, Fingerle V, Gynthersen RM, Lindgren PE, Reiso H. Tick-borne diseases in the North Sea region-A comprehensive overview and recommendations for diagnostics and treatment. Ticks Tick Borne Dis. 2024 Mar;15(2):102306. DOI: 10.1016/j.ttbdis.2023.102306
9. Solheim AM, Lorentzen ÅR, Dahlberg AO, Flemmen HØ, Brune S, Forselv KJN, Pripp AH, Bø MH, Eikeland R, Reiso H, Mygland Å, Ljøstad U. Six versus 2 weeks treatment with doxycycline in European Lyme neuroborreliosis: a multicentre, non-inferiority, double-blinded, randomised and placebo-controlled trial. J Neurol Neurosurg Psychiatry. 2022 Jul 27;93(11):1222–8. DOI: 10.1136/jnnp-2022-329724
10. Kortela E, Kanerva MJ, Puustinen J, Hurme S, Airas L, Lauhio A, Hohenthal U, Jalava-Karvinen P, Nieminen T, Finnilä T, Häggblom T, Pietikäinen A, Koivisto M, Vilhonen J, Marttila-Vaara M, Hytönen J, Oksi J. Oral Doxycycline Compared to Intravenous Ceftriaxone in the Treatment of Lyme Neuroborreliosis: A Multicenter, Equivalence, Randomized, Open-label Trial. Clin Infect Dis. 2021 Apr 26;72(8):1323-31. DOI: 10.1093/cid/ciaa217
11. Oteo JA, Corominas H, Escudero R, Fariñas-Guerrero F, García-Moncó JC, Goenaga MA, Guillén S, Mascaró JM, Portillo A. Executive summary of the consensus statement of the Spanish Society of Infectious Diseases and Clinical Microbiology (SEIMC), Spanish Society of Neurology (SEN), Spanish Society of Immunology (SEI), Spanish Society of Pediatric Infectology (SEIP), Spanish Society of Rheumatology (SER), and Spanish Academy of Dermatology and Venereology (AEDV), on the diagnosis, treatment and prevention of Lyme borreliosis. Enferm Infecc Microbiol Clin (Engl Ed). 2023 Jan;41(1):40-5. DOI: 10.1016/j.eimce.2022.11.011
12. Gocko X, Lenormand C, Lemogne C, Bouiller K, Gehanno JF, Rabaud C, Perrot S, Eldin C, de Broucker T, Roblot F, Toubiana J, Sellal F, Vuillemet F, Sordet C, Fantin B, Lina G, Sobas C, Jaulhac B, Figoni J, Chirouze C, Hansmann Y, Hentgen V, Caumes E, Dieudonné M, Picone O, Bodaghi B, Gangneux JP, Degeilh B, Partouche H, Saunier A, Sotto A, Raffetin A, Monsuez JJ, Michel C, Boulanger N, Cathebras P, Tattevin P; endorsed by the following scientific societies. Lyme borreliosis and other tick-borne diseases. Guidelines from the French scientific societies. Med Mal Infect. 2019 Aug;49(5):296-317. DOI: 10.1016/j.medmal.2019.05.006
13. Lantos PM, Rumbaugh J, Bockenstedt LK, Falck-Ytter YT, Aguero-Rosenfeld ME, Auwaerter PG, et al. Clinical Practice Guidelines by the Infectious Diseases Society of America (IDSA), American Academy of Neurology (AAN), and American College of Rheumatology (ACR): 2020 Guidelines for the Prevention, Diagnosis and Treatment of Lyme Disease. Clin Infect Dis. 2021;72(1):1-8.
14. Cruickshank M, O'Flynn N, Faust SN; Guideline Committee. Lyme disease: summary of NICE guidance. BMJ. 2018 Apr 12;361:k1261. DOI: 10.1136/bmj.k1261

15. Nemeth J, Bernasconi E, Heininger U, Abbas M, Nadal D, Strahm C, Erb S, Zimmerli S, Furrer H, Delaloye J, Kuntzer T, Altpeter E, Sturzenegger M, Weber R, For The Swiss Society For Infectious Diseases And The Swiss Society For Neurology. Update of the Swiss guidelines on post-treatment Lyme disease syndrome. Swiss Med Wkly. 2016 Dec 5;146:w14353. DOI: 10.4414/smw.2016.14353

16. Habegger S. Lyme Disease in Canada: An Update on Case Definitions and Treatments. In: Diseases NCCfI, editor. National Collaborating Centre for Infectious Diseases. 2014. p. 1-11.

17. Dersch R, Freitag MH, Schmidt S, Sommer H, Rauer S, Meerpohl JJ. Efficacy and safety of pharmacological treatments for acute Lyme neuroborreliosis - a systematic review. Eur J Neurol. 2015 Sep;22(9):1249-59. DOI: 10.1111/ene.12744

18. Dersch R, Rauer S. Efficacy and safety of pharmacological treatments for Lyme neuroborreliosis: An updated systematic review. Eur J Neurol. 2023 Dec;30(12):3780-8. DOI: 10.1111/ene.16034

19. Dersch R, Freitag MH, Schmidt S, Sommer H, Rücker G, Rauer S, Meerpohl JJ. Efficacy and safety of pharmacological treatments for neuroborreliosis--protocol for a systematic review. Syst Rev. 2014 Oct 21;3:117. DOI: 10.1186/2046-4053-3-117

20. Stupica D, Bajrović FF, Blagus R, Cerar Kišek T, Collinet-Adler S, Lah A, Levstek E, Ružić-Sabljić E. Clinical manifestations and long-term outcome of early Lyme neuroborreliosis according to the European Federation of Neurological Societies diagnostic criteria (definite versus possible) in central Europe. A retrospective cohort study. Eur J Neurol. 2021 Sep;28(9):3155-66. DOI: 10.1111/ene.14962

21. Marques A. Persistent Symptoms After Treatment of Lyme Disease. Infect Dis Clin North Am. 2022 Sep;36(3):621-38. DOI: 10.1016/j.idc.2022.04.004

22. Jowett N, Gaudin RA, Banks CA, Hadlock TA. Steroid use in Lyme disease-associated facial palsy is associated with worse long-term outcomes. Laryngoscope. 2017 Jun;127(6):1451-8. DOI: 10.1002/lary.26273

23. Arnason S, Skogman BH. Effectiveness of antibiotic treatment in children with Lyme neuroborreliosis - a retrospective study. BMC Pediatr. 2022 Jun 9;22(1):332. DOI: 10.1186/s12887-022-03335-w

24. Higgins JPT TJ, Chandler J, Cumpston M, Li T, Page MJ, Welch VA. Cochrane Hanbook for Systematic Reviews of Interventions. Edition 5.1.0. Cochrane; 2011.

25. Sterne JA, Hernán MA, Reeves BC, Savović J, Berkman ND, Viswanathan M, Henry D, Altman DG, Ansari MT, Boutron I, Carpenter JR, Chan AW, Churchill R, Deeks JJ, Hróbjartsson A, Kirkham J, Jüni P, Loke YK, Pigott TD, Ramsay CR, Regidor D, Rothstein HR, Sandhu L, Santaguida PL, Schünemann HJ, Shea B, Shrier I, Tugwell P, Turner L, Valentine JC, Waddington H, Waters E, Wells GA, Whiting PF, Higgins JP. ROBINS-I: a tool for assessing risk of bias in non-randomised studies of interventions. BMJ. 2016 Oct 12;355:i4919. DOI: 10.1136/bmj.i4919

26. Avellan S, Bremell D. Adjunctive Corticosteroids for Lyme Neuroborreliosis Peripheral Facial Palsy – A Prospective Study With Historical Controls. Clin Infect Dis. 2021 Oct 5;73(7):1211-5. DOI: 10.1093/cid/ciab370

27. Balshem H, Helfand M, Schünemann HJ, Oxman AD, Kunz R, Brozek J, Vist GE, Falck-Ytter Y, Meerpohl J, Norris S, Guyatt GH. GRADE guidelines: 3. Rating the quality of evidence. J Clin Epidemiol. 2011 Apr;64(4):401-6. DOI: 10.1016/j.jclinepi.2010.07.015

28. Dersch R, Hottenrott T, Schmidt S, Sommer H, Huppertz HI, Rauer S, Meerpohl JJ. Efficacy and safety of pharmacological treatments for Lyme neuroborreliosis in children: a systematic review. BMC Neurol. 2016 Sep 29;16(1):189. DOI: 10.1186/s12883-016-0708-y

29. Brown K, Corin S, Handel AS. Doxycycline for the Treatment of Lyme Disease in Young Children. Pediatr Infect Dis J. 2023 Dec 1;42(12):e470-e472. DOI: 10.1097/INF.0000000000004128

30. Dersch R, Toews I, Sommer H, Rauer S, Meerpohl JJ. Methodological quality of guidelines for management of Lyme neuroborreliosis. BMC Neurol. 2015 Nov 25;15:242. DOI: 10.1186/s12883-015-0501-3

31. Dersch R, Sommer H, Rauer S, Meerpohl JJ. Prevalence and spectrum of residual symptoms in Lyme neuroborreliosis after pharmacological treatment: a systematic review. J Neurol. 2016 Jan;263(1):17-24. DOI: 10.1007/s00415-015-7923-0
