# Supplementary material for: Guidelines for diagnosis and treatment in neurology – Lyme neuroborreliosis
Source: Ger Med Sci. 2025 Oct 9;23:Doc13. doi: 10.3205/000349 (PMC12584707; doi:10.3205/000349)
Supplement: Appendices [file GMS-23-13-s-002.pdf]

Attachment 2: Appendices

Appendix 1

Feder's criteria for presumptive "chronic Lyme borreliosis":

Feder et al. describe 4 clinical categories into which patients with presumptive "chronic Lyme borreliosis" can be assigned [164]:

1. Symptoms of an unknown origin without evidence of an infection with Borrelia burgdorferi. In this category, a combination of non-specific symptoms is assumed to be a strong indication of "chronic Lyme borreliosis" [280]. However, these non-specific symptoms are found in approximately 10% of the normal US population, regardless of whether the region is endemic for Lyme borreliosis [198], [199].
2. Symptoms of a known, well-defined disease without evidence of an infection with Borrelia burgdorferi. In this case, the original diagnosis is assumed to be a misdiagnosis (e.g. multiple sclerosis).
3. Symptoms of an unknown origin with a positive borrelia serology, but no objective clinical findings of Lyme borreliosis now or in the past.
4. Post-treatment Lyme disease syndrome (PTLDS) (see section 4.3 and Appendix 2).

Appendix 2

In 2006, the Infectious Diseases Society of America (IDSA) suggested the following diagnostic criteria for PTLDS [233]:

1. Previous, confirmed Lyme borreliosis (according to CDC criteria) and regression or stabilisation of objective symptoms of Lyme borreliosis under a generally accepted antibiotic treatment regimen.
2. Onset of subjective symptoms (fatigue, extensive musculoskeletal pain, cognitive disorders) within 6 months of Lyme borreliosis diagnosis and persistence of symptoms (or chronic recurrence) for at least 6 months after completion of antibiotic treatment.
3. The subjective symptoms lead to a relative impairment of daily activities.
4. Exclusion criteria:
    - Active, untreated co-infection
    - Objective findings during physical examination or neuropsychological testing that explain the symptoms
    - Subjective symptoms that were present before the Lyme borreliosis
    - Another underlying disease that explains the complaints (e.g. morbid obesity, sleep apnoea syndrome, narcolepsy, autoimmune diseases, adverse drug reactions, (insufficiently treated or decompensated) cardiopulmonary diseases, endocrine diseases, malignant diseases within the last 2 years, liver diseases, depressive disorders, bipolar disorders, delusional disorders, dementia, eating disorders, drug or alcohol abuse within the last 2 years)
    - Laboratory findings and/or imaging that could explain the symptoms (e.g. ESR > 50 mm/h, abnormal serum values for thyroid hormone, total protein, immunoglobulins, liver values, calcium, phosphorus, urea, electrolytes, creatinine; abnormal urine results)

Appendix 3

Table 1: Absolute results and manifestations taken from the 8 RCTs and 8 cohort studies (modified according to [16])

**Study (RCT)**	**Case definition**	**Manifestation**	**Residual neurological symptoms after 3–12 months**	
			**Doxycycline**	**Beta lactam antibiotics**
Kortela, 2021	97 confirmed (52%)<br>90 possible (48%)	170 early manifestations (91%)<br>17 late manifestations (9%)	35/82 (43%)	Ceftriaxone 36/84 (42%)
Solheim, 2022	76 confirmed (83%)<br>16 possible (17%)	89 early manifestations (97%)<br>3 late manifestations (>6 months) (3%)	2 weeks of treatment: 6/46 (13%)<br>6 weeks of treatment: 10/46 (22%)	
Ljostad, 2008	Confirmed (n=71)<br>(n=31)	Early manifestations (Bannwarth syndrome, cranial nerve paresis, radiculopathies)<br>n=97 (95%)<br>Late manifestations (myelopathy, ACA with paraesthesia, encephalopathy)<br>n=5 (5%)	28/54 (52%)	32/48 (66%) (ceftriaxone)
Karlsson, 1994	Probable	Not differentiable, predominantly early manifestations	6/31 (19%)	4/21 (19%) (penicillin G)
Kohlhepp, 1989	Possible	Early manifestations (radiculopathy, meningitis, cranial nerve paresis)	19/39 (49%)	23/36 (63%) (penicillin G)
**Study (RCT)**	**Case definition**	**Manifestations**	**Residual neurological symptoms after 12 months**	
			**Doxycycline**	**Beta lactam antibiotics**
Ljostad, 2008	Confirmed (n=71)<br>Possible (n=31)	See above	22/44 (50%)	19/41 (46%)
Karlsson, 1994	Probable	See above	3/30 (10%)	3/21 (14%)
Kohlhepp, 1989	Possible	See above	12/39 (30%)	11/36 (30%)
**Study (RCT)**	**Case definition**	**Manifestations**	**Residual neurological symptoms after >3 months**	
			**Cefotaxime**	**Penicillin G**
Hassler, 1990	Possible	Early manifestations (radiculopathies)	14/49 (28%)	24/44 (54%)
Pfister, 1989	3/21 possible<br>19/21 patient confirmed/probable	Early manifestations (Bannwarth syndrome, meningitis)	2/11 (18%)	2/10 (20%)
**RCT without relevant data for defined comparisons**				
**Study (RCT)**	**Case definition**	**Manifestations**		
Oksi, 1988	Possible	Not differentiable, predominantly early manifestations		
Pfister, 1988 (Pfister, HW 88)	3/21 possible<br>18/21 probable	Early manifestations (Bannwarth syndrome)		
Pfister, 1991	6/30 possible<br>24/30 confirmed/ probable<br>24/30 confirmed/ probable	Early manifestations (Bannwarth syndrome)		

Attachment 2 to: *Rauer S, Kastenbauer S, Dersch R, Hofmann H, Fingerle V, Huppertz HI, Hunfeld KP, Krause A, Salzberger B, Consensus group. Guidelines for diagnosis and treatment in neurology – Lyme neuroborreliosis. GMS Ger Med Sci. 2025;23:Doc13. DOI: 10.3205/000349, URN: urn:nbn:de:0183-0003498*

Appendix 4

Table 2: Frequency of side effects taken from 6 RCTs (modified according to [16])

**Beta lactams vs. doxycycline**					
**Design**	**Studies**	**Beta lactam group (n=)**	**Doxycycline group (n=)**	**Beta lactam side effects (n=)**	**Doxycycline side effects (n=)**
**RCTs**	2	79	88	32 (40%)	25 (28%)
**NRSs**	2	47	75	1 (2%)	5 (7%)
**Penicillin vs. cefotaxime**					
**Design**	**Studies**	**Cefotaxime group (n=)**	**Penicillin G group (n=)**	**Cefotaxime side effects (n=)**	**Penicillin G side effects (n=)**
**RCTs**	2	80	79	39 (49%)	22 (28%)

Appendix 5

Table 3: Side effects taken from comparison studies (modified according to [16])

**Individual side effects**		
**Beta lactams vs doxycycline**	**Beta lactams**	**Doxycycline**
**Kortela, 2021**	**n=93**	**n=94**
**All AE**	9	5
**AE/SAE**	Drug-induced exanthema, elevated liver enzymes, diarrhoea, Clostridium difficile infection, thrombophlebitis from intravenous access	Drug-induced exanthema, nausea, eczema after sun exposure
**Ljostad, 2008**	**n=56**	**n=57**
**All AE**	26	57
**SAE**	3 (cholecystitis/stomatitis/allergy)	1 (duodenal ulcer)
**Other (not broken down by treatment)**	Diarrhoea n=17, nausea n=3, nausea + diarrhoea n=2, constipation n=9, exanthema n=3	
**Karlsson, 1994**	**n=23**	**n=31**
**AE**	3 (dizziness n=1, thrombophlebitis n=2)	4 (exanthema n=2, diarrhoea n=2)
**Berglund, 2002**	**n=18**	**n=39**
**AE/SAE**	0	0
**Borg, 2005**	**n=29**	**n=36**
**AE**	1 (leucopoenia)	5 (GI issues n=3, phototoxicity n=2)
**Penicillin vs. cefotaxime**	**Penicillin**	**Cefotaxime**
**Hassler, 1990**	**n=69**	**n=69**
**AE**	20 (diarrhoea n=6, Herxheimer reaction n=14)	37 (diarrhoea n=9, Herxheimer reaction n=28)
**SAE**	n=2 (colitis, shock)	n=2 (colitis, allergic reaction)
**Pfister, 1989**	**n=10**	**n=11**
**AE**	0	0

Appendix 6

Patient information following a tick bite (taken from DDG-S2k-LL “Cutaneous Lyme Borreliosis”; AWMF-Reg.-Nr. 013/044 [51])

1. Remove the tick as quickly as possible.
2. The best tools are special tick tweezers or tick cards.
3. Slowly and patiently pull or push the tick out of the skin without twisting it or pre-treating it with oil or glue. Avoid squeezing the tick’s body. If part of the feeding apparatus remains in the skin (often misinterpreted as the “head”), it can be removed with a sterile needle or curette, or it can be removed by a physician. If the feeding apparatus remains in the skin, there is no danger that the Borrelia will be transferred.
4. Carefully examine your body and especially the heads of children for more ticks.
5. Observe the skin around the site of the bite for 6 weeks.
6. Any redness caused by the tick saliva, which appears immediately after the bite, will disappear within several days. If reddening reappears or if the initial reddening increases to ≥5 cm, a doctor should be consulted. This may be erythema migrans (migrating rash), which is an early manifestation of Lyme borreliosis.
7. If there is a typical migratory rash in the area around the tick bite, antibiotic treatment should be initiated, preferably with doxycycline (in children aged 9 and up) or with amoxicillin, even if no blood test has been carried out or if no antibodies are detectable yet in the blood.
8. The dissemination of the Borrelia through the blood – even without a reddening of the skin – is recognisable by a flu-like feeling without respiratory symptoms. They may be the precursor of organ disease, e.g. of the joints or nervous system. In this case, consult a physician who will decide whether a blood test for Borrelia antibodies is necessary.
9. Lyme borreliosis can be completely cured in the early stages with guideline-compliant antibiotic treatment, thus preventing late manifestations.
10. It is not advisable to test the tick for Borrelia, since a positive result does not mean that the Borrelia will have been transmitted to the skin or that they will cause an infection if they have been transmitted. A negative result does not rule out transmission.
11. Only a small proportion of people infected with Borrelia become ill! This is why prophylactic oral antibiotic treatment is not recommended.

Appendix 7

Table 4: Frequency of clinical manifestations related to Lyme neuroborreliosis taken from 3 studies (Kaiser, 1994 [14])

Clinical manifestation	Acute (early) Lyme neuroborreliosis n=86	Chronic (late) Lyme neuroborreliosis n=15
**Spinal radiculitis**<br>• Isolated spinal radiculitis<br>• Spinal and cranial radiculitis<br>• Isolated cranial radiculitis	73.2%<br>38.4%<br>34.9%<br>20.9%	
**Cranial radiculitis**<br>• VII<br>• II<br>• III<br>• VI	55.8%<br>51.2%<br>1.2%<br>1.2%<br>2.2%	
**Myeloradiculitis**	3.5%	
**Cerebral vasculitis**	1.2%	
**Myositis**	1.2%	
**Encephalomyelitis**		100%

Table 5: Hansen & Lebech, 1992 [24]

Clinical manifestation at the time of diagnosis	Acute (early) Lyme neuroborreliosis n=176	Late Lyme neuroborreliosis n=11
Lymphocytic meningoradiculitis with mononeuritis multiplex (Bannwarth syndrome)	61%	
Fascial paresis, unilateral	37%	
Fascial paresis, bilateral	17%	
Palsy, VI cranial nerve	5%	
Painful lymphocytic meningoradiculitis (without paresis)	24.6%	
Subacute lymphocytic meningitis (without pain, without paresis)	4.8%	
Myelo-meningoradiculitis	3.7%	
Chronic lymphocytic meningitis (with paresis, length of disease >6 months)		1.6%
Chronic progressive encephalomyelitis		4.3%

Table 6: (Oschmann et al., 1998 [15])

Symptoms related to Lyme neuroborreliosis	n=330
Paresis, peripheral	45%
Paresis, central	9%
Sensory disorder, peripheral	44%
Sensory disorder, central	4%
Cranial nerve palsy, fascial nerve	39%
Cranial nerve palsy, other	8%
Bladder paralysis	5%
Organic brain syndrome	3%
Parkinson’s disease	7%
Cerebellar ataxia	2%
Stroke	1.2%
Myositis	0.3%

Appendix 8

Table 7: GRADE assessment of the studies on antibiotics (modified according to [16])

**Quality assessment**						**Number of patients**		**Effects**		**Quality**
**No. of studies**	**Design**	**Risk of bias**	**Inconsistency**	**Indirectness**	**Imprecision**			**RR (95% CI)**	**Absolute**	
**Beta lactams vs. doxycycline**						**Beta lactams**	**doxycycline**			
Neurological symptoms after >4 months										
**3**	RCTs	Relevant[1]	No relevant inconsistencies	No relevant indirectness	Relevant[2]	59/105 (56.2%)	53/124 (42.7%)	RR 1.27 (0.98 – 1.63)	115 more per 1000 (from 9 fewer to 269 more)	Early manifestations:<br>++--<br>LOW<br>Late manifestations:<br>+---<br>VERY LOW<br>(additional indirectness)
Side effects										
**3**	RCTs	Relevant[1]	No relevant inconsistencies	No relevant indirectness	Relevant[2]	33/173 (19.1%)	35/169 (20.7%)	RR 0.94 (0.63 – 1.39)	12 fewer per 1000 (from 77 fewer to 81 more)	Early manifestations:<br>++--<br>LOW<br>Late manifestations:<br>+---<br>VERY LOW<br>(additional indirectness)
Neurological symptoms after >12 months										
**4**	RCTs	Relevant[1]	No relevant inconsistencies	No relevant indirectness	Relevant[2]	69/184 (37.5%)	3773/196 (32.7%)	RR 0.98 (0.76 – 1.26)	7 fewer per 1000 (from 89 fewer to 97 more)	Early manifestations:<br>++--<br>LOW<br>Late manifestations:<br>+---<br>VERY LOW<br>(additional indirectness)
[1] two unblinded studies, concerns regarding treatment allocation and selective reporting<br>[2] limited case numbers										
**Cefotaxime vs. penicillin**						**Penicillin**	**Cefotaxime**			
Neurological symptoms after >4 months										
**2**	RCTs	Relevant[1]	No relevant inconsistencies	No relevant indirectness	Relevant[2]	26/54 (48.1%)	16/60 (26.7%)	RR 1.81 (1.1 – 2.97)	216 more per 1000 (from 27 more to 525 more)	Early manifestations:<br>++--<br>LOW<br>Late manifestations:<br>+---<br>VERY LOW<br>(additional indirectness)

Quality assessment						Number of patients		Effects		Quality
**No. of studies**	**Design**	**Risk of bias**	**Inconsistency**	**Indirectness**	**Imprecision**			**RR (95% CI)**	**Absolute**	
Side effects										
**2**	RCTs	Relevant[1]	Relevant[3]	No relevant indirectness	Relevant[2]	22/79 (27.8%)	39/80 (48.8%)	RR 0.56 (0.38–0.84)	215 fewer per 1000 (from 78 fewer to 302 fewer)	+--- VERY LOW
[1] high risk of bias<br>[2] limited case numbers<br>[3] one study reported no findings										
**Combination of several antibiotics vs. single substance**						**Single substance**	**Combination**			
Neurological symptoms										
**2**	Observational study	Very serious[1]	No relevant inconsistencies	Relevant[2]	Relevant[3]	4/10 (40%)	2/8 (25%)	No pooling	No pooling	+--- VERY LOW
[1] high risk of bias<br>[2] various types of interventions, different treatment periods<br>[3] limited case numbers										
**Antibiotic treatment vs. no treatment**						**Treatment**	**No treatment**			
Neurological symptoms										
**3**	Observational studies	Very serious[1]	No relevant inconsistencies	No relevant indirectness	Relevant[2]	30/94 (31.9%)	31/79 (39.2%)	No pooling	No pooling	+--- VERY LOW
[1] high risk of bias<br>[2] limited case numbers										

Additional steroids vs. antibiotic monotherapy							Additional steroids	Antibiotic monotherapy		
**Quality assessment**							**No. of patients**		**Effects**	**Quality**
**No. of studies**	**Study design**	**Risk of bias**	**Inconsistency**	**Indirectness**	**Lack of precision**	**Other factors**	**Additional steroids**	**Monotherapy with antibiotics**		
**Residual fascial paresis following treatment (dichotomous, follow-up period: 12 months)**										
2	Observational studies	Very serious[1]	Serious[2]	Serious[3]	Serious[4]	None	53	44	Not pooled (see text)	⊕◯◯◯ Very low
**Residual symptoms of fascial paresis following treatment (follow-up period: 12 months; assessed using: eFACE Dynamic)**										
1	Observational studies	Very serious[1]	Serious[e]	Serious[f]	Serious[4]	None	18	17	See text	⊕◯◯◯ Very low
**Residual symptoms of fascial paresis following treatment (follow-up period: 12 months; assessed using: eFACE Synkinesis)**										
1	Observational studies	Very serious[1]	Serious[5]	Serious[6]	Serious[4]	None	18	17	See text	⊕◯◯◯ Very low
**Residual symptoms of fascial paresis following treatment (follow-up period: 12 months; assessed using: eFACE Composite)**										
1	Observational studies	Very serious[1]	Serious[5]	Serious[6]	Serious[4]	None	18	17	See text	⊕◯◯◯ Very low

[1] critical risk of bias in all included studies
[2] relevant differences in effect estimators
[3] very heterogenous patient population
[4] low case numbers
[5] single study
[6] intervention not sufficiently described

Appendix 9

Table 8: Important differential diagnoses of Lyme neuroborreliosis

Neurological manifestation	Differential diagnoses	Clinical characteristics, laboratory results
**Fascial paresis**	Idiopathic fascial paresis	▪ No CSF pleocytosis<br>▪ Differential diagnosis can be difficult in the early phase – especially in children
	Zoster oticus (Ramsay Hunt syndrome)	▪ Blisters on the external auditory canal (may be subtle or absent) and/or around the mouth<br>▪ Pain close to the ear<br>▪ Frequent hyperacusis and taste disorders<br>▪ Detection of virus in blisters and/or CSF<br>▪ VZV-AI
	Cranial polyradiculitis (Miller Fisher syndrome)	▪ Rarely unilateral, barrier dysfunction<br>▪ No CSF pleocytosis<br>▪ GQ1b antibodies<br>▪ Involvement of other cranial nerves
	Traumatic fascial paresis	▪ Patient history<br>▪ Imaging
	Fascial paresis related to tumour processes	▪ Cerebellopontine angle tumour<br>▪ Parotid gland tumour<br>▪ Neoplastic meningitis: patient history (gradual progression)<br>▪ CSF and imaging
	Mastoiditis, otitis media	▪ ENT results<br>▪ Imaging
	Bacterial meningitis of a different aetiology (incl. tuberculous meningitis)	▪ CSF<br>▪ Pathogen detection in CSF
	Sarcoidosis (Heerfordt syndrome)	▪ Fascial swelling with parotoid gland<br>▪ Uveitis<br>▪ Fascial paresis, often bilateral<br>▪ Imaging<br>▪ Serum marker for sarcoidosis
	Melkersson-Rosenthal syndrome	▪ Recurring<br>▪ Geographic tongue<br>▪ Fascial swelling
**Mono/ polyradiculitis (Bannwarth syndrome)**	Mono/polyradiculitis from other pathogens: VZV, EBV, HSV, CMV (the latter related to immunosuppression)	▪ Pathogen detection in CSF
	Herniated disc caused by root suppression, facet syndrome, ISG syndrome, piriformis syndrome	▪ Symptoms dependent on physical exertion<br>▪ Local mechanical triggering (or trigger points)<br>▪ Spinal imaging
	Spinal tumour (e.g. neurinoma, ependymoma); neoplastic meningitis	▪ Gradual progression<br>▪ Imaging
	Spondylodiscitis, spinal/dural abscess	▪ Mechanical local triggering<br>▪ Inflammation parameters<br>▪ Imaging
**Meningitis**	Chronic meningitis (pathogen-linked, no immunodeficiency):<br>▪ Mycobacterium tuberculosis<br>▪ Treponema pallidum<br>▪ Mollaret meningitis (HSV2)<br>▪ Parameningeal infection focus (sinusitis, mastoiditis, otitis)<br>▪ HSV 1 and 2<br>▪ Lymphocytic chorioretinitis<br>▪ Enteroviruses<br>▪ VZV (rare)	▪ Microbiological pathogen detection in CSF

Neurological manifestation	Differential diagnoses	Clinical characteristics, laboratory results
	Chronic meningitis (pathogen-linked, when immunodeficiency is present):<br>▪ HIV<br>▪ Mycobacterium tuberculosis<br>▪ CMV<br>▪ Cryptococcus neoformans<br>▪ Candida spp.<br>▪ Toxoplasma gondii	
	Chronic meningitis (not pathogen-linked):<br>▪ Neoplastic meningitis<br>▪ M. Behcet<br>▪ Connective tissue disease<br>▪ Sarcoidosis<br>▪ Migraines with CSF pleocytosis<br>▪ Chronic steroid-responsive idiopathic meningitis<br>▪ Drug-induced meningitis<br>▪ Leptomeningeal involvement with isolated CNS angiitis	▪ Microbiological differentiation of pathogen-linked causes in CSF and, if necessary, serum<br>▪ CSF cytology<br>▪ Autoimmune serology<br>▪ Clarification by internist/rheumatologist<br>▪ Drug history
**Myelitis**	Chronic myelitis (not caused by the pathogen):<br>▪ Chronic progressive multiple sclerosis<br>▪ Neuromyelitis optica (NMO) and neuromyelitis optica spectrum disorders (NMOSD)<br>▪ Connective tissue disease<br>▪ Paraneoplastic<br>▪ After vaccination (extremely rare)	▪ Microbiological differentiation of pathogen-linked cause in CSF and, if necessary, serum<br>▪ Spinal MRI<br>▪ Autoimmune serology<br>▪ Internal-rheumatological co-assessment<br>▪ Vaccination history
	Myelitis (caused by the pathogen):<br>▪ Mycobacterium tub.<br>▪ Treponema pallidum<br>▪ HSV, VZV, enteroviruses<br>If immunodeficiency is present:<br>▪ HIV, CMV, JCV<br>Parainfectious with:<br>▪ Mycoplasma pneumoniae<br>▪ HSV-2, VZV, CMV, EBV, adenovirus, ECHO, mumps	▪ Microbiological differentiation of pathogen-linked cause in CSF and, if necessary, serum<br>▪ Spinal MRI
	Chronic myelopathy:<br>▪ Spinal canal stenosis<br>▪ Funicular myelosis (vitamin B12 deficiency)<br>▪ Degenerative diseases (ALS, spastic spinal paralysis)<br>▪ Spinal dural arteriovenous fistulas<br>▪ Radiation myelopathy<br>▪ Adrenoleukodystrophy<br>▪ Hepatic myelopathy<br>▪ Copper deficiency myelopathy<br>▪ HIV myelopathy<br>▪ Spinal tumours<br>▪ Alcoholic myelopathy	▪ Spinal imaging (MRI, CT, myelography, angiography)<br>▪ Extended lab testing<br>▪ Clarification by internist<br>▪ CSF to differentiate from an infectious disease-related cause<br>▪ Electrophysiology (EPs)

Appendix 10

Table 9: Features of the RCT studies on treating post-treatment Lyme disease syndrome (PTLDS)

Study	Size	Case definition	Pre-treatment	Intervention	Treatment length	Country of origin
**Fallon 2008 [8]**	37	1. Status after erythema migrans or LB according to CDC criteria and positive or borderline ELISA with positive western blot<br>2. IgG western blot currently positive<br>3. Treatment with ceftriaxone i.v. for at least 3 weeks<br>4. Subjective memory impairment after onset of LB<br>5. Inconspicuous Wechsler Memory Scale III findings	At least 3 weeks of ceftriaxone i.v.	Ceftriaxone i.v. vs. placebo	10 weeks	USA
**Sjöwall 2012 [223]**	15	1. LNB according to EUCALB criteria<br>2. >6 months of persistent symptoms (fatigue, fascial paresis, headaches, radiculitis, cognitive impairments)	Ceftriaxone or doxycycline for at least 10–14 days	2 x 100 mg of doxycycline orally vs. placebo	3 weeks	Sweden
**Berende 2016 [221]**	280	1. Persistent symptoms attributed to LB (musculo-skeletal pain, arthritis/arthral-gia, neuralgia, sensory disorders, dysaesthesia, neuropsychological abnormalities, cognitive disorders, fatigue)<br>2. Time-related connection to erythema migrans or another clinically confirmed LB or positive serology	30–40 days of antibiotics, unclear which type	2 weeks of ceftriaxone, then 12 weeks of doxycycline vs. clarithromy-cin + hydroxy-chloroquine vs. placebo	12 weeks	Nether-lands
**Kaplan 2003 [220]**	129	1. Erythema migrans, early neurological/cardiological symptoms, radiculopathy, arthritis<br>2. Patient received antibiotic treatment	Previous antibiotics, length/type unclear	2g of ceftria-xone i.v. for 30d, then 200 mg of doxycyc-line orally for 60d vs. placebo i.v. for 30d, then placebo orally for 60d	90 days	USA
**Klempner 2001 [225]**	107	1. Erythema migrans, early neurological/cardiological symptoms, radiculopathy, arthritis<br>2. Patient received guideline-compliant antibiotic treatment<br>3. Persistent symptoms that occur within 6 months after LB and last >6 months, e.g. musculoskeletal pain, cognitive impairment, radiculopathy, paraesthesia/ dysaesthesia/fatigue<br><br>Seronegative + seropositive patients included	Previous antibiotic treatment, type unclear Median duration 50 days (placebo) – 66 days (verum)	2 weeks of 2g of ceftriaxone i.v. for 30d, then 2x100 mg of doxycycline orally for 60d vs. placebo i.v. for 30d, then placebo orally for 60d	90 days	USA

Study	Size	Case definition	Pre-treatment	Intervention	Treatment length	Country of origin
**Krupp 2003 [222]**	48	1. 18–70 years<br>2. Erythema migrans or late manifestation of LB according to CDC criteria with positive ELISA and western blot<br>1. Patient received guideline-compliant antibiotic treatment for 6 months before entering study<br>2. Currently experiencing fatigue	Minimum of 3 weeks of 2 x 100mg of doxycycline or 3 x 500 mg of amoxicillin or 2 g/d of ceftriaxone	2 g of ceftriaxone i.v. vs. placebo	28 days	USA
**Cameron 2008 [226]**	84	1. >18 years<br>2. Recurrence of LB symptoms after previously successful antibiotic treatment	All patients had received antibiotic treatment, length and type not stated	3 x 1000 mg of amoxicillin vs. placebo	3 months	USA
**Murray 2022 [224]**	29	1. >18 years<br>2. Clinical diagnosis of LB at least 6 months before entering study<br>3. Initial guideline-compliant antibiotic treatment<br>4. Persistent symptoms that started within 6 months after LB diagnosis which have persisted >6 months<br>5. Persistent pain and fatigue	Guideline-compliant pre-treatment was inclusion criterion, but was not specified	Kundalini yoga vs. waiting list	8 weeks	Nether-lands

Table 10: GRADE assessment: should antibiotics be used for post-treatment Lyme disease syndrome (PTLDS)?

Certainty assessment								Result	Certainty
**No. of studies**	**Study design**	**Risk of bias**	**Inconsistencies**	**Indirectness**	**Lack of precision**	**Antibiotics n=**	**Placebo n=**		
**Fatigue (various means of measurement)**									
3	Randomised clinical trials	Not serious	Serious[a]	Serious[b]	Serious[c]	228	132	Two RCTs found no statistically significant difference with regard to fatigue (including one study with an overall low risk of bias).<br>One RCT found less fatigue after antibiotic treatment.	⊕◯◯◯ Very low
**Depression (assessed using: BDI)**									
2	Randomised clinical trials	Not serious	Not serious[a]	Serious[b]	Serious[c]	84	77	No statistically significant difference with regard to depression.	⊕⊕◯◯ Low
**Quality of life (assessed using: SF36)**									
4	Randomised clinical trials	Not serious	Not serious	Serious[b]	Serious[c]	274	183	No statistically significant difference with regard to quality of life.	⊕⊕◯◯ Low
**Cognition (various means of measurement)**									
4	Randomised clinical trials	Not serious	Not serious	Serious[b]	Serious[c]	292	197	No statistically significant difference with regard to cognition.	⊕⊕◯◯ Low

Explanations
a. relevant heterogeneity
b. relevant difference in terms of case definition, intervention, treatment duration and follow-up period
c. pooled analysis not possible

Table 16: GRADE – additional administration of steroids for fascial paresis in the context of Lyme neuroborreliosis

Certainty assessment							Number of patients		Effects	Certainty
No. of studies	Study design	Risk of bias	Inconsistency	Indirectness	Lack of precision	Other factors	Additional steroids	Monotherapy with antibiotics		
**Residual fascial paresis following treatment (dichotomous, follow-up period: 12 months)**										
2	Observational studies	Very serious[a]	Serious[b]	Serious[c]	Serious[d]	None	53	44	Not pooled (see text)	⊕◯◯◯ Very low
**Residual symptoms of fascial paresis following treatment (follow-up period: 12 months; assessed using: eFACE Dynamic)**										
1	Observational studies	Very serious[a]	Serious[e]	Serious[f]	Serious[d]	None	18	17	See text	⊕◯◯◯ Very low
**Residual symptoms of fascial paresis following treatment (follow-up period: 12 months; assessed using: eFACE Synkinesis)**										
1	Observational studies	Very serious[a]	Serious[e]	Serious[f]	Serious[d]	None	18	17	See text	⊕◯◯◯ Very low
**Residual symptoms of fascial paresis following treatment (follow-up period: 12 months; assessed using: eFACE Composite)**										
1	Observational studies	Very serious[a]	Serious[e]	Serious[f]	Serious[d]	None	18	17	See text	⊕◯◯◯ Very low

a. critical risk of bias in all included studies according to ROBIN-I; b. relevant differences in effect estimators, two studies show no differences, one study shows downside for steroids; c. very heterogenous patient population; d. low case numbers; e. single study; f. intervention insufficiently described

Table 17: GRADE – Doxycycline for 2 weeks versus 6 weeks

Certainty assessment							Number of patients		Effects		Certainty
**No. of studies**	**Study design**	**Risk of bias**	**Inconsistency**	**Indirectness**	**Lack of precision**	**Other factors**	**Doxycycline for 2 weeks**	**Doxycycline for 6 weeks**	**Relative (95% CI)**	**Absolute (95% CI)**	
**Residual neurological symptoms (follow-up period: 12 months)**											
1	Randomised clinical trial	Not serious	Serious[a]	Not serious	Serious[b]	None	6/46 (13.0%)	10/46 (21.7%)	**RR 0.60** (0.24 to 1.51)	**87 fewer per 1,000** (from 165 fewer to 111 more)	⊕⊕◯◯ Low

a. single study
b. low case numbers

Table 18: GRADE – 2 weeks of antibiotic treatment versus protracted antibiotic treatment

Certainty assessment							Number of patients		Effects		Certainty
**No. of studies**	**Study design**	**Risk of bias**	**Inconsistency**	**Indirectness**	**Lack of precision**	**Other factors**	**2 weeks of antibiotics**	**Protracted antibiotic treatment**	**Relative (95% CI)**	**Absolute (95% CI)**	
**Residual neurological symptoms (follow-up period: 12 months)**											
2	Randomised clinical trials	Not serious	Not serious	Very serious[a]	Serious[b]	None	23/118 (19.5%)	24/121 (19.8%)	**RR 0.98** (0.59 to 1.64)	**4 fewer per 1,000** (from 81 fewer to 127 more)	⊕⊕◯◯ Low

a. heterogenous population, one study includes additional patients with non-neurological manifestations of Lyme borreliosis. Case definition not consequentially implemented in one study. Different types of antibiotics and treatment durations.
b. low case numbers, wide confidence interval

Table 19: GRADE efficacy of antibiotics in treating PTLDS

Certainty assessment								Result	Certainty
No. of studies	Study design	Risk of bias	Inconsistency	Indirectness	Lack of precision	Antibiotics n =	Placebo n =		
**Fatigue (various measurement tools)**									
3	Randomised clinical trials	Not serious	Serious[a]	Serious[b]	Serious[c]	228	132	Two RCTs found no statistically significant difference with regard to fatigue (including one study with an overall low risk of bias). One RCT found less fatigue following antibiotic treatment.	⊕◯◯◯ Very low
**Depression (assessed with: BDI)**									
2	Randomised clinical trials	Not serious	Not serious[a]	Serious[b]	Serious[c]	84	77	No statistically significant difference with regard to depression.	⊕⊕◯◯ Low
**Quality of life (assessed with: SF36)**									
4	Randomised clinical trials	Not serious	Not serious	Serious[b]	Serious[c]	274	183	No statistically significant difference with regard to quality of life.	⊕⊕◯◯ Low
**Cognition (various measurement tools)**									
4	Randomised clinical trials	Not serious	Not serious	Serious[b]	Serious[c]	292	197	No statistically significant difference with regard to cognition.	⊕⊕◯◯ Low

a. heterogeneity with respect to direction of outcome
b. relevant differences with regard to case definition, intervention, treatment duration and follow-up period
c. pooled analysis not possible

Table 20: GRADE side effects of antibiotics for PTLDS

Certainty assessment						Number of patients		Effects	Certainty
No. of studies	Study design	Risk of bias	Inconsistency	Indirectness	Lack of precision	Antibiotics	Placebo	Relative risk (95% CI)	
**Side effects (AE)**									
4	RCTs	Not serious	Not serious	Very serious[a]	Serious[b]	111/284 (39.1%)	47/192 (24.5%)	**RR 1.47** (1.11-1.95)	⊕⊕◯◯ Low
**Serious side effects (SAE)**									
4	RCTs	Not serious	Not serious	Very serious[a]	Serious[c]	7/289 (2.4%)	3/202 (1.5%)	**RR 1.51** (0.44-5.12)	⊕◯◯◯ Very low

[a] heterogeneity with respect to direction of outcome
[b] relevant differences with regard to case definition, intervention, treatment duration and follow-up period
[c] pooled analysis not possible
